# The Indian Pharmaceutical Landscape in 2025: CDSCO-Approved Small Molecules and Synthetic Approaches

**DOI:** 10.3390/molecules31213962

**Published:** 2026-11-03

**Authors:** Davide Benedetto Tiz, Luana Bagnoli, Francesca Marini, David C. Magri, Luca Sancineto

**Affiliations:** 1 Department of Chemistry, Faculty of Science, University of Malta, MSD 2080 Msida, Malta; david.magri@um.edu.mt; 2 Group of Catalysis and Organic Green Chemistry, Department of Pharmaceutical Sciences, University of Perugia, Via del Liceo 1, 06100 Perugia, Italy; luana.bagnoli@unipg.it (L.B.); francesca.marini@unipg.it (F.M.)

**Keywords:** CDSCO, small molecules, drug approval, therapies, India, pharmacovigilance, synthesis

## Abstract

In 2025, the Central Drugs Standard Control Organization (CDSCO) strengthened India’s pharmaceutical regulatory landscape through selective approval of novel drugs and intensified quality surveillance. Key approvals included tafamidis (amyloidosis), letermovir (post-transplant prophylaxis) and fexuprazan (erosive esophagitis). Concurrently, CDSCO flagged over 145 selected batches of drugs for failing quality standards, reflecting stricter enforcement of the New Drugs and Clinical Trials Rules (2019). This review discusses 20 small-molecule (<1000 Da) drugs approved in 2025 (from January to October) and the synthetic pathway for each therapeutic agent. Also, small molecules approved in the two previous years will be discussed. Peptides, radiopharmaceuticals, biologics, and inorganic compounds were excluded from the analysis. The review also highlights the most common impurities arising from the synthetic routes described and provides a comparative assessment of each drug against its main market competitors.

## 1. Introduction

The Central Drugs Standard Control Organization (CDSCO) in India is the national authority responsible for regulating drugs, medical devices, and clinical trials [1]. Drug control in India is mostly a post-Independence (1947) phenomenon. The Drugs and Cosmetics Act governs the import, manufacturing, and sale of allopathic, Ayurvedic, Unani, and homeopathic drugs and cosmetics [2]. Enforcing the Act’s provisions is the responsibility of both the central and state governments. While the Central Government oversees the quality of imported pharmaceuticals, establishes regulatory measures and standards, and so on, State Governments are responsible for monitoring drug manufacturing and sales.

The CDSCO grants the licenses for the manufacturing and marketing of pharmaceuticals. The Drugs Rules specify the basic requirements for granting licenses. Drug factories producing medications worth $1500 million are subject to frequent inspections by Drug Inspectors [3]. It is important to note that regulation does not stop at approval; it also involves post-marketing surveillance and quality control. The CDSCO released the New Drugs and Clinical Trials Rules (NDCTR) 2019, which marked a radical change in India’s clinical research regulations [4]. In some situations, such as for life-threatening illnesses, uncommon diseases, diseases with unmet medical needs, disasters, and specific defense usage, the new legislation allows for expedited medication development. There are two ways to do this: expedited review and speedy approval. Instead of using standard clinical outcomes like disease progression or mortality, a medicine may be approved for marketing under expedited approval based on surrogate endpoints [5]. Before permanent morbidity develops, the surrogate indicators should be detectable and able to forecast the course of the disease. Post-marketing clinical investigations that confirm the expected therapeutic benefit should come after approval [4]. The fact that the CDSCO flagged 145 drug batches as Not of Standard Quality (NSQ) in January 2025 serves as a reminder of the continuous oversight required [6]. Despite the growing relevance of the Indian pharmaceutical market and its increasing contribution to global drug innovation, no review article has been specifically devoted to systematically covering drugs approved in India. This represents a significant gap in the medicinal chemistry literature, as several compounds introduced into the Indian market subsequently gain international relevance or provide valuable insights into emerging therapeutic trends. To address this unmet need, the present review provides a comprehensive overview of the drugs approved for clinical use in India in 2025 and discusses their clinical applications, chemical–physical parameters and synthetic aspects. This effort is in line with our ongoing activity aimed at delivering timely reviews on newly approved drugs and emerging therapeutic classes [7,8,9,10,11,12].

## 2. CDSCO-Approved New Drugs in 2025

The CDSCO, India’s national regulatory authority for pharmaceuticals and medical devices, publishes a regular list of new drugs approved each year, as per the New Drugs and Clinical Trials Rules, 2019 (NDCTR) framework. For the year 2025 (period January–October, given that no drugs were approved in November and December), the registry lists a modest number of new drugs that have received approval for manufacturing or marketing. The CDSCO-approved 20 small molecules (<1000 Da) [13] will be discussed in the next section. There were eighteen separate entities approved. It should be noted that there was a combination of three drugs approved (fixed-dose combination of relugolix, estradiol, and norethindrone). In 2024, the CDSCO approved a total of 21 drugs, while in 2023 and 2022, there were 27 and 32 drugs approved, respectively [13]. While the preliminary numbers for 2025 indicate a decline, this often reflects a transition from quantity to quality, as discussed in the previous section. This strategic focus on quality over quantity will be further illuminated in the upcoming SAR, which aims to visualize these shifting analytical trends through the integration of PoseEdit sketches (when available).

**Figure 1:**
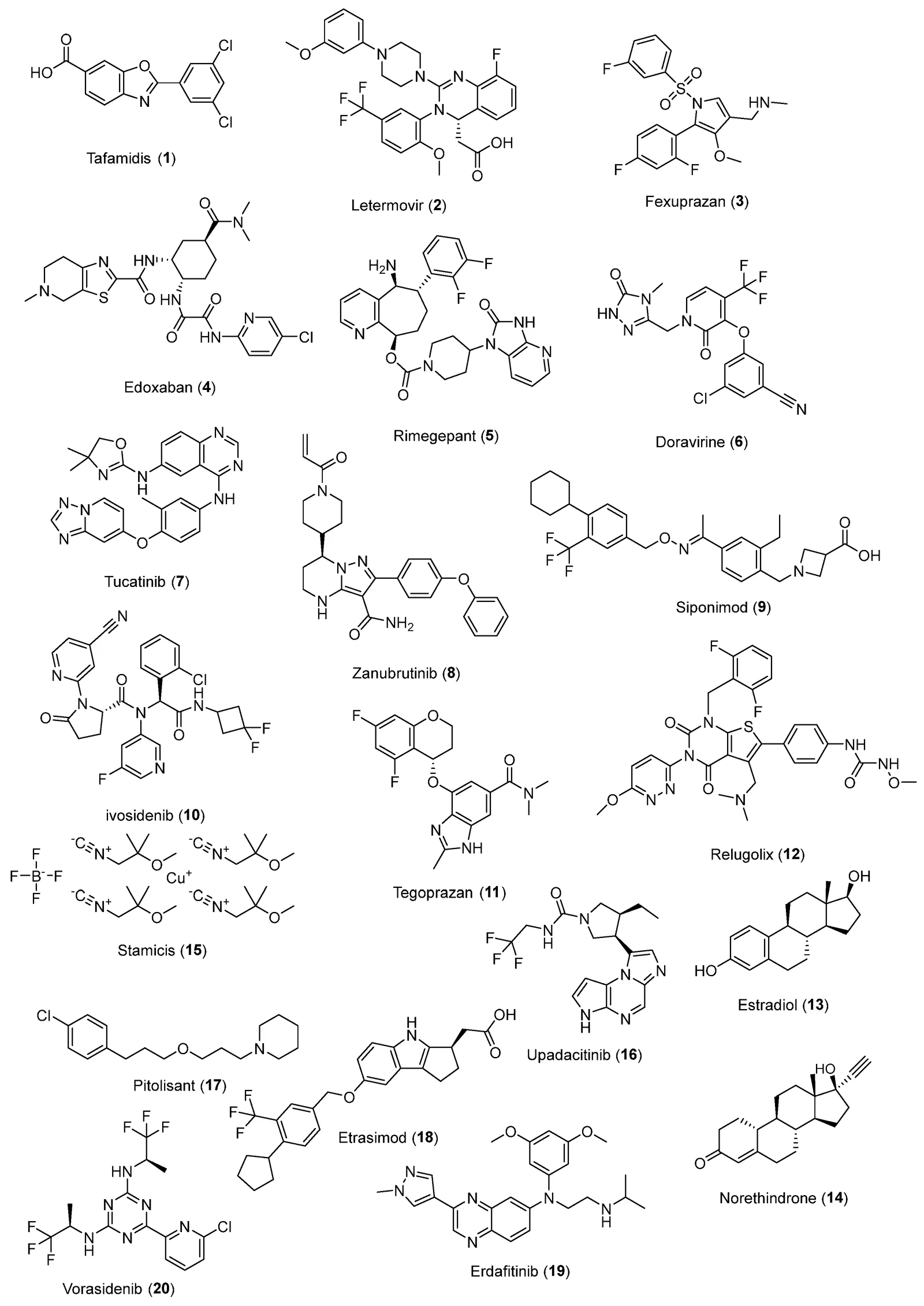
The chemical structures of CDSCO-approved new drugs in 2025.

PoseEdit automatically produces high-quality 2D diagrams of intermolecular interactions in 3D binding sites, calculated from ligands in complex with proteins, involving adjusting ligand orientation and conformation together with local protein features, such as side-chain rotamers, hydrogen positions, or flexible residues in the binding site, to resolve clashes, restore key interactions, and better represent induced-fit effects before further analysis or simulation [14]. In summary, PoseEdit binding is a predictive method for ligand–protein interactions, enabling accurate anticipation of binding conformations (pink/magenta dashed lines represent ionic interactions, dark-blue dashed line represents a standard hydrogen bond, light-blue/cyan dashed line with circles represents π–π stacking, green splines/smooth curves represent hydrophobic contacts). To evaluate the drug-likeness and pharmacokinetic profile of the studied compound, key physicochemical properties were assessed using computational methods. Specifically, topological polar surface area (TPSA) was utilized to predict membrane permeability, while the consensus Log P o/w (an averaged or combined estimate of the lipophilicity of a molecule) was calculated to estimate the lipophilic character of a molecule and absorption potential. Lipophilicity, measured as consensus Log P, is arguably the most critical physicochemical property in drug discovery because it determines the ability of the molecule to balance water solubility for blood transport with membrane permeability for cellular absorption. Poor absorption or permeation is more likely when the calculated Log P (CLogP) is greater than 5 [15]. In accordance with the drug-likeness filters described in the work of Veber (2022) [16], the target molecules demonstrate favorable membrane permeability, as their calculated TPSA values fall safely below the recommended threshold of 140 Å. Additionally, a synthetic accessibility score was employed to evaluate the feasibility of laboratory synthesis. Together, these parameters provide essential insight into the oral bioavailability and overall viability of a prospective drug candidate. Despite the inherent limitations of computationally predicted properties relative to experimentally validated data, integrating synthetic information and structure–property relationships of approved drugs with bioinformatics-based predictions may provide a valuable framework for the rational design of molecular analogs. This integrated approach could facilitate the identification of compounds with improved drug-like properties and greater synthetic accessibility. A detailed workflow for the computational calculations is reported in the Supporting Information. The chemical structures of the approved drugs are shown in Figure 1.

### 2.1. Tafamidis (***1***)

**Figure 2:**
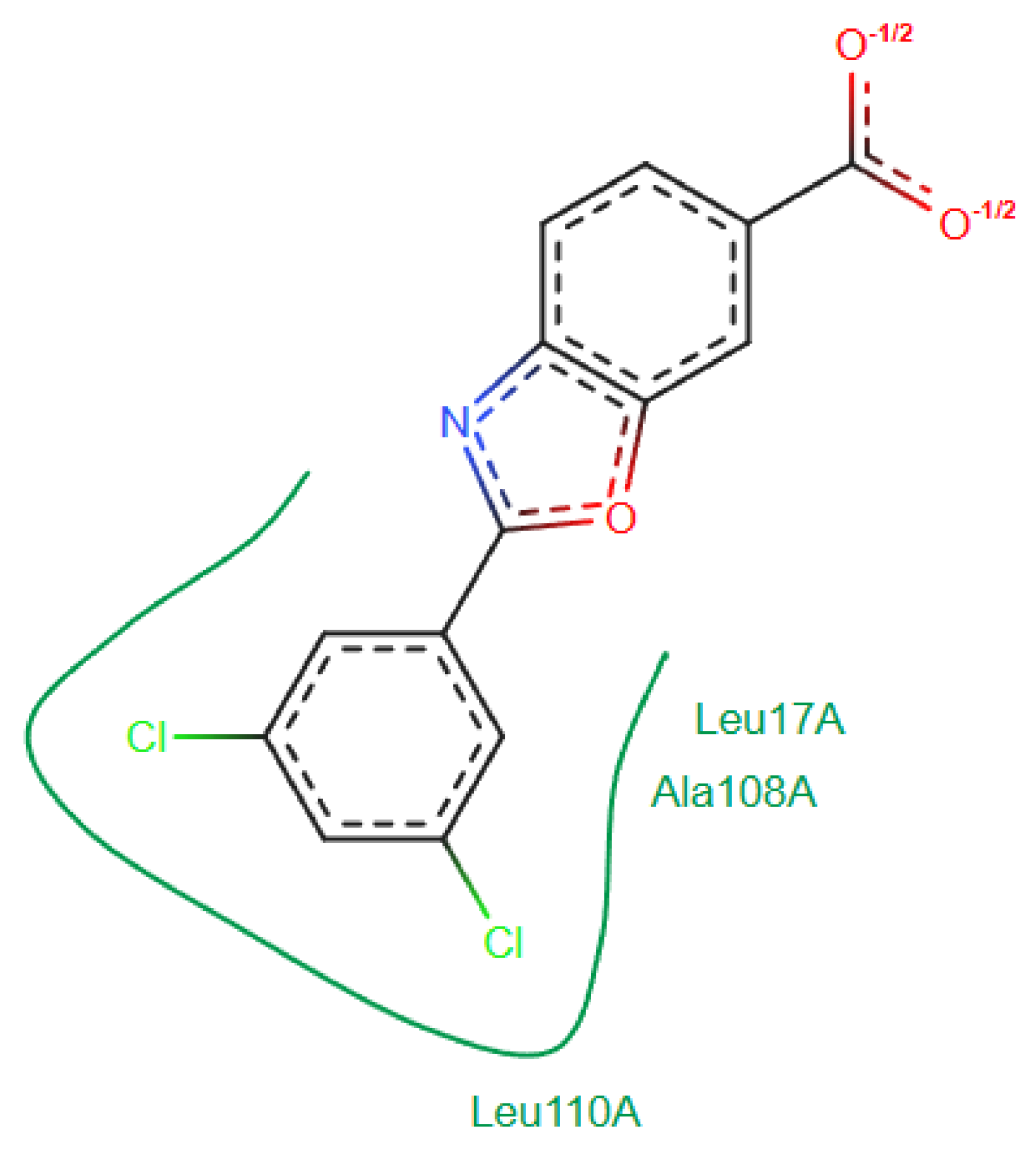
A 2D interaction map (PDB: 3TCT, crystal structure of wild-type TTR in complex with tafamidis) generated by PoseEdit [14].

On 16 January 2025, the CDSCO approved the bulk-drug/API for tafamidis, thereby adding the active substance of this treatment for transthyretin (TTR) amyloidosis to the list of new drug substances. Tafamidis, brand name Vyndaqel™ and Vyndamax™, is a drug intended to slow disease development in individuals with specific types of transthyretin amyloidosis [17,18]. It can treat both hereditary types, familial amyloid cardiomyopathy and familial amyloid polyneuropathy, as well as wild-type transthyretin amyloidosis, previously known as senile systemic amyloidosis. It functions by stabilizing the quaternary structure of the protein transthyretin. In persons with transthyretin amyloidosis, transthyretin breaks down and forms clumps termed amyloid that affect tissues including neurons and the heart [19]. Tafamidis represents a landmark example of structure-based drug discovery emerging from academic research. The compound was invented by Jeffery W. Kelly and colleagues at The Scripps Research Institute in the early 2000s, based on the principle of kinetic stabilization of transthyretin (TTR) tetramers to prevent amyloid fibril formation [20,21]. Chemically, it is made up of a benzoxazole core. This ring acts as a planar, aromatic bioisostere that can form strong π–π stacking interactions with aromatic residues in the TTR binding pocket. Tafamidis was identified as an oral candidate capable of substantially slowing the rate-limiting step in the TTR amyloid cascade and was selected for clinical development [22]. Chemically, it displays an intriguing 3,5-dichlorophenyl substituent. The presence of a halogen in drug candidates and approved drugs has already been discussed in comprehensive reviews [23,24]. Figure 2 illustrates a ligand–protein interaction map highlighting key hydrophobic contacts. The dichlorophenyl moiety (outlined in green) is positioned within a hydrophobic pocket, allowing for favorable van der Waals interactions with Leu17A, Ala108A, and Leu110A. These residues likely contribute significantly to binding affinity and ligand stabilization. The heteroaromatic core provides structural rigidity, while the polar substituents (e.g., the carboxylate group) may engage in additional electrostatic or hydrogen-bonding interactions outside the hydrophobic region, supporting overall binding specificity.

The electrostatic potential isosurface (Table 1) shows the molecule’s charge distribution. Areas of negative electrostatic potential (e.g., carboxylate) correspond to areas that will attract positive charges (electrophiles). The physicochemical properties (Table 1) of the compound, including topological polar surface area (TPSA, a medicinal chemistry descriptor measuring the sum of surfaces of all polar atoms, primarily oxygen, nitrogen, and attached hydrogens in a molecule), consensus Log P o/w [(consensus Log o/w is the arithmetic mean of multiple, diverse, in silico lipophilicity models (typically five) used to predict the partition coefficient of a compound between 1-octanol and water)], and synthetic accessibility, are described in the following paragraphs. The compound exhibits a TPSA value of 63.33 Å^2^, which is consistent with moderate polarity and suggests a favorable balance between hydrogen-bonding capacity and membrane permeability. The consensus Log P (o/w) of 3.76 indicates moderate lipophilicity, implying good potential for passive diffusion across biological membranes while avoiding excessive hydrophobicity that could compromise solubility. In addition, the synthetic accessibility score of 2.67 suggests that the compound is relatively easy to synthesize, with no major structural complexity that would hinder its practical preparation.

**Scheme 1:**
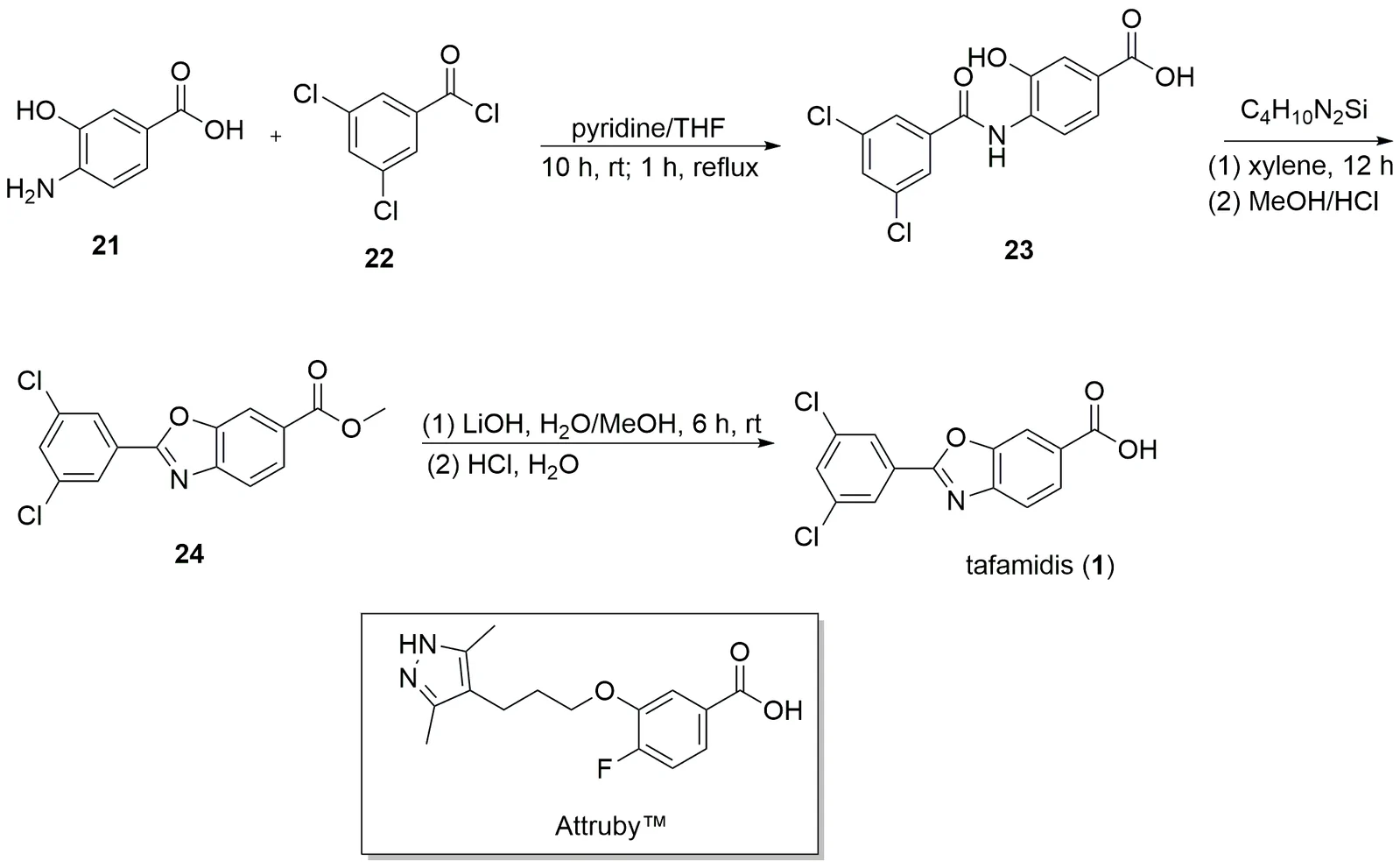
Synthetic scheme for tafamidis (**1**). Acoramidis (Attruby™) shown in the grey box.

The synthetic pathway shown in Scheme 1 [27], disclosed by Cellix Bio Private Limited, outlines the chemical preparation of the drug tafamidis. In the first step, an amide coupling reaction occurs between four-amino-three-hydroxybenzoic acid, labeled **21**, and 3,5-dichlorobenzoyl chloride, **22**, in a pyridine and tetrahydrofuran mixture to form the amide intermediate labeled **23**. Next, **23** undergoes cyclization and esterification using a silicon-containing reagent (trimethylsilyldiazomethane) in xylene, followed by a methanolic hydrogen chloride treatment. This step builds the core benzoxazole ring system to form the benzoxazole methyl ester intermediate (**24**).

Finally, a base-mediated ester hydrolysis of **24** using lithium hydroxide in aqueous methanol, followed by acidification with hydrochloric acid, yields the final target drug molecule, tafamidis. The specific reaction yields were not retrieved from the patent, as this data was not explicitly disclosed in the source text. A practical synthesis of tafamidis starting from commercially available 4-amino-3-hydroxybenzoic acid and 3,5-dichlorobenzaldehyde was described in 2026. The key transformation is a cyanide-catalyzed aerobic oxidative cyclization of an aldimine intermediate to form the benzoxazole core, with cyanide acting both as a catalyst and a base, affording tafamidis in high yield and purity [28]. *N*-Nitroso-meglumine (NNM) is a nitrosamine drug substance-related impurity associated with tafamidis meglumine and is considered a potential mutagenic and carcinogenic contaminant requiring stringent control [29]. Tafamidis is currently the established standard of care with proven long-term clinical experience, but its benefits may develop gradually, and it remains very costly. Acoramidis (Attruby™, Scheme 1) is a competitive drug to tafamidis, which showed particularly strong improvements in cardiovascular outcomes and biomarkers (NT-proBNP), suggesting potentially greater TTR stabilization [30].

### 2.2. Letermovir (***2***)

Approved on 17 January 2025, letermovir (Prevymis™) is an antiviral medication used to prevent cytomegalovirus (CMV) infection, mainly in CMV-positive adults who have undergone allogeneic hematopoietic stem cell transplantation [31]. It works by inhibiting the CMV terminase complex, which blocks viral DNA processing and packaging [32]. This mechanism is different from traditional CMV drugs like ganciclovir, which targets viral DNA polymerase. Letermovir has strong CMV-specific activity with minimal toxicity to human cells [33]. It is available in oral and intravenous forms and is generally well-tolerated, causing fewer blood-related side effects than older treatments [34,35]. Common adverse effects include nausea, diarrhea, fatigue, and headache [36]. The drug has notable interactions, especially with cyclosporine and other immunosuppressants [37]. Overall, it is an important option for CMV prevention in high-risk transplant patients and contributes to better clinical outcomes. Letermovir is a small-molecule antiviral compound belonging to the class of quinazoline derivatives. The molecule also includes multiple fluorine atoms that contribute to its selective binding to the cytomegalovirus (CMV) terminase complex. Thus, it exists in zwitterionic form at pH values 4.0 to 7.0, with a low intrinsic solubility of 0.3 mg/mL, which increases above pH 7.0 [38]. Chemically, it is characterized by both lipophilic aromatic rings and polar functional groups (e.g., a carboxylic acid group), which together support its antiviral activity and bioavailability. The isosurface mapping (Table 2) shows that there is a well-balanced distribution of positive and negative charges across the molecule. Letermovir displays a TPSA of 77.84 Å^2^, indicating a relatively high polar surface area that supports its capacity for hydrogen bonding while remaining within a range compatible with oral bioavailability. The consensus log P (o/w) value of 4.57 reflects pronounced lipophilicity, which is consistent with strong membrane permeability but may also be associated with lower aqueous solubility. Additionally, the synthetic accessibility score of 4.97 suggests moderate synthetic complexity, reflecting the presence of structurally elaborate features that may require multistep synthesis but remain feasible for large-scale production (Table 2).

**Scheme 2:**
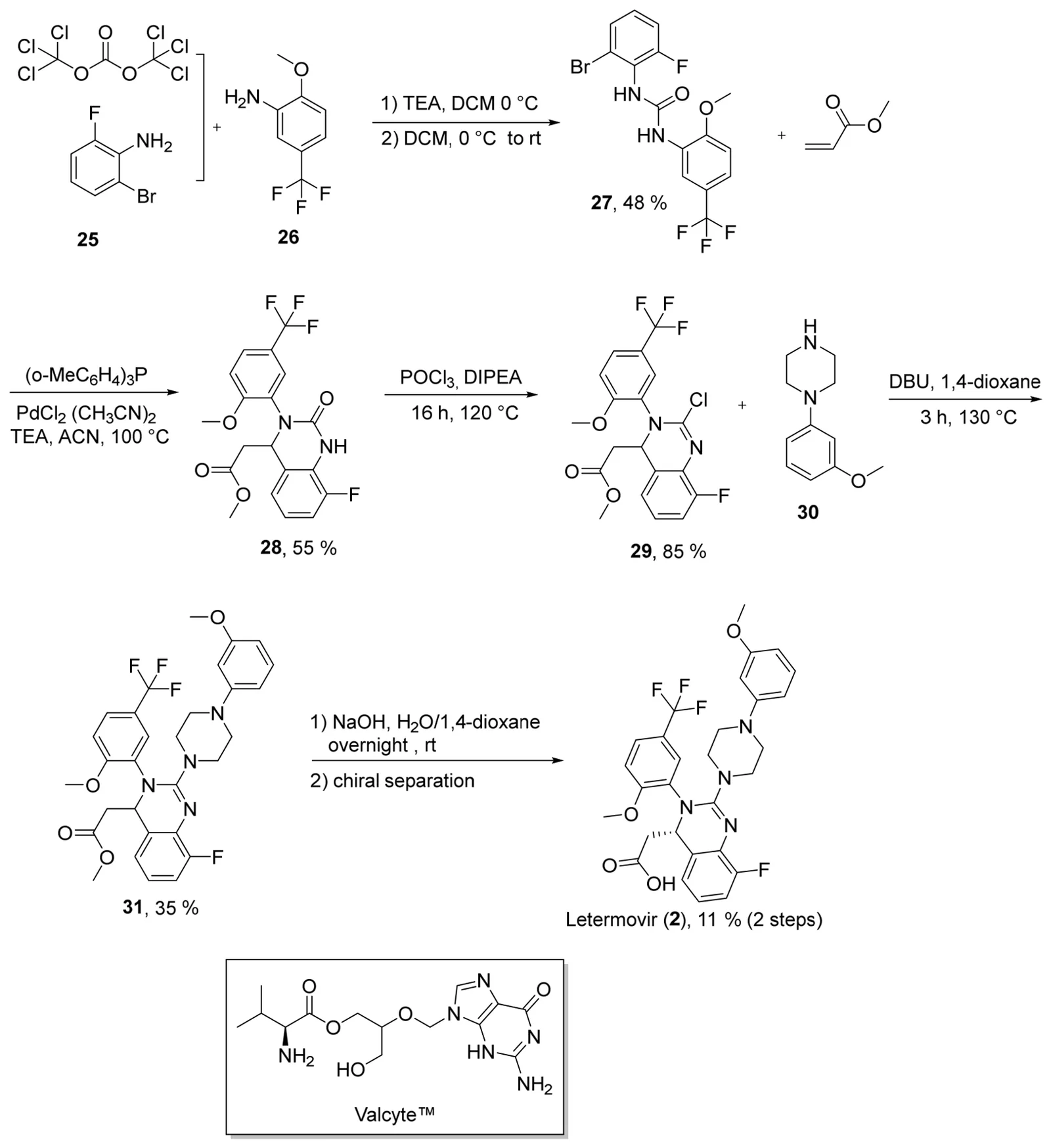
Synthetic scheme for letermovir (**2**). Valganciclovir (Valcyte™) shown in the grey box.

This inhibitor has been developed by Merck & Co., Inc., (Rahway, NJ, USA) under a global license from AiCuris Anti-infective Cures GmbH (GmbH, Wuppertal, Germany) [39]. The synthetic route for letermovir is shown in Scheme 2 [40], among other synthetic pathways. This strategy is highly convergent. By introducing the complex, heavily functionalized phenylpiperazine fragment (**30**) at a very late stage, they minimized the risk of carrying a costly and structurally sensitive amine through harsh reagents (like the hot phosphorus oxychloride chlorination step).

First, triphosgene mediates a coupling reaction between the substituted aniline intermediates **25** and **26** to form the urea intermediate **27** (48%). This urea intermediate then undergoes a palladium-catalyzed intramolecular Heck reaction and subsequent cyclization with methyl acrylate to build the quinazolinone core structure of intermediate **28** (55%). Next, intermediate **28** is chlorinated using phosphoryl chloride to produce the chloro-substituted intermediate **29** (85%), which then undergoes nucleophilic substitution with the piperazine derivative intermediate **30** to form intermediate **31** (35%). Finally, base-mediated ester hydrolysis of intermediate **31** followed by chiral separation (column, Chiralpak OD-H, 20 × 250 mm, 20 um; mobile phase: CO_2_/MeOH) provides the enantiopure target drug molecule (*S*)-letermovir in 11% yield. Moreover, researchers at Merck & Co have developed an efficient, seven-step asymmetric synthesis route [41] to produce the antiviral drug candidate letermovir, achieving an overall yield of greater than 60% from commercially available starting materials. Among several synthetic routes, a paper from 2017 reports the development of an enantioselective intramolecular aza-conjugate addition catalyzed by C_2_-symmetric bistriflamides, which activate α,β-unsaturated esters through dual hydrogen-bond donation. The method efficiently forms chiral 3,4-dihydroquinazolines (including letermovir), providing high yields and excellent enantioselectivities across a broad substrate scope [42]. Five main impurities were detected upon degradation of letermovir [43]. The first impurity is formed mainly by oxidation of the piperazine nitrogen (an *N*-oxide impurity). The second impurity is formed during thermal and photolytic degradation; it contains a newly formed olefinic (double-bond) structure. The third is formed by further oxidation of the second impurity. The fourth is formed through hydrolysis reactions. The fifth and last was observed particularly in letermovir injection after photolytic/oxidative stress. Letermovir appears to reduce CMV DNAemia during prophylaxis and is less likely to cause hematologic toxicity such as neutropenia and leukopenia, making it an attractive option for patients at risk of bone marrow suppression. However, the evidence is still relatively limited, with most data coming from observational studies, and long-term outcomes require further validation. Valganciclovir (Valcyte™, Scheme 2) remains the established standard prophylactic agent with extensive clinical experience, but its use is often limited by myelotoxicity, including neutropenia and leukopenia, which can complicate post-transplant management [44].

### 2.3. Fexuprazan (***3***)

Approved on 10 February 2025 (as hydrochloride), fexuprazan (Fexuclue™) is a potassium-competitive acid blocker (P-CAB) used to reduce gastric acid secretion. It works by reversibly inhibiting the H^+^/K^+^-ATPase proton pump in the stomach, offering faster and more sustained acid suppression than traditional proton pump inhibitors (PPIs) [45]. The drug is mainly used for treating gastroesophageal reflux disease (GERD), gastric ulcers, and other acid-related disorders [46]. Fexuprazan has a rapid onset of action and remains effective regardless of meal timing [47]. It is generally well-tolerated, with common side effects including headache, abdominal discomfort, and diarrhea. Compared with PPIs, it shows fewer drug–drug interactions and more consistent acid control. Overall, fexuprazan represents a newer, potent option for managing acid-related gastrointestinal conditions. Chemically, it contains a sulfonamide group and three fluorine atoms; particularly, it displays one monosubstituted and one disubstituted fluoroarene. While there are crystal structures for other potassium-competitive acid blockers (P-CABs) like vonoprazan (PDB ID: 5YLU), the specific structure involving fexuprazan has not been deposited in the public Protein Data Bank archive [48]. The isosurface mapping (Table 3) shows that the oxygen from the sulfonamide moiety could play an important role in binding given the high electron density. Fexuprazan displays a well-balanced physicochemical profile (Table 3) consistent with an orally bioavailable small-molecule drug. The TPSA of 68.71 Å^2^ supports adequate polarity for solubility while remaining compatible with efficient passive permeability. A consensus Log P (o/w) of 3.95 places the compound toward the upper end of acceptable lipophilicity, favoring membrane penetration and target engagement but warranting attention to potential metabolic and off-target liabilities. The synthetic accessibility score of 3.54 indicates good tractability, with no major concerns for synthesis or scale-up.

**Scheme 3:**
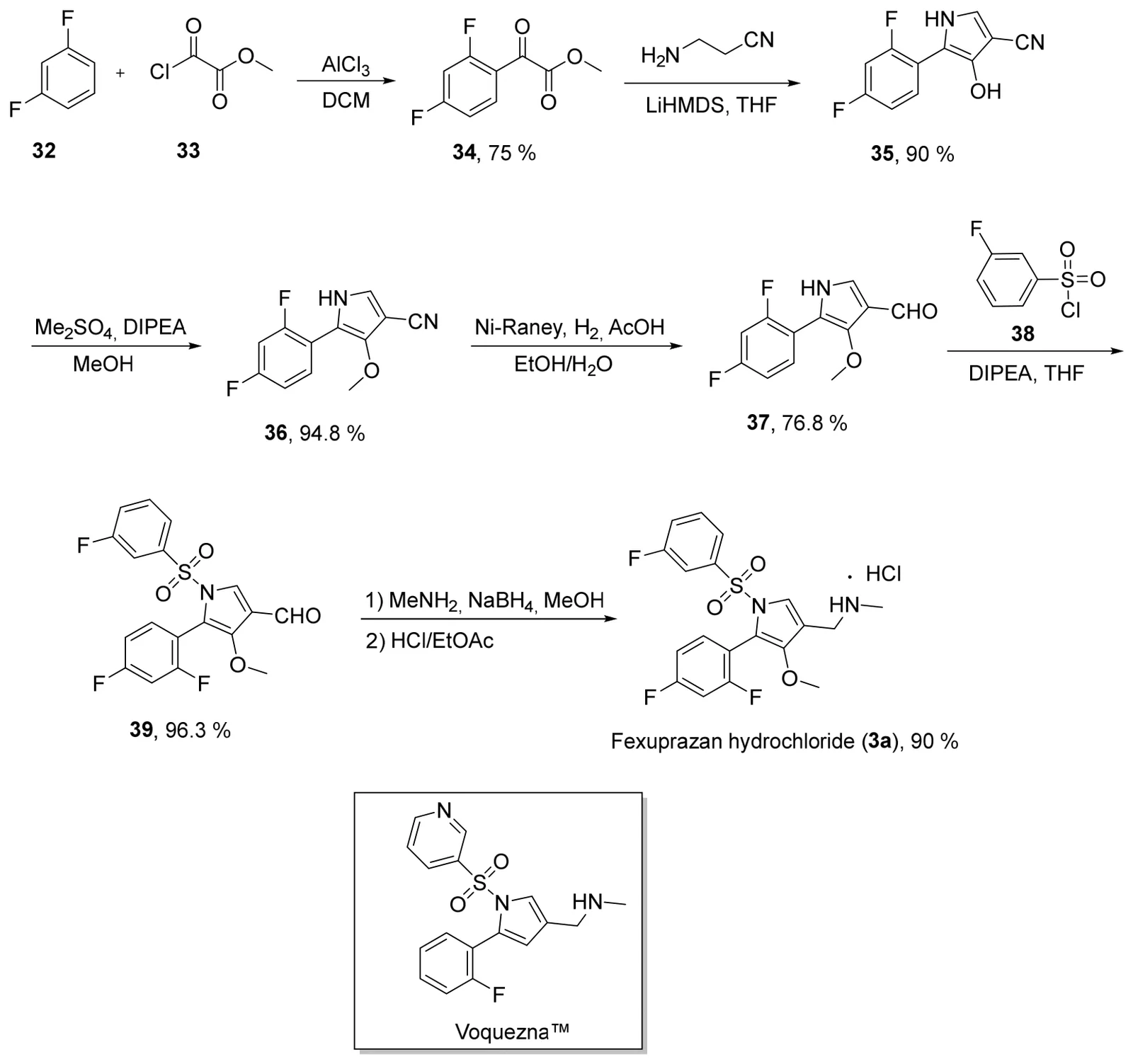
Synthetic scheme for fexuprazan hydrochloride (**3a**). Vonoprazan (Voquezna™) shown in the grey box.

It was developed by Daewoong Pharmaceutical Co., Ltd. (Seoul, Republic of Korea) [49]. A kilogram-scale process for fexuprazan is reported in Scheme 3 (by its manufacturer Chengda Pharmaceuticals, Jiaxing, China) [50]. In the first step, difluorobenzene (**32**) undergoes a Friedel-Crafts acylation with methyl oxalyl chloride (**33**) catalyzed by aluminum trichloride in dichloromethane to yield a substituted dicarbonyl compound (**34**, 75%). This intermediate is then subjected to a base-mediated cyclization with aminopropanenitrile using lithium bis(trimethylsilyl)amide in tetrahydrofuran to form a pyrrole ring containing both a nitrile group and a hydroxyl group (**35**, 90%). Next, the hydroxyl group on the pyrrole ring is selectively methylated using dimethyl sulfate and diisopropylethylamine in methanol to introduce a methoxy substituent (**36**, 94.8%). The nitrile group on this functionalized pyrrole is then partially reduced and hydrolyzed to an aldehyde (**37**, 76.8%) using a Raney nickel catalyst under a hydrogen atmosphere in a mixture of acetic acid, ethanol, and water. Following this transformation, the pyrrole nitrogen is sulfonylated with fluorobenzenesulfonyl chloride (**38**) in the presence of diisopropylethylamine in tetrahydrofuran to attach the arylsulfonyl group, affording **39** (96.3%). Finally, the aldehyde on **39** undergoes reductive amination with methylamine and sodium borohydride in methanol to yield a secondary amine, which is subsequently treated with hydrochloric acid in ethyl acetate to precipitate the final fexuprazan hydrochloride salt (**3a**, 90%). The overall yield was 41%. A new total synthesis of fexuprazan has been reported. Notably, most intermediates, as well as the final product, were isolated by simple recrystallization. The route afforded gram-scale fexuprazan in up to 47% overall yield [51]. Unlike the letermovir example, in this fexuprazan study, the impurities are process impurities/intermediates formed during synthesis and then recycled into the desired product []. Vonoprazan (Voquezna™, Scheme 3) has a larger body of clinical evidence and has demonstrated strong efficacy in symptom relief and mucosal healing, but long-term use has been associated with increased serum gastrin levels and gastric histological changes, although no malignant outcomes were reported. Fexuprazan also showed effective symptom control and mucosal healing with a favorable safety profile, and may offer an alternative for patients who do not respond adequately to PPIs. However, compared with vonoprazan, clinical experience and long-term safety data for fexuprazan remain more limited [52].

### 2.4. Edoxaban (***4***)

Approved on 11 February 2025, edoxaban (Savaysa™, Lixiana™, Roteas™) is an oral anticoagulant in the direct factor Xa inhibitor class, used to reduce the risk of stroke and systemic embolism in people with non-valvular atrial fibrillation and to treat or prevent deep vein thrombosis and pulmonary embolism [53]. It works by selectively blocking factor Xa, a key enzyme in the coagulation cascade, which decreases thrombin generation and blood clot formation [54]. After being taken once daily, it is absorbed quickly and reaches predictable blood levels, so routine INR monitoring is not needed [55]. Kidney function influences dosing because the drug is partially eliminated through the kidneys. Common side effects include bleeding, anemia, and gastrointestinal symptoms [56], and the risk of bleeding increases when combined with other anticoagulants, antiplatelet drugs, or certain medications that affect metabolism. Edoxaban is usually avoided in patients with mechanical heart valves or moderate-to-severe mitral stenosis, and dose adjustments may be required for low body weight, reduced kidney function, or the use of specific drug interactions [57]. Chemically, it contains a tertiary amide, a 6,7-dihydro-4*H*-[1,3]thiazolo[5,4-c]pyridine and a halogenated (chloro) arene. This combination of a rigid anchor and a flexible tail is a very common design in medicinal chemistry, allowing the core to fit snugly into a protein binding pocket while the tail explores the surrounding chemical space. Edoxaban is characterized by a high TPSA of 164.87 Å^2^ (Table 4), which is well above the classical threshold for optimal passive oral permeability. This elevated polarity is consistent with limited membrane diffusion and explains the reliance of the compound on transport-mediated absorption rather than simple passive uptake. The consensus Log P (o/w) of 1.34 indicates lower lipophilicity, favoring aqueous solubility and reducing the risk of nonspecific binding and metabolic liabilities, but further constraining permeability. The synthetic accessibility score of 5.04 suggests moderate synthetic complexity, reflecting the presence of multiple functional groups and stereochemical elements, yet remains acceptable for large-scale manufacture.

**Scheme 4:**
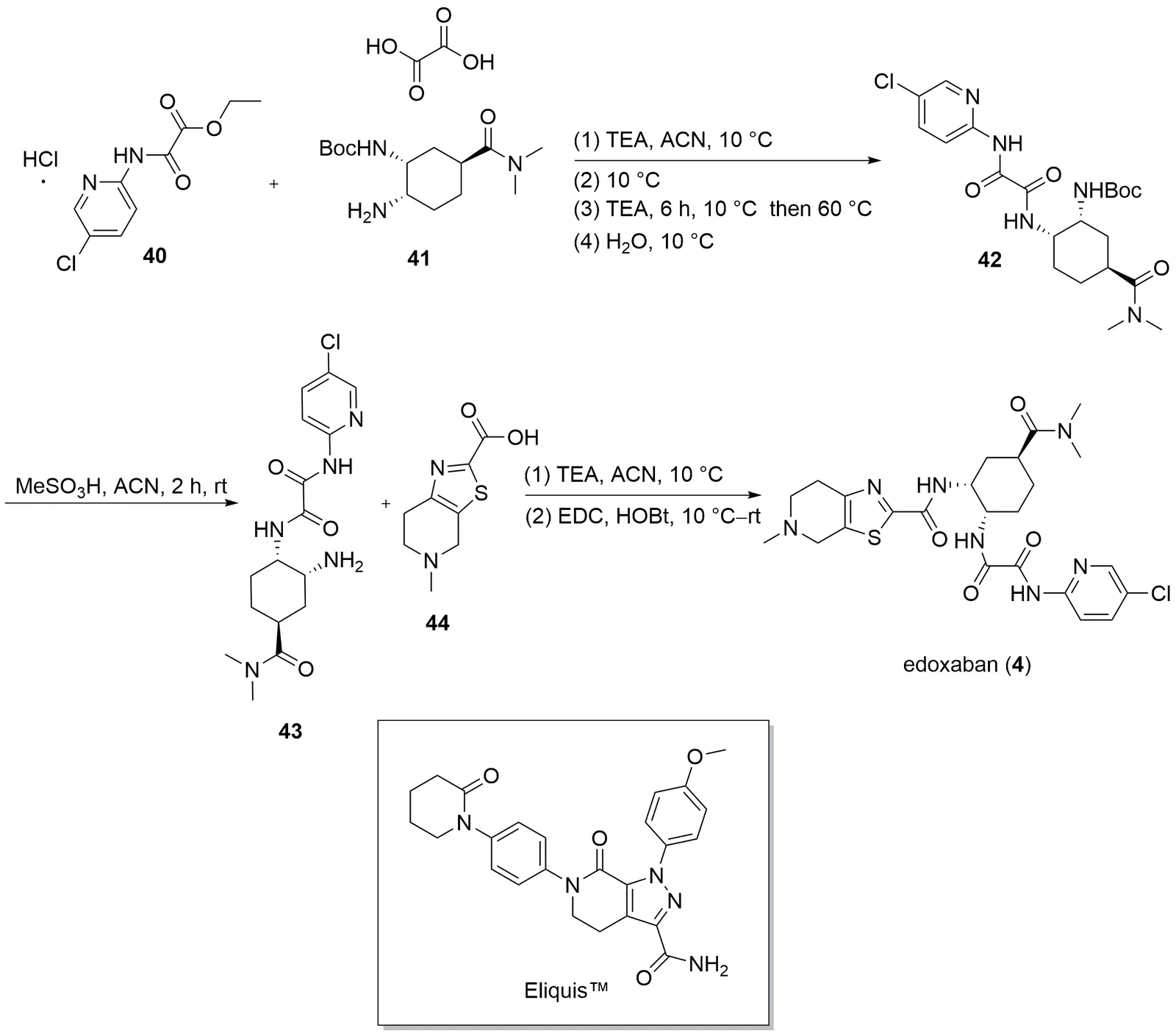
Synthetic scheme for edoxaban (**4**). Apixaban (Eliquis™) shown in the grey box.

It was marketed by Daiichi Sankyo [58]. Among the several synthetic routes for the synthesis of edoxaban, we report herein (Scheme 4) [59] a strategy that improves crystallization over other types of purification. In the first step, the ethyl oxamate derivative as its hydrochloride salt (**40**) undergoes an amidation reaction with the primary amine of the cyclohexane derivative, which is provided as its oxalate salt (**41**), in the presence of triethylamine in acetonitrile to build the central diamide core (**42**). Next, the tert-butyloxycarbonyl protecting group on the secondary amine is removed by treatment with methanesulfonic acid in acetonitrile at room temperature to liberate the free primary amine salt (**43**). Finally, this free amine is coupled with the carboxylic acid group of the tetrahydrothiazolopyridine derivative (**44**) using a base mixture of triethylamine and the coupling reagents 1-ethyl-3-(3-dimethylaminopropyl)carbodiimide (EDC) and hydroxybenzotriazole (HOBt) in acetonitrile to construct the final amide linkage and afford edoxaban (**4**). The impurities described in a recent paper are mirror-image or stereochemical variants of edoxaban that form accidentally during manufacture. They are chemically very similar to the desired drug but have a different three-dimensional arrangement, so they must be identified and controlled [60]. Edoxaban and apixaban (Eliquis™, Scheme 4) showed similar effectiveness and safety in elderly patients with atrial fibrillation. Edoxaban offers the convenience of once-daily dosing, while apixaban has broader clinical experience and remains more commonly used [61].

### 2.5. Rimegepant (***5***)

Approved on 27 March 2025, rimegepant (Nurtec ODT™) is a medication used both to treat migraine attacks when they occur and to prevent episodic migraines [62]. It works by blocking the CGRP receptor, a key pathway involved in migraine pain and inflammation [63]. The usual dose is 75 mg, taken either as needed for an attack or every other day for prevention [64]. It comes as an orally dissolving tablet that melts on the tongue [65]. Common side effects include nausea, stomach discomfort, and sleepiness, while serious reactions such as allergic responses are rare [64]. Rimegepant does not constrict blood vessels, making it an option for people who cannot take triptans, and it has a low risk of causing medication overuse headaches [66]. Chemically, it is a bicyclic, peptidomimetic compound designed to mimic key binding features of peptides while maintaining oral bioavailability. Rimegepant is not associated with a specific PDB entry for its primary target, the CGRP receptor, which is publicly available via a general search. More importantly, for a non-covalent inhibitor like rimegepant, binding affinity is not determined by the intrinsic reactivity of the molecule, but by its precise fit and complementary interactions within the binding pocket. This is achieved through a combination of shape complementarity and electrostatic interactions. The planar core acts as a rigid anchor that fits into the pocket, while the flexible tail can adjust to maximize contact. The carbamate oxygen displays a highly electronegative region, likely due to the donation of the nitrogen lone pair to the carbonyl group. Rimegepant combines a relatively high TPSA of 119.13 Å^2^, which may limit passive permeability, with a moderate lipophilicity (consensus Log P o/w = 3.10) that helps offset this polarity and supports oral absorption, while a synthetic accessibility score of 5.20 reflects appreciable structural complexity but remains manageable for development and manufacture (Table 5).

**Scheme 5:**
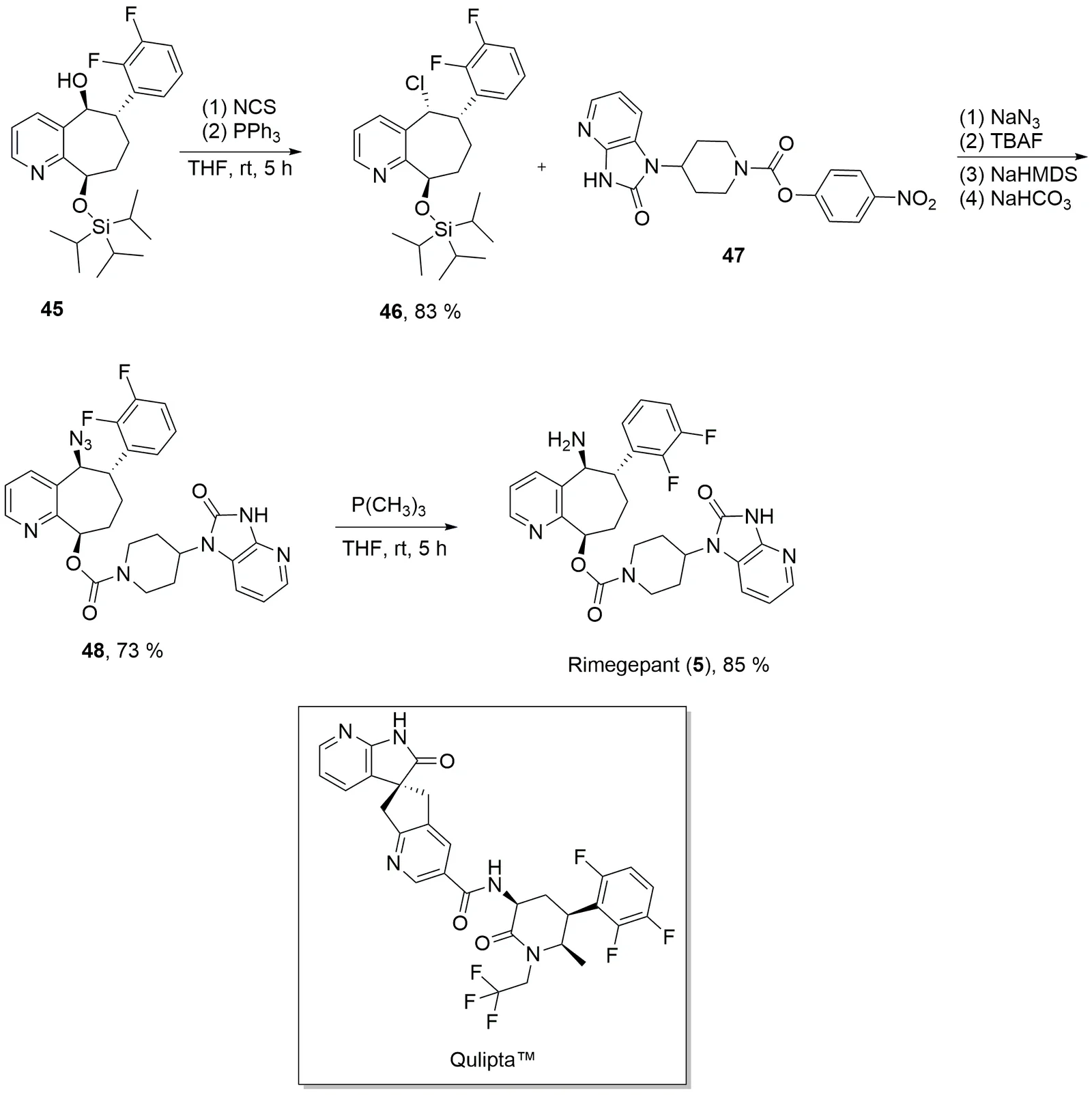
Synthetic scheme for rimegepant (**5**). Atogepant (Qulipta™) shown in the grey box.

Its synthesis (Scheme 5) [67] develops from the secondary alcohol of **45**, which undergoes Appel-type chlorination utilizing *N*-chlorosuccinimide and triphenylphosphine, which successfully converts the hydroxyl group into a chloride (**46**) with a complete inversion of stereochemistry in 83% yield. Next, **46** is converted to **48** (73%) through a multi-step sequence starting with nucleophilic substitution using sodium azide, which displaces the chloride and inverts the stereocenter once more to install the azide in its required configuration. This is followed by treatment with tetrabutylammonium fluoride to cleave the bulky silyl ether protecting group, revealing a free secondary alcohol. Sodium hexamethyldisilazide then deprotonates this newly formed alcohol, prompting it to attack the activated carbamate core of coupling partner **47**, which, after a mild sodium bicarbonate workup, delivers the carbamate-linked intermediate **48**. Finally, the azide group of **48** is selectively reduced to a primary amine via a Staudinger reduction using trimethylphosphine in THF, completing the total synthesis to afford rimegepant (**5**, 85%) with fully preserved stereochemical integrity. The overall yield was 51%. Three *N*-oxide degradation products and two hydrolysis degradation products were detected in a recent study [68]. Rimegepant and atogepant (Qulipta™, Scheme 5) are effective CGRP receptor antagonists for migraine management. Rimegepant offers flexibility for both acute and preventive treatment, while atogepant is specifically approved for prevention and may provide stronger reductions in monthly migraine days. Both are generally well-tolerated, although long-term experience is greater with rimegepant [69].

### 2.6. Doravirine (***6***)

**Figure 3:**
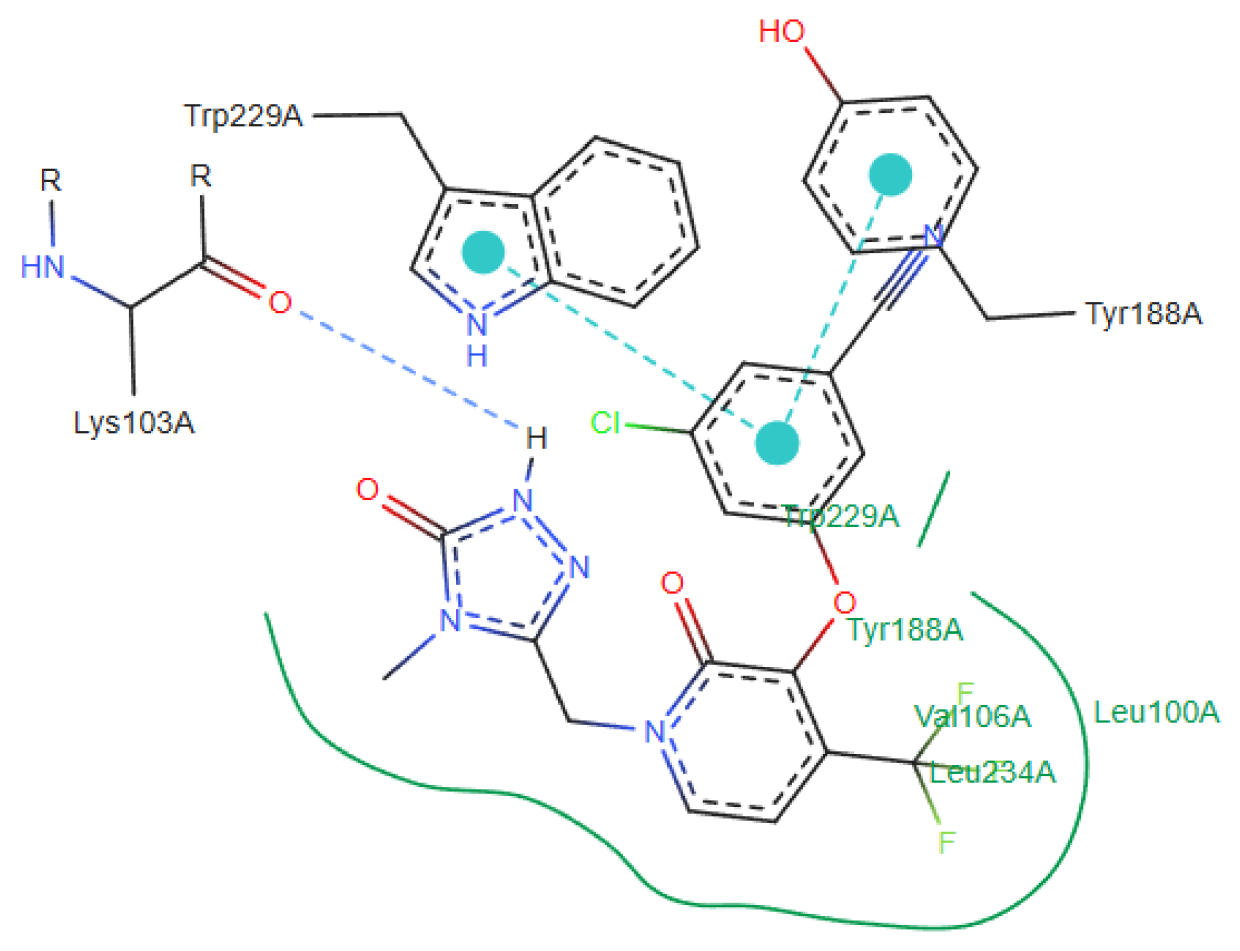
2D interaction map (PDB: 7Z2H, cryo-EM structure of NNRTI-resistant M184I/E138K mutant HIV-1 reverse transcriptase with a DNA aptamer in complex with doravirine) generated by PoseEdit [14].

Approved on 28 March 2025, doravirine (Pifeltro™) is an antiretroviral medication used to treat HIV-1 infection. It belongs to the class of non-nucleoside reverse transcriptase inhibitors (NNRTIs) and works by blocking the activity of the reverse transcriptase enzyme, which HIV needs to replicate [70]. It is typically taken once daily, either alone or as part of fixed-dose combination tablets with other antiretrovirals [71]. Doravirine is generally well-tolerated, with common side effects including nausea, headache, dizziness, and fatigue [72]. It has fewer central nervous system effects and fewer drug interactions than some older NNRTIs [73]. Doravirine is usually prescribed as part of combination therapy to maintain viral suppression and improve immune function in people living with HIV [74]. The molecule contains several key structural features: a pyridinone ring system that forms the central scaffold, a trifluoromethyl group and a chlorobenzyl moiety that likely contributes to hydrophobic interactions. The diagram (Figure 3) depicts the binding mode of the ligand within the protein active site, highlighting both polar and hydrophobic interactions. The ligand forms key hydrogen bonds with Lys103A and π–π interactions with Trp229A, stabilizing the heterocyclic core and anchoring the molecule within the binding pocket. Additional aromatic and π–π interactions are observed near Tyr188A, contributing to ligand orientation. The hydrophobic substituent (outlined in green) occupies a nonpolar pocket formed by Leu100A, Val106A, Leu234A, and Tyr188A, suggesting favorable van der Waals contacts that enhance binding affinity. Overall, the interaction pattern indicates a balance between specific polar recognition and hydrophobic packing, which likely plays a crucial role in ligand potency and selectivity.

The oxo-triazole ring displays a highly electropositive region due to the hydrogen bonding of one nitrogen atom (Table 6). This positive charge favors the interaction with oxygen’s lone pairs from Lys103A. Doravirine exhibits a balanced physicochemical profile (Table 6), with a TPSA of 105.70 Å^2^ that sits near the upper limit for efficient passive permeability, a moderate consensus Log P (o/w) of 2.75 supporting oral absorption without excessive lipophilicity, and a low synthetic accessibility score of 3.28 indicative of good synthetic tractability and favorable developability.

**Scheme 6:**
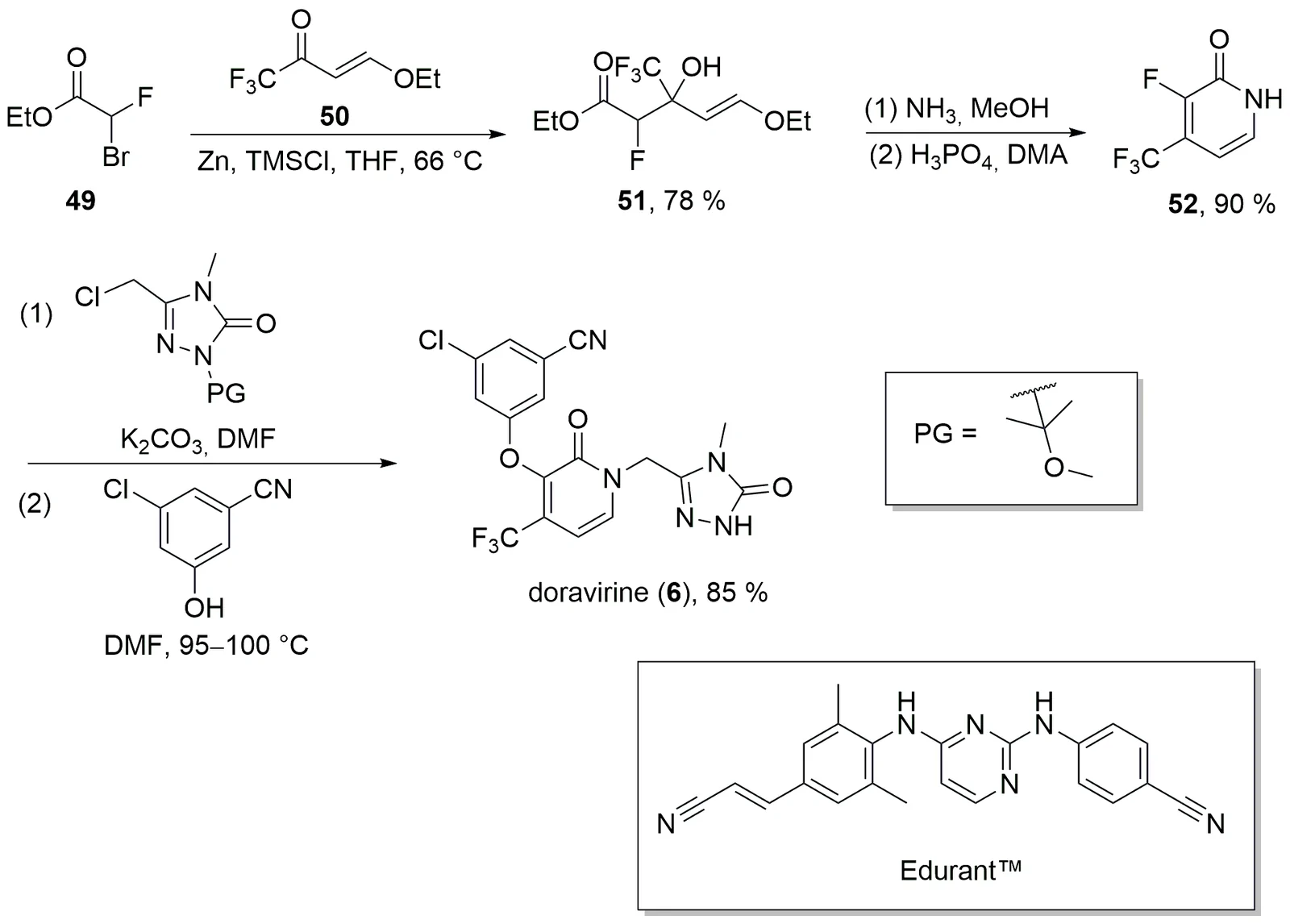
Synthesis of doravirine (**6**). Protecting group MIP (methoxyisopropyl group) shown in the grey box. Rilpivirine (Edurant™) shown in the grey box.

The synthetic approach (Scheme 6), directly from the inventors at Merck [75], involves a Reformatsky reaction between alpha-halo ester **49** and chalcone **50** in the presence of metallic zinc and TMSCl to yield a beta-hydroxy ester **51** (78%). Following amination, cyclization, and dehydration to form 3-fluoro-4-(trifluoromethyl)pyridone **52**, doravirine (**6**) was accessed via a multi-step sequence highlighted by a protection–deprotection (MIP group, methoxyisopropyl group) strategy to install the triazolinone and the phenolic group [76]. The nucleophilic aromatic substitution involving 3-chloro-5-hydroxybenzonitrile requires the use of a base such as K_2_CO_3_ (not reported in the literature). 85% yield was reported for the final step [75]. The overall yield was 60%. Several contaminants are formed during the manufacture of doravirine. During the workup and purification of doravirine, two low-level impurities are generated through the sensitive nitrile group [77]. Rilpivirine (Edurant™, Scheme 6) and doravirine have complementary activity against HIV strains with NNRTI resistance mutations. Rilpivirine appears more effective against a broader range of resistant mutants, while doravirine retains activity against several mutations that reduce rilpivirine efficacy [78].

### 2.7. Tucatinib (***7***)

Approved on 8 April 2025, tucatinib (Tukysa™) is an oral targeted anticancer drug used primarily to treat HER2-positive breast cancer (HER2 is a member of the human epidermal growth factor receptor), including cases that have spread to other parts of the body or to the brain [79]). It is a highly selective inhibitor of the HER2 tyrosine kinase, meaning it blocks the signaling pathways that drive tumor cell growth and survival while sparing the related HER1/EGFR pathway [80], which helps reduce some side effects seen with less selective drugs. Tucatinib is usually given in combination with trastuzumab and capecitabine, forming a regimen that has been shown to improve survival and control brain metastases in patients with advanced HER2-positive disease [81]. Common adverse effects include diarrhea, fatigue, nausea, and hepatotoxicity, so liver function monitoring is important during treatment [82]. Overall, tucatinib provides a more targeted and effective option for patients with difficult-to-treat HER2-positive cancers. Its structure is built around a substituted aminopyrimidine core, a common scaffold in kinase inhibitors [83]. Tucatinib presents a polarity–lipophilicity balance (Table 7) typical of targeted kinase inhibitors, with a TPSA of 110.85 Å^2^ approaching the upper limit for passive permeability, a relatively high consensus Log P (o/w) of 3.77 that compensates for this polarity and supports membrane penetration, and a synthetic accessibility score of 4.01 consistent with moderate structural complexity yet acceptable manufacturability.

**Scheme 7:**
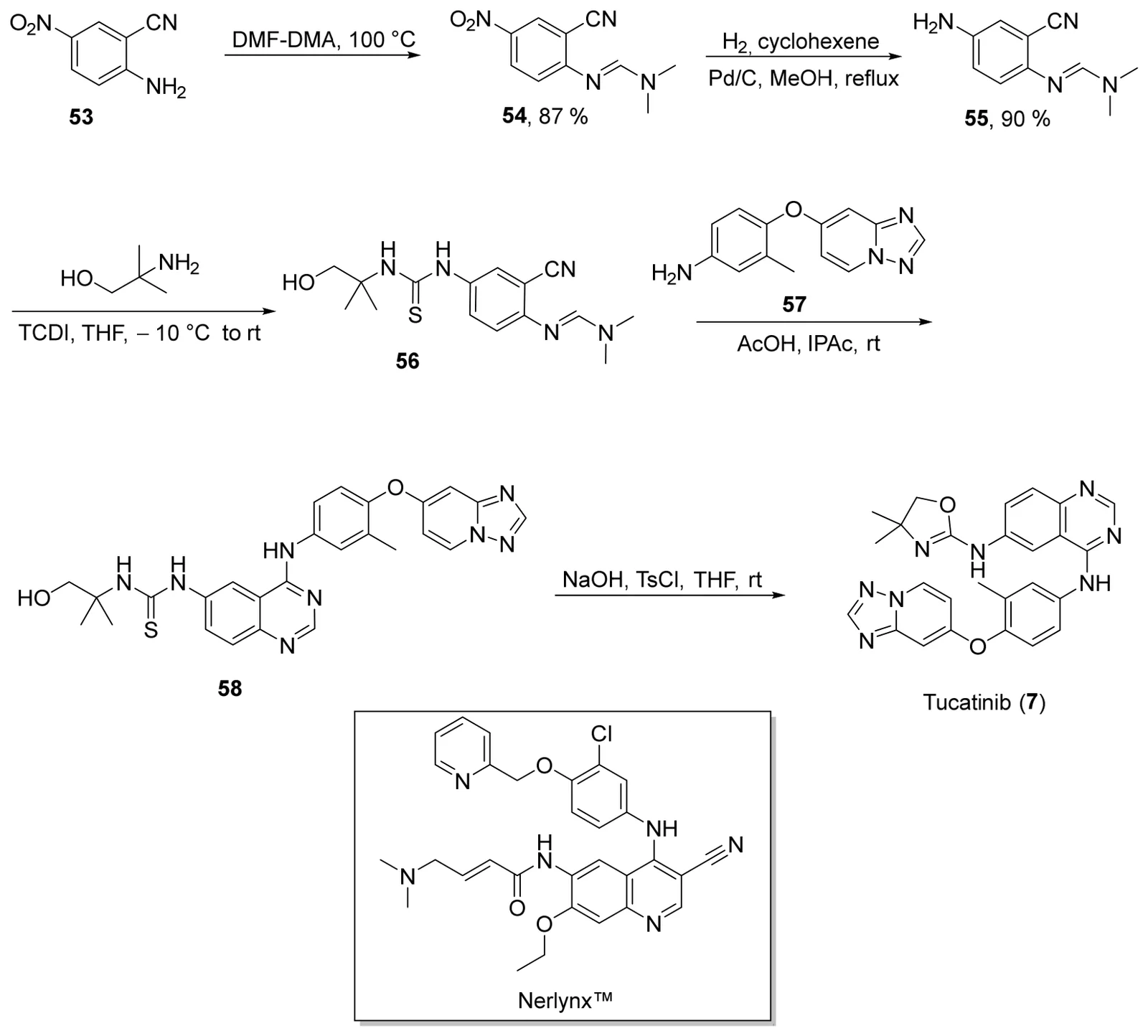
Synthesis of tucatinib (**7**). Neratinib (Nerlynx™) shown in the grey box.

The synthesis [84] is shown in Scheme 7. The process begins with the treatment of the substituted benzonitrile **53** with dimethylformamide dimethyl acetal at an elevated temperature to form the formamidine intermediate **54** (87%), which subsequently undergoes catalytic transfer hydrogenation using cyclohexene and palladium on carbon to selectively reduce the nitro group, yielding the aniline intermediate **55** (90%). This resulting amine is then coupled with an amino alcohol in the presence of thiocarbonyldiimidazole (TCDI) to yield the thiourea intermediate **56**. Next, **56** undergoes acid-mediated condensation and cyclization with the bicyclic ether-linked aniline **57**, constructing the central quinazoline core found in **58**. Finally, the sulfur of **58** is activated and cyclized intramolecularly using sodium hydroxide and tosyl chloride to close the oxazoline ring (cyclodesulfurization), completing the synthesis of tucatinib. Due to its aromatic nitro functionality present in the starting material, the related impurities were considered potential genotoxic risks and therefore required monitoring at trace levels [85]. Neratinib (Nerlynx™, Scheme 7) and tucatinib are both effective HER2-targeted tyrosine kinase inhibitors, but tucatinib has shown stronger efficacy in metastatic disease, particularly in patients with brain metastases, with significant improvements in progression-free and overall survival.

### 2.8. Zanubrutinib (***8***)

**Figure 4:**
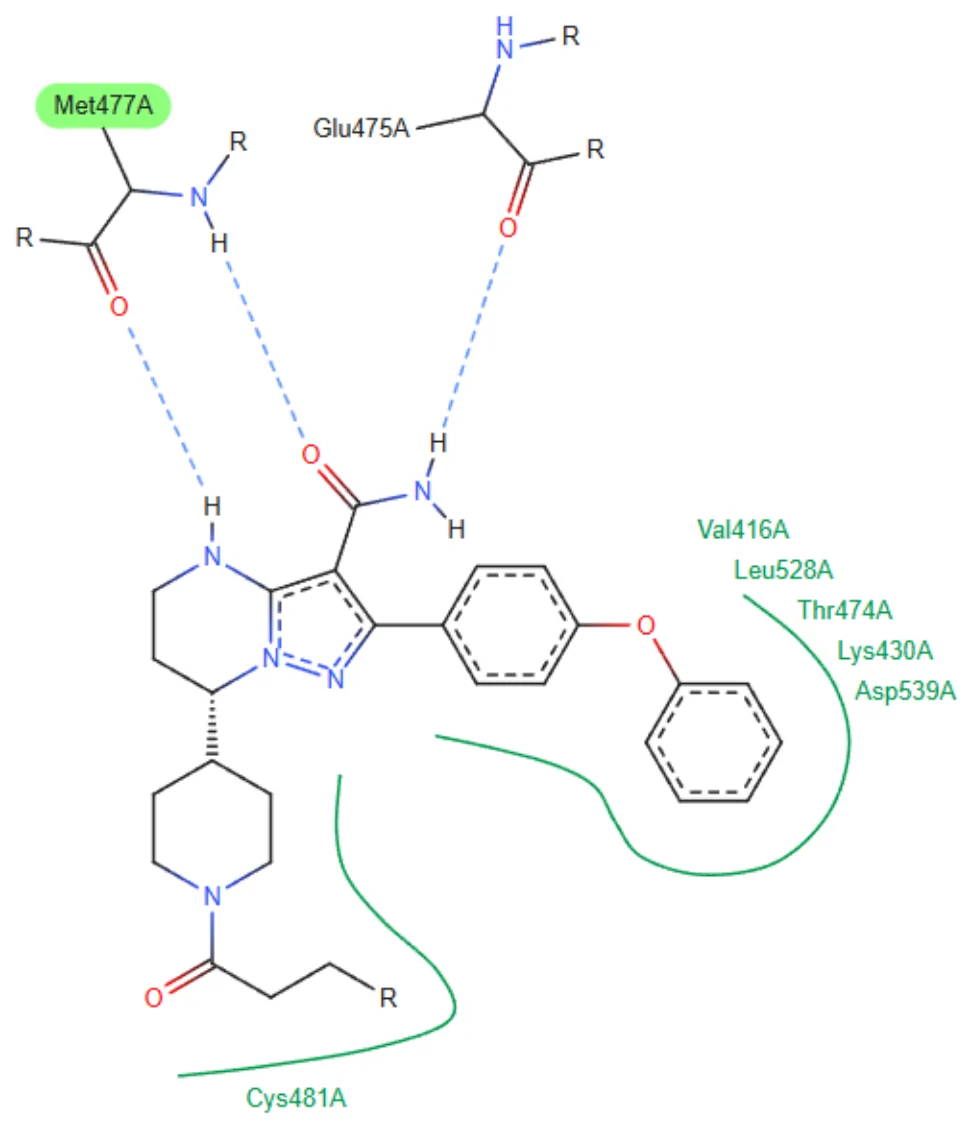
A 2D interaction map (PDB: 6J6M, co-crystal structure of BTK kinase domain with zanubrutinib) generated by PoseEdit [14].

Approved on 8 April 2025, zanubrutinib (Brukinsa™) is an oral medication that inhibits Bruton’s tyrosine kinase (BTK), an enzyme essential for the growth and survival of certain B-cell cancers [86]. It is used to treat conditions like mantle cell lymphoma, chronic lymphocytic leukemia, and Waldenström’s macroglobulinemia, especially when other treatments are unsuitable [87]. By blocking BTK, zanubrutinib slows the proliferation of malignant B-cells. Taken in capsule form, it is designed to be more selective, potentially reducing side effects [88]. Common side effects include infections, bleeding, diarrhea, and low blood cell counts, requiring careful monitoring. Chemically, it is a covalent inhibitor since it binds irreversibly to a cysteine residue (Cys481) [89] in BTK via Michael addition, thanks to its electrophilic acrylamide moiety. Figure 4 illustrates the ligand binding interactions within the active site, emphasizing a network of hydrogen bonds and hydrophobic contacts. The central heterocyclic scaffold engages in multiple hydrogen bonds with Glu475A and Met477A, providing strong anchoring and orientational stability. Additional polar interactions further stabilize the binding pose along the backbone amide region. The aryl substituent extends into a hydrophobic pocket (outlined in green) formed by Val416A, Leu528A, and Thr474A, suggesting favorable van der Waals interactions that contribute to binding affinity. The lower aliphatic moiety interacts near Cys481A, potentially enhancing pocket occupancy and overall ligand stabilization. Collectively, these interactions indicate a balanced contribution of specific polar recognition and hydrophobic packing to ligand binding.

This is confirmed by the isosurface mapping (Table 8), which clearly shows an electron-rich region corresponding to the acrylamide oxygen. Zanubrutinib shows a well-tuned physicochemical profile (Table 8), with a TPSA of 102.48 Å^2^ compatible with oral exposure despite being near the upper permeability threshold, a moderate-to-high consensus Log P (o/w) of 3.17 that supports membrane permeability and target engagement, and a synthetic accessibility score of 4.32 reflecting moderate complexity but feasible synthesis for development.

**Scheme 8:**
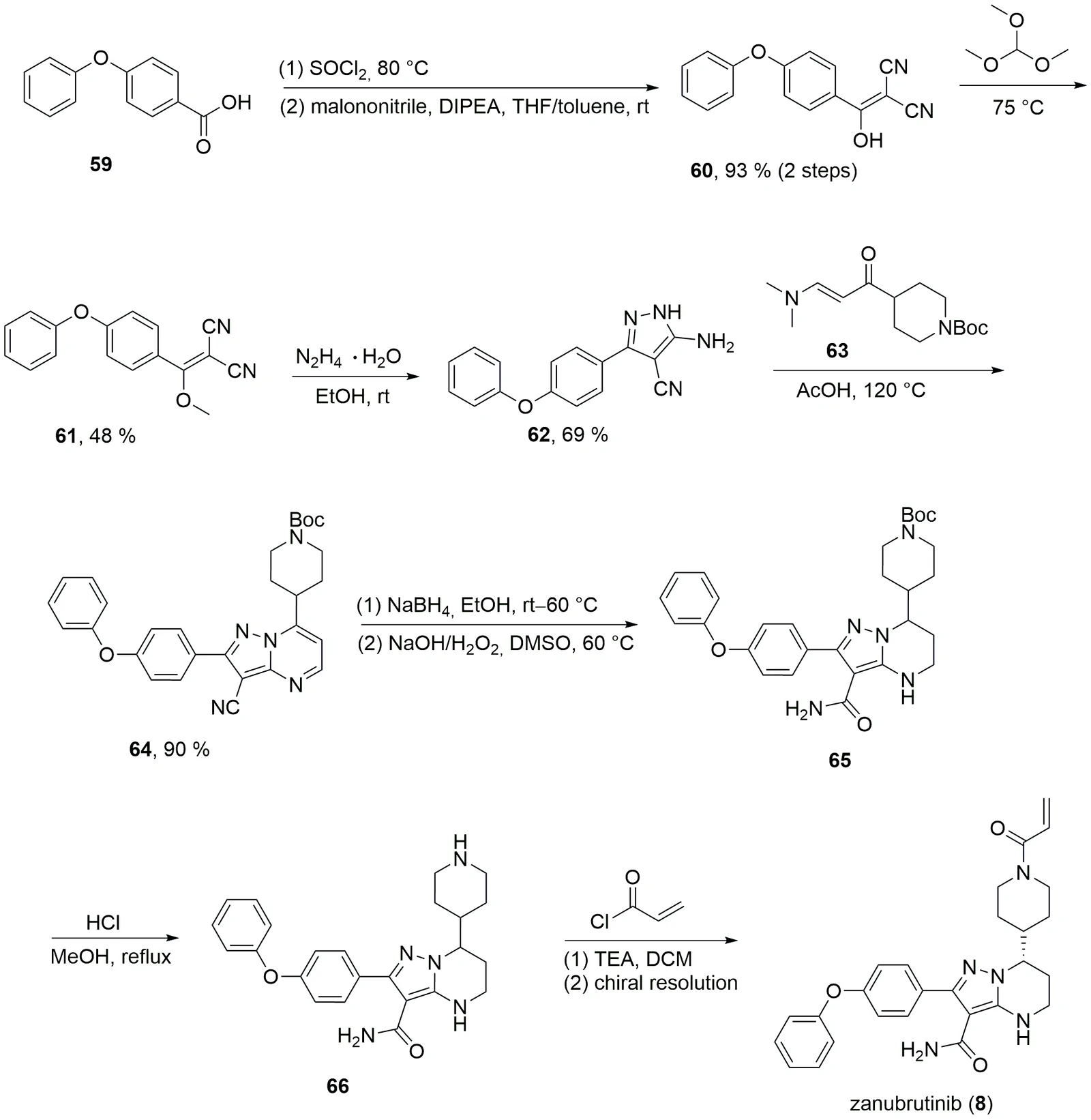
Synthesis of zanubrutinib (**8**).

Its synthesis (Scheme 8) [90] develops from the conversion of 4-phenoxybenzoic acid (**59**) to an acyl chloride intermediate using thionyl chloride, which is subsequently reacted with malononitrile and DIPEA in a THF/toluene mixture at room temperature to yield **60** in 93% yield over two steps. Next, **60** is treated with trimethyl orthoformate to afford the enol ether **61**, which then undergoes cyclization with hydrazine monohydrate in ethanol at room temperature to provide the aminopyrazole derivative **62** (69%). Condensation of **62** with the enaminone **63** in acetic acid constructs the pyrazolo[1,5-a]pyrimidine core structure of **64** (90%). The pyrimidine ring of **64** is then partially reduced using sodium borohydride in ethanol, followed by a localized nitrile hydrolysis using sodium hydroxide and hydrogen peroxide in DMSO to form the carboxamide intermediate **65**. Subsequent global deprotection of the Boc group on **65** is achieved using hydrochloric acid in refluxing methanol to deliver the free piperidine **66**. Finally, **66** is acylated with acryloyl chloride in the presence of triethylamine in dichloromethane and subjected to chiral resolution (column CHIRALPAK 1C) to deliver the active enantiomer, (*S*)-zanubrutinib (**8**). One impurity that could form during its synthesis is the deacylated derivate (loss of the acryloyl group) [91]. Zanubrutinib is a next-generation Bruton tyrosine kinase (BTK) inhibitor that provides effective and sustained disease control in B-cell malignancies, with improved selectivity designed to reduce off-target adverse effects compared with earlier BTK inhibitors [92].

### 2.9. Siponimod (***9***)

**Figure 5:**
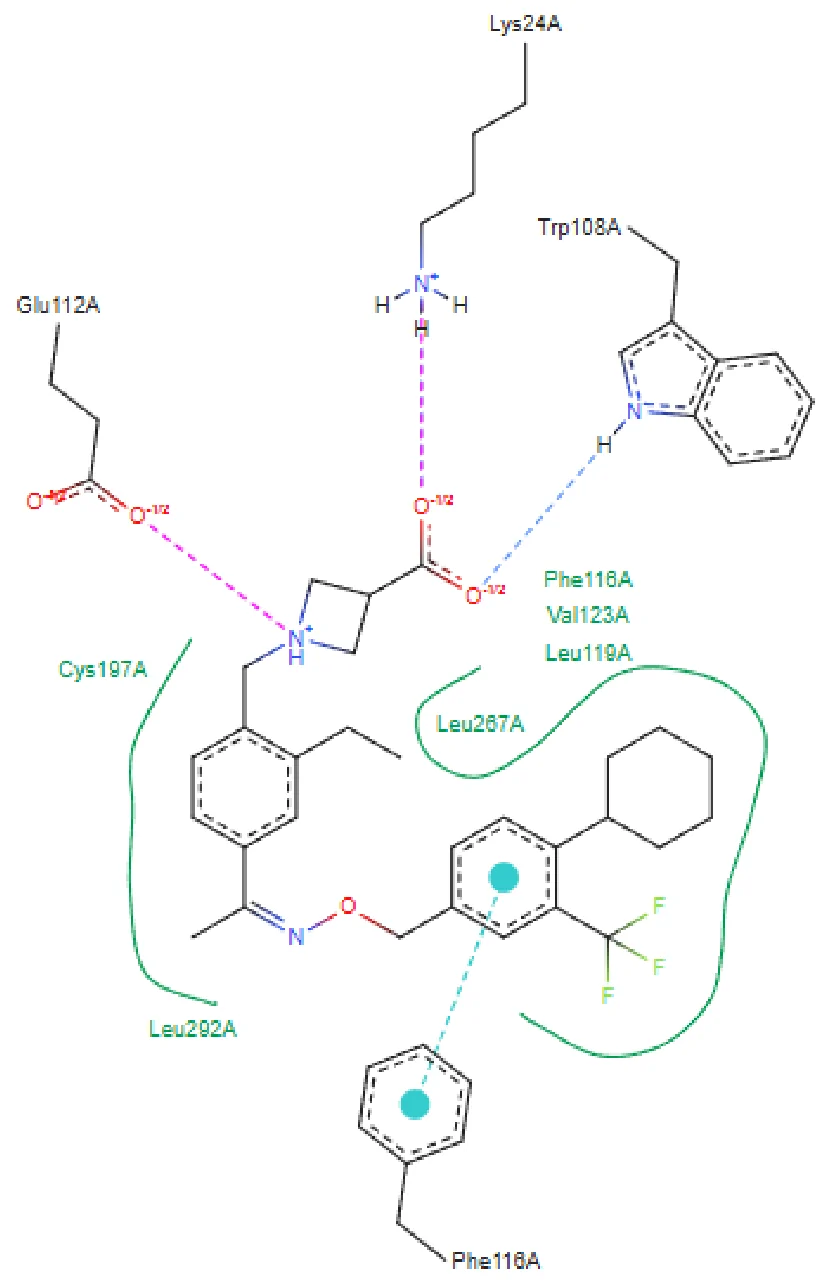
A 2D interaction map (PDB: 7EW1, cryo-EM structure of siponimod-bound Sphingosine-1-phosphate receptor 5 in complex with Gi protein) generated by PoseEdit [14].

Approved on 9 May 2025, siponimod (Mayzent™) is an oral medication used to treat adults with secondary progressive multiple sclerosis, with or without active relapses [93]. It works by selectively binding to sphingosine 1-phosphate receptor subtype 1 (S1P_1_) and S1P_5_ receptors, which helps keep certain immune cells trapped in lymph nodes, preventing them from attacking the central nervous system [94]. This reduces inflammation and slows the progression of disability. Siponimod also has potential direct effects on brain cells that may support neuroprotection [94]. Common side effects include headache, high blood pressure, and increased liver enzymes, while more serious risks may include infections, heart rhythm changes, macular edema, and reduced lung function [95]. Chemically, siponimod contains a benzyloxy oxime moiety and a trifluoromethyl group attached to a substituted benzene ring. Figure 5 shows the binding interactions of the ligand within the protein pocket, highlighting a combination of key hydrogen bonds and hydrophobic contacts. The ligand forms stabilizing hydrogen-bond interactions with Glu112A and Lys24A, while additional polar contacts involving Trp108A help anchor the heterocyclic core and define ligand orientation. The hydrophobic region (outlined in green) accommodates the aromatic and aliphatic substituents of the ligand and is formed by Leu27A, Val123A, Leu119A, Leu292A, and Phe118A, providing extensive van der Waals stabilization. Notably, the proximity of Cys197A suggests potential relevance for selectivity or covalent targeting, depending on ligand reactivity. The trifluoromethyl-substituted aromatic ring is deeply embedded in the nonpolar pocket, indicating its importance for binding affinity and shape complementarity.

The electrostatic potential map (Table 9) shows a predominantly hydrophobic and weakly polarized surface, with localized regions of higher electron density around heteroatom-containing substituents that likely participate in polar receptor interactions. Together, these descriptors support the view that siponimod’s activity arises mainly from hydrophobic and localized electrostatic interactions rather than high frontier-orbital-driven reactivity. Siponimod displays a distinctly lipophilic profile (Table 9), with a low TPSA of 62.13 Å^2^ favoring efficient passive permeability; a high consensus Log P (o/w) of 5.79 that enhances membrane penetration but raises potential concerns around solubility, metabolic clearance, and off-target effects; and a synthetic accessibility score of 4.63 indicative of moderate structural complexity yet acceptable synthetic feasibility.

**Scheme 9:**
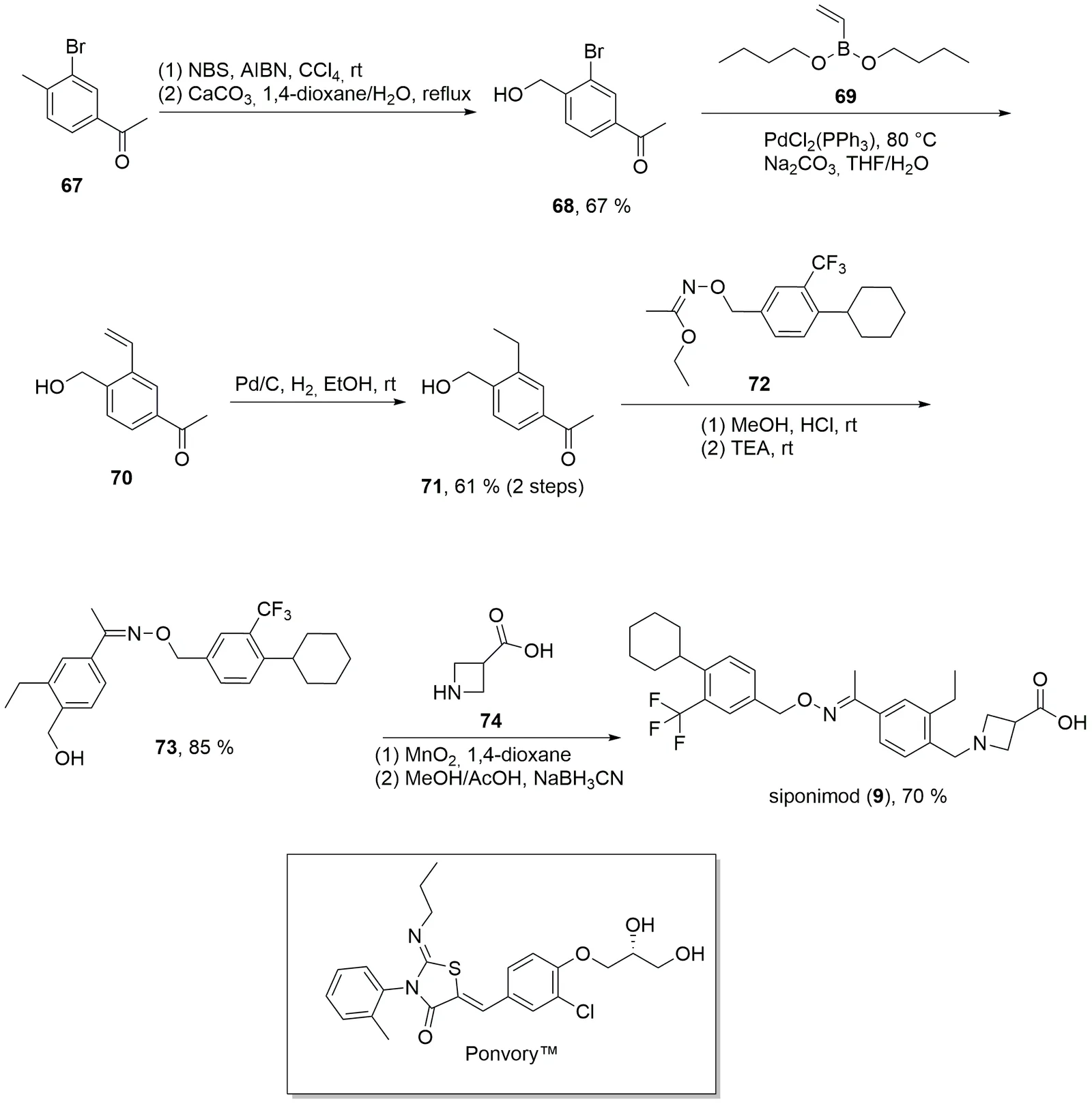
Synthesis of siponimod (**9**). Ponesimod (Ponvory™) shown in the grey box.

Its synthesis [90] (Scheme 9) originates from the radical bromination of **67** using *N*-bromosuccinimide and azobisisobutyronitrile at room temperature, followed by nucleophilic substitution with calcium carbonate in a refluxing dioxane and water mixture to yield the benzyl alcohol **68** (67%). This intermediate then undergoes a Suzuki–Miyaura coupling with the vinylboronate **69** catalyzed by bis(triphenylphosphine)palladium(II) dichloride in a mixture of tetrahydrofuran, water, and sodium carbonate to form **70**. Hydrogenation of the vinyl group over palladium on carbon in ethanol at room temperature yields the ethyl derivative **71** (61% over two steps). Next, **71** is coupled with the oxime ether **72** via a sequence involving hydrochloric acid in methanol followed by triethylamine at room temperature to form the oxime **73** (85%). The primary alcohol of **73** is then oxidized using manganese dioxide in dioxane, and the resulting aldehyde undergoes reductive amination with the azetidine derivative **74** using sodium cyanoborohydride in a mixture of methanol and acetic acid to provide siponimod (**9**, 70%). Related impurities of siponimod fumarate were considered critical quality attributes requiring accurate monitoring during pharmaceutical analysis. A robust HPLC method was developed to achieve effective separation and quantification of SIP and its related impurities, ensuring reliable impurity profiling and quality control of both the bulk drug and tablet dosage forms [96]. Siponimod and ponesimod (Ponvory™, Scheme 9) are selective S1P receptor modulators that effectively reduce disease activity in multiple sclerosis. Siponimod has demonstrated benefits in secondary progressive MS, while ponesimod offers flexible dosing and rapid reversibility due to its shorter half-life [97].

### 2.10. Ivosidenib (***10***)

**Figure 6:**
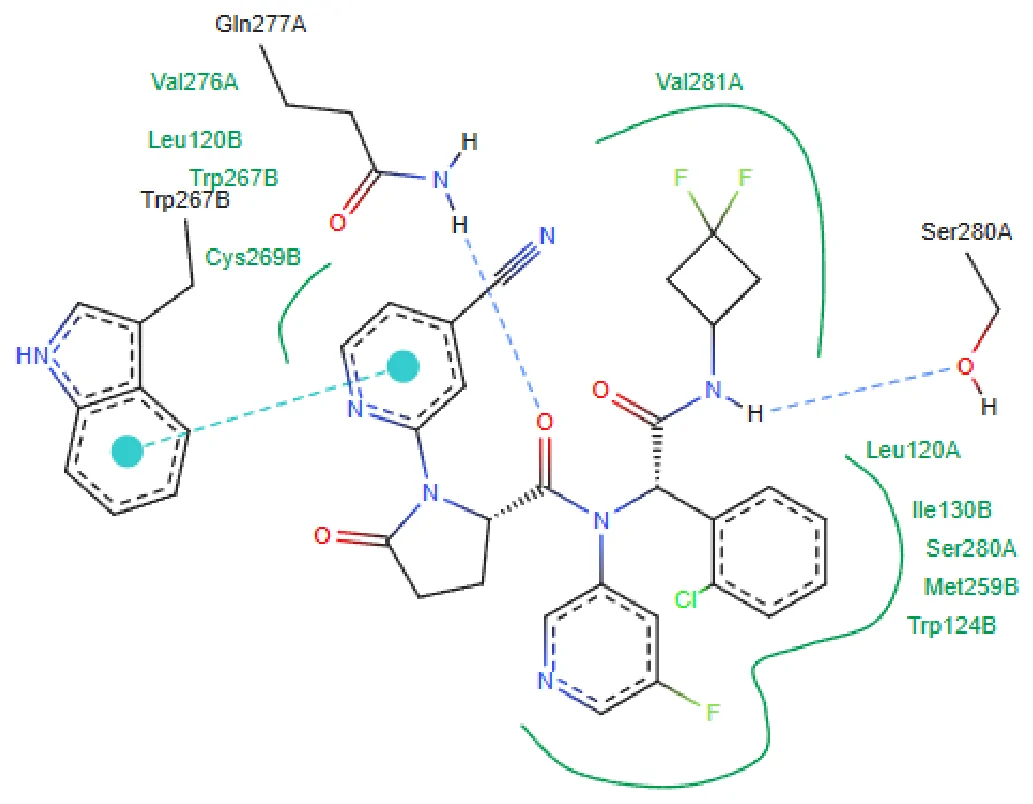
A 2D interaction map (PDB: 8T7O, crystal structure of the R132H mutant of IDH1 bound to ivosidenib) generated by PoseEdit [14].

Approved on 14 May 2025, ivosidenib (Tibsovo™) is an oral anticancer medication that inhibits the mutant isocitrate dehydrogenase-1 (IDH1) enzyme, which is found in certain leukemias and solid tumors [98]. Mutant IDH1 produces an abnormal metabolite called 2-hydroxyglutarate, which disrupts normal cell differentiation [99]. By blocking this enzyme, ivosidenib lowers 2-hydroxyglutarate levels and allows cancer cells to mature and stop proliferating [100]. It is approved for the treatment of acute myeloid leukemia and cholangiocarcinoma with susceptible IDH1 mutations [101]. Common side effects include fatigue, nausea, diarrhea, elevated liver enzymes, and prolongation of the QT interval, and a serious potential complication is differentiation syndrome, which requires prompt recognition and treatment [102]. Chemically, it contains two pyridine rings and a very unusual 3,3-difluorocyclobutyl)amino moiety. Figure 6 depicts the binding pose of the ligand within the active site of the protein, highlighting a well-defined combination of polar interactions and hydrophobic enclosure. The ligand establishes multiple hydrogen bonds, notably with Gln277A and Ser280A, which anchor the central amide and nitrile functionalities and contribute to precise ligand orientation. Additional polar stabilization is provided by interactions involving backbone or side-chain atoms near Trp267B. The hydrophobic pockets (outlined in green) play a major role in ligand stabilization. One region accommodates the aromatic heterocycle through interactions with Val276A, Leu120B, Trp267B, and Cys269B, while a second nonpolar cavity engages the substituted phenyl moiety via Leu120A, Ile130B, Met259B, Trp124B, and Ser280A. The fluorinated substituents enhance lipophilic contacts and shape complementarity, reinforcing binding affinity. The cyclopropyl/fluoroalkyl group projects toward Val281A, further optimizing pocket occupancy.

The electrostatic potential surface shows a predominantly weakly polarized, hydrophobic framework with localized electron-rich regions around heteroatom-containing fragments, which likely serve as hydrogen-bond acceptors and contribute to receptor recognition. Together, the global gap and ESP features suggest that ivosidenib’s activity is governed primarily by hydrophobic and localized polar interactions rather than high frontier-orbital-driven reactivity. Ivosidenib exhibits a polarity–lipophilicity balance characteristic (Table 10) of many targeted oncology agents, with a TPSA of 119.29 Å^2^ that may limit passive permeability, a moderate-to-high consensus Log P (o/w) of 3.40 that helps offset this polarity and support oral exposure, and a synthetic accessibility score of 4.42 reflecting moderate structural complexity but feasible development and manufacture.

**Scheme 10:**
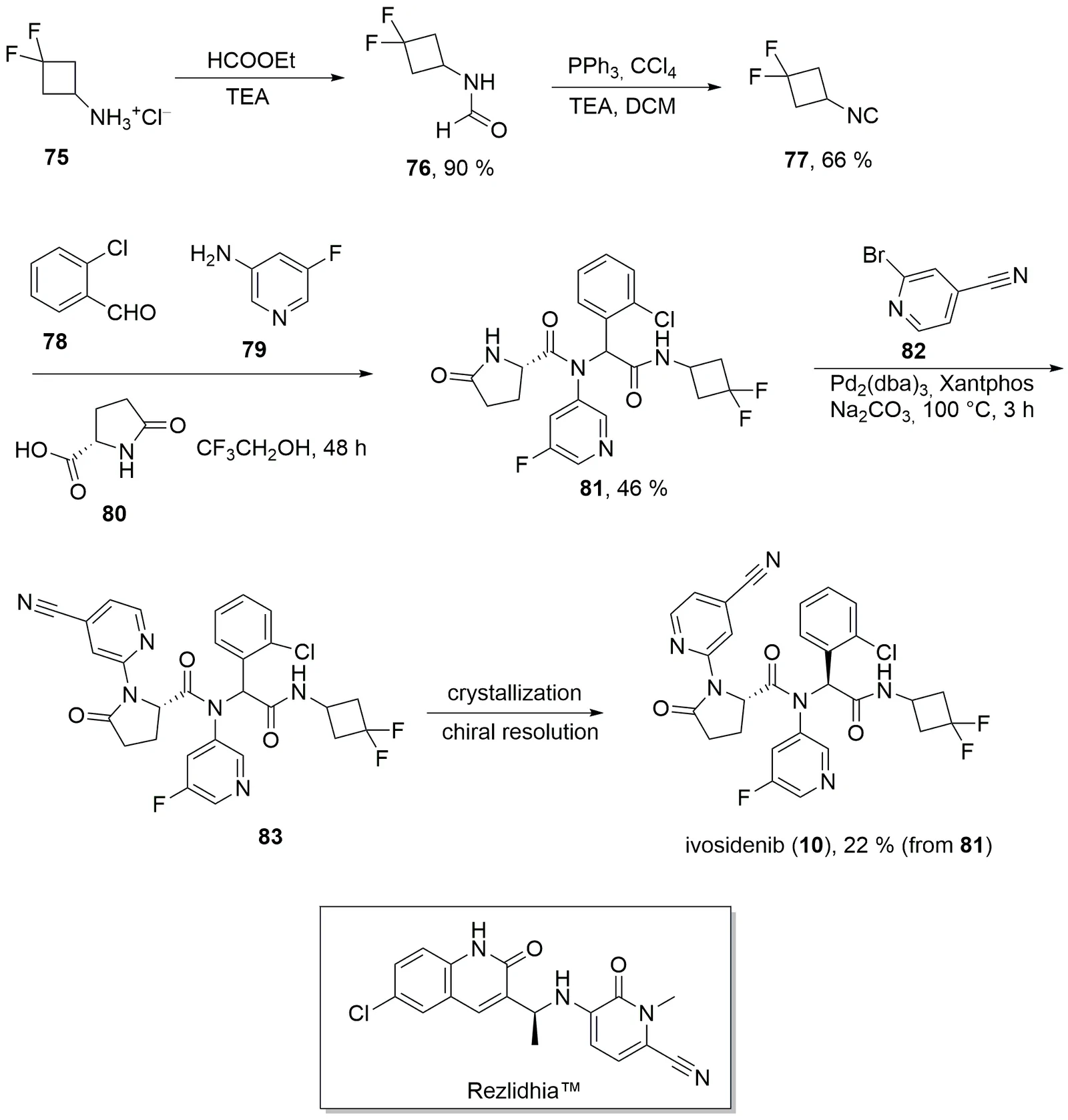
Synthesis of ivosidenib (**10**). Olutasidenib (Rezlidhia™) shown in the grey box.

Its synthesis [103] is shown in Scheme 10. It begins with the formylation of the cyclobutylammonium salt **75** using ethyl formate and triethylamine to give the formamide **76** (90%). This intermediate undergoes dehydration with triphenylphosphine and carbon tetrachloride in the presence of triethylamine and dichloromethane to afford the isocyanide **77** (66%). Next, an Ugi four-component reaction (46%) is performed by assembling the benzaldehyde derivative **78**, the aminopyridine **79**, the pyrrolidone carboxylic acid **80**, and the newly prepared isocyanide **77** in trifluoroethanol to construct the diastereomeric bis-amide structure **81**. Palladium-catalyzed Buchwald–Hartwig amination is then carried out to couple the amide nitrogen of **81** with the bromopyridine **82**, using tris(dibenzylideneacetone)dipalladium(0), Xantphos, and sodium carbonate to yield **83**. Finally, **83** undergoes a crystallization-induced resolution process to isolate the desired pure diastereomeric ivosidenib (**10**) in 22% yield. Three process-related impurities of ivosidenib, namely ethyl ester, isonicotinamide, and *N*-oxide impurities, were investigated and successfully resolved using the developed green HPLC method [104]. Olutasidenib (Rezlidhia™, Scheme 10) and ivosidenib are both IDH1 inhibitors that provide meaningful clinical benefit in relapsed or refractory IDH1-mutated AML. Olutasidenib may offer activity in some patients previously exposed to venetoclax, while ivosidenib has broader clinical experience and established use across multiple IDH1-mutated malignancies.

### 2.11. Tegoprazan (***11***)

Approved on 28 May 2025, tegoprazan (PCAB™) is a potassium-competitive acid blocker (P-CAB) used to treat acid-related disorders such as gastroesophageal reflux disease and peptic ulcers [105]. It works by reversibly and competitively inhibiting the H^+^/K^+^-ATPase proton pump in stomach parietal cells, which leads to rapid and sustained suppression of gastric acid secretion [106]. Unlike proton-pump inhibitors, tegoprazan does not require activation in an acidic environment and provides a faster onset of action [107]. It is generally well-tolerated, with common side effects including headache, diarrhea, and abdominal discomfort [108]. Chemically, it is a benzimidazole that displays a dimethylacetamide and a difluoro-chromane ring. The electrostatic-potential map shows a predominantly weakly polarized, hydrophobic molecular surface with localized electron-rich regions around heteroatom-containing substituents, which are likely involved in specific hydrogen-bonding and polar interactions within the receptor site. These results suggest that tegoprazan’s activity is governed mainly by hydrophobic and localized electrostatic binding interactions rather than intrinsic electronic reactivity. Moreover, the fluorine substituents exert a strong inductive electron-withdrawing effect that locally increases electron density at the C–F region while withdrawing electron density from the adjacent aromatic framework. Tegoprazan shows a favorable balance of physicochemical properties (Table 11), with a TPSA of 67.45 Å^2^ supportive of efficient passive permeability and oral absorption, a moderate consensus Log P (o/w) of 3.44 that enables adequate membrane penetration without excessive lipophilicity, and a synthetic accessibility score of 3.59 indicating good synthetic tractability and developability.

**Scheme 11:**
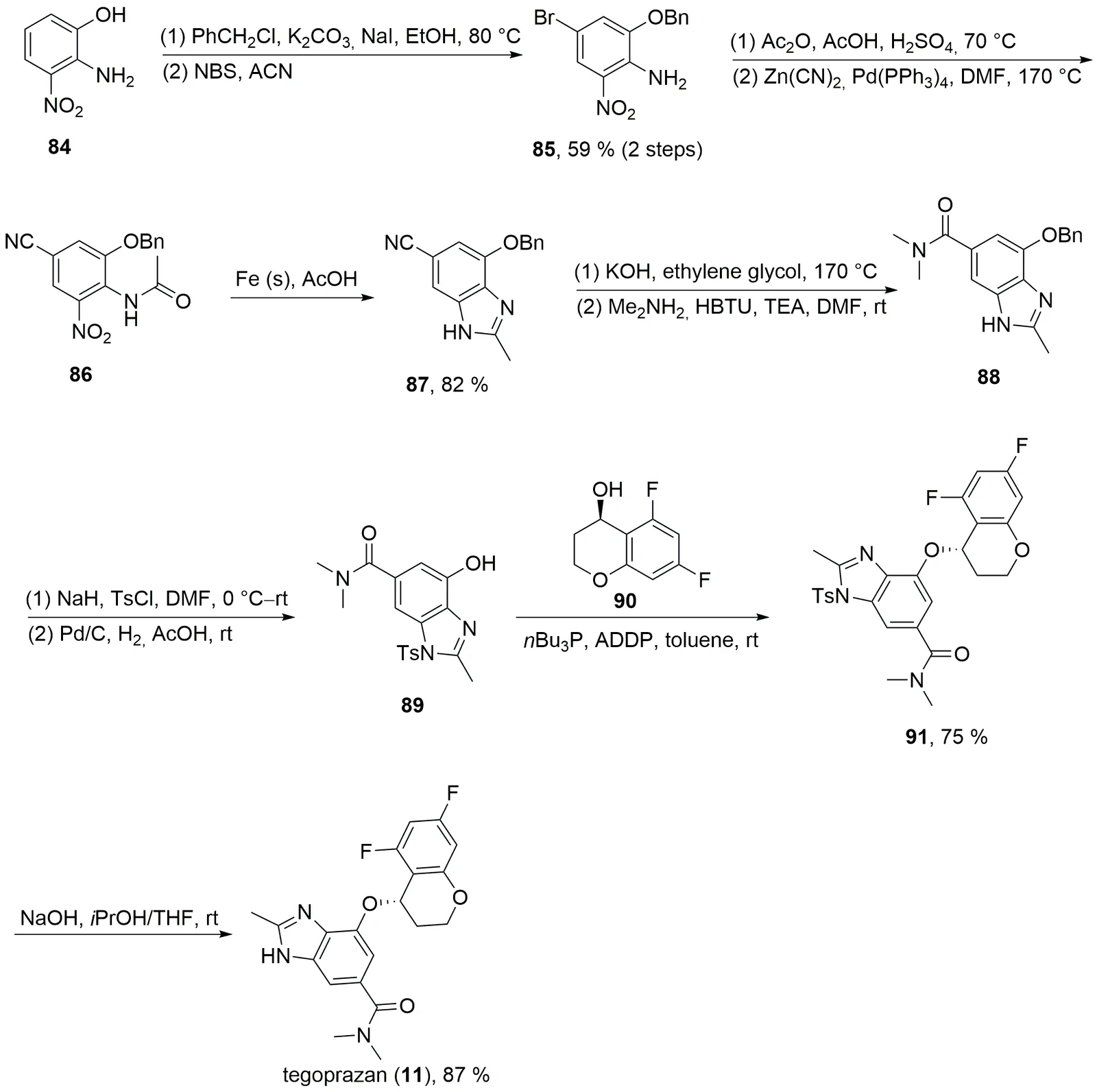
Synthesis of tegoprazan (**11**).

Its synthesis [109] is shown in Scheme 11. It begins from the protection of the phenolic hydroxyl group of **84** as a benzyl ether using benzyl chloride, followed by regioselective aromatic bromination with *N*-bromosuccinimide to afford **85** (59% over two steps). Acetylation of the primary amine in **85**, followed by palladium-catalyzed cyanation, replaces the aryl bromide to yield **86**. Reduction of the nitro group in **86** with iron powder triggers in situ intramolecular cyclization with the acetamido group, constructing the benzimidazole core of **87** (82%). Base-catalyzed hydrolysis of the nitrile group in **87** generates a carboxylic acid intermediate, which is coupled with dimethylamine using HBTU to afford the amide functional group in **88**. Protection of the benzimidazole nitrogen with a tosyl group followed by palladium-catalyzed hydrogenolysis removes the benzyl group to unmask the phenol in **89**. A Mitsunobu coupling of the phenol in **89** with the secondary alcohol of **90** mediated by tributylphosphine and 1,1′-(Azodicarbonyl)dipiperidine (ADDP) proceeds with stereochemical inversion to form the ether linkage in **91** (75%). Finally, base-induced deprotection removes the tosyl protecting group from **91** to deliver the active pharmaceutical ingredient, tegoprazan (**11**, 87%). In a recent study, the major impurity investigated was the enantiomeric impurity of tegoprazan. The undesired (*R*)-enantiomer was considered a chiral impurity and was separated from the active (*S*)-enantiomer using a chiral HPLC method [110]. Tegoprazan and vonoprazan are both effective P-CABs for Helicobacter pylori eradication, with generally comparable efficacy and safety. Vonoprazan has the larger clinical evidence base and established use in eradication regimens, while tegoprazan appears similarly effective and well-tolerated, offering an alternative option where available [111].

### 2.12. Fixed-Dose Combination of Relugolix (***12***), Estradiol (***13***), and Norethindrone (***14***)

A fixed-dose combination of relugolix (**12**), estradiol (**13**), and norethindrone (**14**) (Figure 1) was approved on 16 July 2025 for the management of heavy menstrual bleeding associated with uterine Leiomyomas (fibroids) in premenopausal women [112]. Relugolix (**12**) is an oral gonadotropin-releasing hormone (GnRH) receptor antagonist. It works by directly blocking GnRH receptors in the pituitary gland, which rapidly suppresses the release of luteinizing hormone (LH) and follicle-stimulating hormone (FSH) [113,114]. This leads to a decrease in ovarian or testicular sex hormone production (estrogen and progesterone in women, and testosterone in men) [115]. Estradiol (**13**) is a naturally occurring estrogen hormone and the most potent form of estrogen in humans [116]. It plays a central role in the development and regulation of the female reproductive system, the menstrual cycle, and secondary sexual characteristics [117]. Estradiol also has important effects on bone density, cardiovascular health, and brain function [118]. Norethindrone (**14**) is a synthetic progestin, which is a man-made form of the natural hormone progesterone [119]. It works by binding to progesterone receptors in target tissues, exerting effects similar to natural progesterone [120]. In the uterus, norethindrone stabilizes the endometrial lining, preventing overgrowth that can occur when estrogen acts alone, which reduces the risk of abnormal uterine bleeding [121]. It also thickens cervical mucus, which can inhibit sperm penetration, and alters the endometrium to reduce the likelihood of implantation, which is the basis for its contraceptive effects [122]. The synthetic methodologies for relugolix, estradiol, and norethindrone have been previously reported [97,98,99] and will not be addressed in this work. While a commercial, large-scale synthesis of relugolix has not yet been formally documented, the initial discovery-scale synthetic route developed by researchers at Takeda has been described [123]. The production of estradiol involves a multi-step biosynthetic pathway originating from cholesterol [124]. This process occurs across diverse cellular environments and culminates in the aromatase-catalyzed synthesis of the final estrogen product. The synthesis of norethindrone (17α-ethynyl-17β-hydroxyestr-4-en-3-one) is initiated from the precursor 19-nor-4-androsten-3,17-dione [125].

### 2.13. Stamicis (***15***)

Approved on 23 July 2025, stamicis (**15**) 1 mg is a radiopharmaceutical kit used to prepare a diagnostic imaging agent called Technetium-99m sestamibi [126]. The kit itself contains a chemical that, when combined with a radioactive source in a radiopharmacy, forms the active imaging compound. This compound is injected into the patient and accumulates in certain tissues, allowing doctors to visualize the tissue using a gamma camera [13]. It is also employed for the assessment of global ventricular function, using techniques such as first-pass imaging to determine ejection fraction and ECG-triggered gated SPECT to evaluate left ventricular ejection fraction, volumes, and regional wall motion. Additionally, it can be used in scintimammography to detect suspected breast cancer when conventional mammography results are equivocal, inadequate, or indeterminate. Stamicis is also used for the localization of hyperfunctioning parathyroid tissue in patients with recurrent or persistent disease in both primary and secondary hyperparathyroidism, as well as in patients with primary hyperparathyroidism scheduled for initial parathyroid surgery [13].

### 2.14. Upadacitinib (***16***)

Approved on 18 August 2025, upadacitinib (Rinvoq™) is a prescription JAK inhibitor used to treat several inflammatory conditions such as rheumatoid arthritis, psoriatic arthritis, atopic dermatitis, ulcerative colitis, Crohn’s disease, and ankylosing spondylitis [127]. It works by blocking specific enzymes involved in the immune response, which helps reduce inflammation and ease symptoms like pain, swelling, itching, and intestinal discomfort [128]. Common side effects include headache, nausea, acne, and upper respiratory infections, while more serious but less common risks include infections, blood clots, heart-related events, and certain cancers [127]. Clinicians usually monitor patients regularly to ensure safe use. Chemically, it is an imidazo[1,2-a]pyrrolo[2,3-e]pyrazine derivative. Upadacitinib-related PDB structures could not be retrieved, as no corresponding entries were found in the database. Overall, this value underscores the well-tuned electronic profile of the molecule and supports its potential effectiveness within its therapeutic context. A TPSA of 78.32 Å^2^ suggests the molecule can effectively permeate cell membranes, supporting its role as an orally administered small molecule. A Log P of 2.48 indicates moderate lipophilicity, which is ideal for drug-like molecules. It strikes the right balance by being lipophilic enough to cross lipid bilayers (membranes). This contributes to its high permeability across the pH range of 1–7.5. A synthetic accessibility score of 3.75 suggests that upadacitinib is relatively easy to produce. This low-to-moderate complexity is beneficial for large-scale pharmaceutical manufacturing (Table 12).

**Scheme 12:**
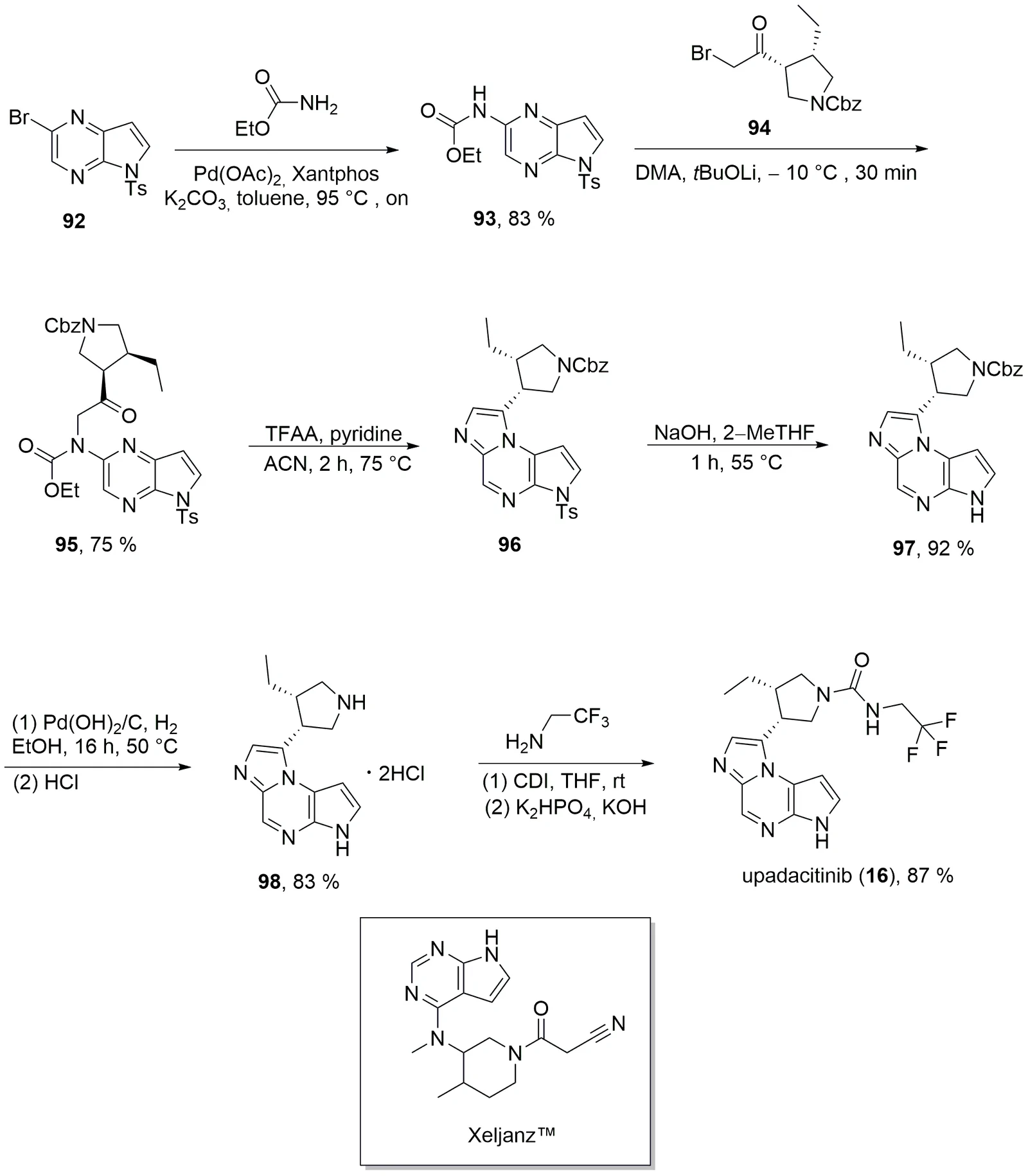
Synthesis of upadacitinib (**16**). Tofacitinib (Xeljanz™) shown in the grey box.

Its synthesis [129] (Scheme 12), chosen due to the high overall yield, begins with palladium-catalyzed Buchwald–Hartwig amidation of the bromopyrrolopyrazine **92** using ethyl carbamate to yield the carbamate intermediate **93** (83%). Alkylation of the carbamate nitrogen in **93** with the bromoketone **94** under basic conditions provides the substituted acyclic adduct **95** (75%). Dehydrative cyclization of **95** mediated by trifluoroacetic anhydride and pyridine promotes intramolecular condensation to construct the tricyclic imidazopyrrolopyrimidine core of **96**. Base-catalyzed hydrolysis of **96** clears the tosyl protecting group from the pyrrole nitrogen to deliver **97** (92%). Subsequent palladium-catalyzed hydrogenolysis removes the carboxybenzyl protecting group from the pyrrolidine ring of **97**, which is then converted to its dihydrochloride salt to afford **98** (83%). Finally, a 1,1′-carbonyldiimidazole (CDI)-mediated urea formation couples the secondary amine of **98** with 2,2,2-trifluoroethylamine, followed by a basic workup to furnish the active pharmaceutical ingredient, upadacitinib, in 87% yield. Forced degradation studies of upadacitinib revealed the formation of twelve novel degradation impurities, predominantly under oxidative and UV-photolytic conditions, highlighting the susceptibility of the molecule to oxidation-induced structural transformations [130]. Upadacitinib and tofacitinib (Xeljanz™, Scheme 12) are effective JAK inhibitors for ulcerative colitis, but upadacitinib generally achieves higher rates of clinical and endoscopic remission, while tofacitinib benefits from longer real-world experience and broader clinical use [131].

### 2.15. Pitolisant (***17***)

Approved on 27 August 2025, pitolisant (Wakix™) is a selective histamine H_3_-receptor antagonist and inverse agonist used primarily to treat excessive daytime sleepiness in narcolepsy [132]. By blocking presynaptic H_3_ receptors, it increases the release of histamine and other neurotransmitters such as acetylcholine, norepinephrine, and dopamine in the brain, which promotes wakefulness [133]. Pitolisant is taken orally, has good absorption, and is metabolized mainly through CYP2D6 and CYP3A4 pathways [134]. It is generally well-tolerated but can cause side effects like insomnia, headache, and anxiety [135]. Chemically, it is a small heterocyclic compound derived from a piperidine core and a substituted chloroarene moiety. Pitolisant-related PDB structures could not be retrieved, as no corresponding entries were found in the database. Due to its high electronegativity and strong inductive (–I) effect, the chlorine atom withdraws electron density from the adjacent aromatic system, contributing to the stabilization of the HOMO level and influencing the overall frontier orbital distribution. This effect is clearly reflected in the molecular electrostatic potential (MEP) surface, where the chloro-containing region exhibits a pronounced negative electrostatic potential (electron-rich zone). Such a feature indicates that the chlorine atom acts as a key site for intermolecular interactions, including halogen bonding and electrostatic interactions with positively charged or electron-deficient regions of biological targets. At the same time, its electron-withdrawing nature enhances charge polarization across the molecule, facilitating intramolecular charge transfer (Table 13). Pitolisant is a molecule optimized for rapid brain entry. Its low TPSA and high LogP (Table 13) allow it to bypass the blood–brain barrier with ease, while its high synthetic accessibility reflects a straightforward chemical architecture (a piperidine-ether derivative) that supports its robust bioavailability.

**Scheme 13:**
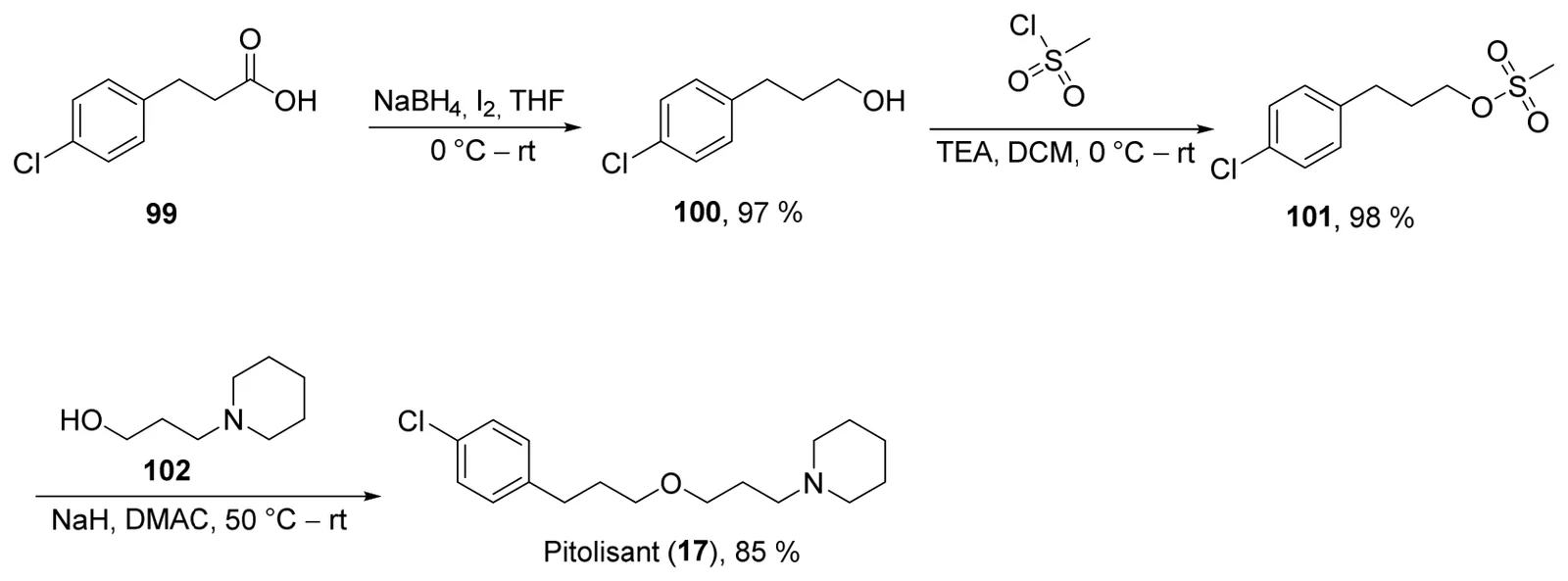
Synthesis of pitolisant (**17**).

While numerous synthetic pathways for pitolisant (**17**) have been published, a large-scale, kilogram-scale synthesis was first reported (Scheme 13) in 2013 [136]. The synthesis of pitolisant begins with the selective reduction of 3-(4-chlorophenyl)propanoic acid, **99**, to its corresponding primary alcohol intermediate, **100**, using sodium borohydride and iodine in tetrahydrofuran, which provides the product in an excellent 97% yield. This intermediate is then activated by treatment with methanesulfonyl chloride and triethylamine in dichloromethane to smoothly afford the mesylate intermediate, **101**, in a nearly quantitative 98% yield. In the final step, Williamson ether synthesis is employed to couple this activated intermediate with 3-(piperidin-1-yl)propan-1-ol, designated as **102**, in the presence of sodium hydride and dimethylacetamide, successfully delivering pitolisant (85%). Overall, the synthetic scheme and total yield (80%) show an easy approach supported by synthetic accessibility of 2.21. Among the identified impurities, pitolisant *N*-oxide was found to be the major oxidative degradation impurity, whereas the drug exhibited good stability under acidic, alkaline, thermal, and photolytic stress conditions [137]. For patients who experience both daytime sleepiness and muscle weakness (cataplexy), the primary brand-name alternatives of pitolisant are the oxybate salts [138].

### 2.16. Etrasimod (***18***)

Approved on 16 September 2025, etrasimod (Velsipity™) is an oral selective modulator of the sphingosine-1-phosphate receptor (S1P), mainly targeting S1P_1_ with additional activity at S1P_4_ and S1P_5_ [139]. By modulating these receptors, it causes controlled sequestration of lymphocytes in lymphoid tissues, reducing the number of circulating immune cells that can drive inflammation [140]. It targets older people with moderately to severely active ulcerative colitis who have had an inadequate response, lost response, or were intolerant to either conventional therapy or a biological agent. Etrasimod is designed to offer more receptor selectivity and a cleaner safety profile than earlier S1P modulators, with a relatively short half-life that allows quicker reversibility of immune effects when therapy is stopped [141]. It is taken once daily and is generally well-tolerated, with common effects including headache, nasopharyngitis, and occasional heart-rate changes when first dosed [142]. Chemically, it is a heterocycle-rich molecule built around an indole core that is substituted with multiple functional groups. Carboxylic acid and trifluoromethyl groups are among these. Etrasimod-related PDB structures could not be retrieved, as no corresponding entries were found in the database. Overall, the isosurface (electron density) map confirms that etrasimod has a well-distributed electrostatic profile suitable for specific receptor binding, consistent with its potency as a selective S1P modulator (Table 14). In particular, the trifluoromethyl group and the carboxylic acid pendant may be important for establishing an interaction with the receptor. Etrasimod is a highly lipophilic (Table 14) small molecule optimized for potency and receptor selectivity. Its profile allows for extensive volume of distribution, facilitating the systemic sequestration of lymphocytes needed to treat ulcerative colitis.

**Scheme 14:**
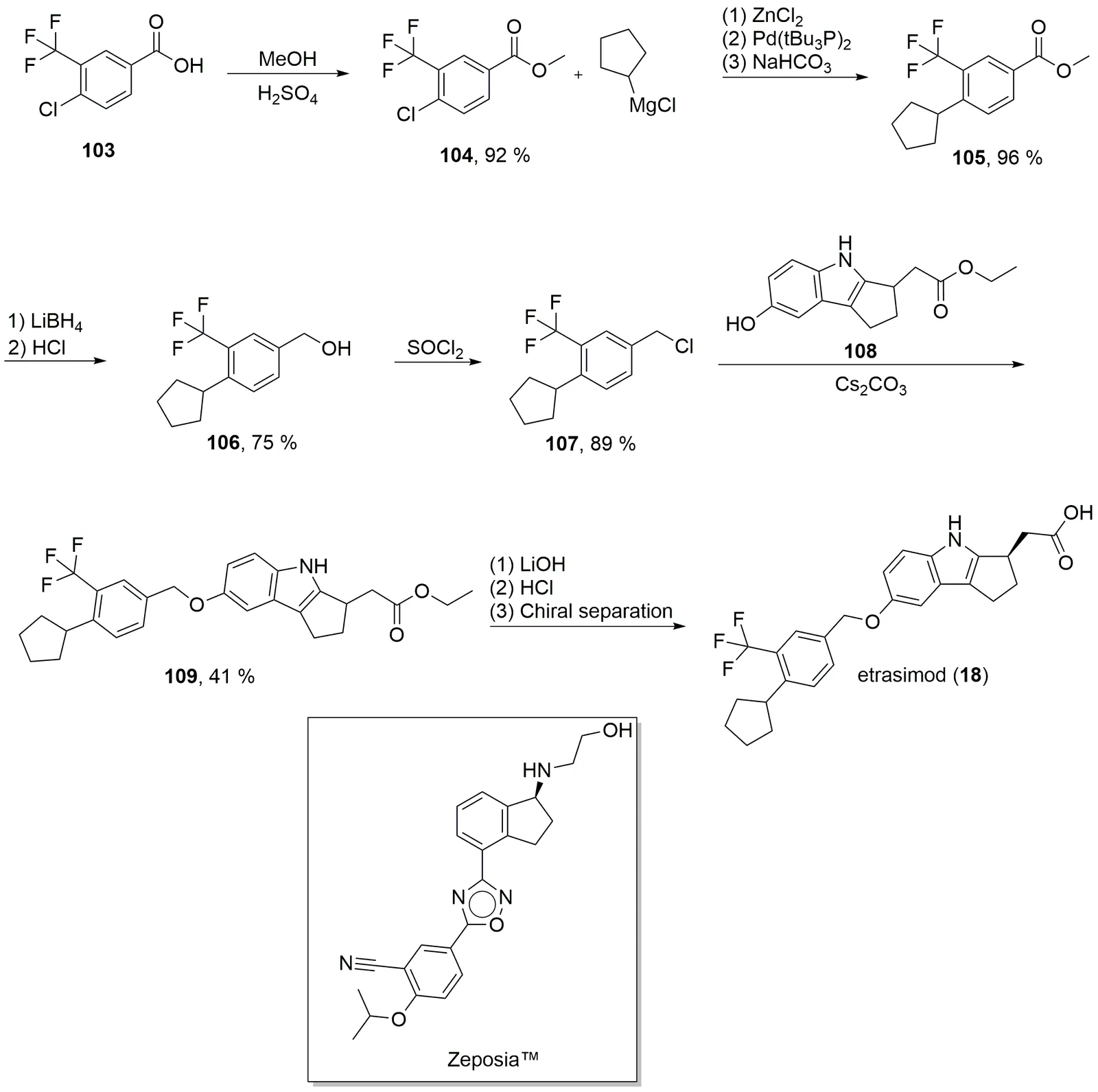
Synthesis of etrasimod (**18**). Ozanimod (Zeposia™) shown in the grey box.

Its synthesis (Scheme 14) [143] develops from 4-chloro-3-(trifluoromethyl)benzoic acid (**103**) to form methyl ester **104** via Fischer esterification (92%), followed by a zinc-mediated Negishi cross-coupling to introduce the cyclopentyl ring. The resulting ester **105** (96%) is reduced using lithium borohydride to give benzyl alcohol **106** (75%), which is subsequently chlorinated with thionyl chloride to afford benzyl chloride **107** (89%). A cesium carbonate-mediated nucleophilic substitution couples **107** with phenol **108** to yield ether **109** (41%), which undergoes final ester hydrolysis and chiral separation (normal phase preparative chiral chromatography) to deliver the active pharmaceutical ingredient, etrasimod (**18**). Acidic, oxidative, and photolytic stress conditions produced degradation impurities, whereas etrasimod showed good stability under basic hydrolysis and elevated temperature conditions [144]. Ozanimod (Zeposia™, Scheme 14) and etrasimod are effective S1P receptor modulators for moderate-to-severe ulcerative colitis, but etrasimod may offer modestly greater efficacy for key clinical and endoscopic outcomes, while ozanimod has longer clinical experience and established use [145].

### 2.17. Erdafitinib (***19***)

**Figure 7:**
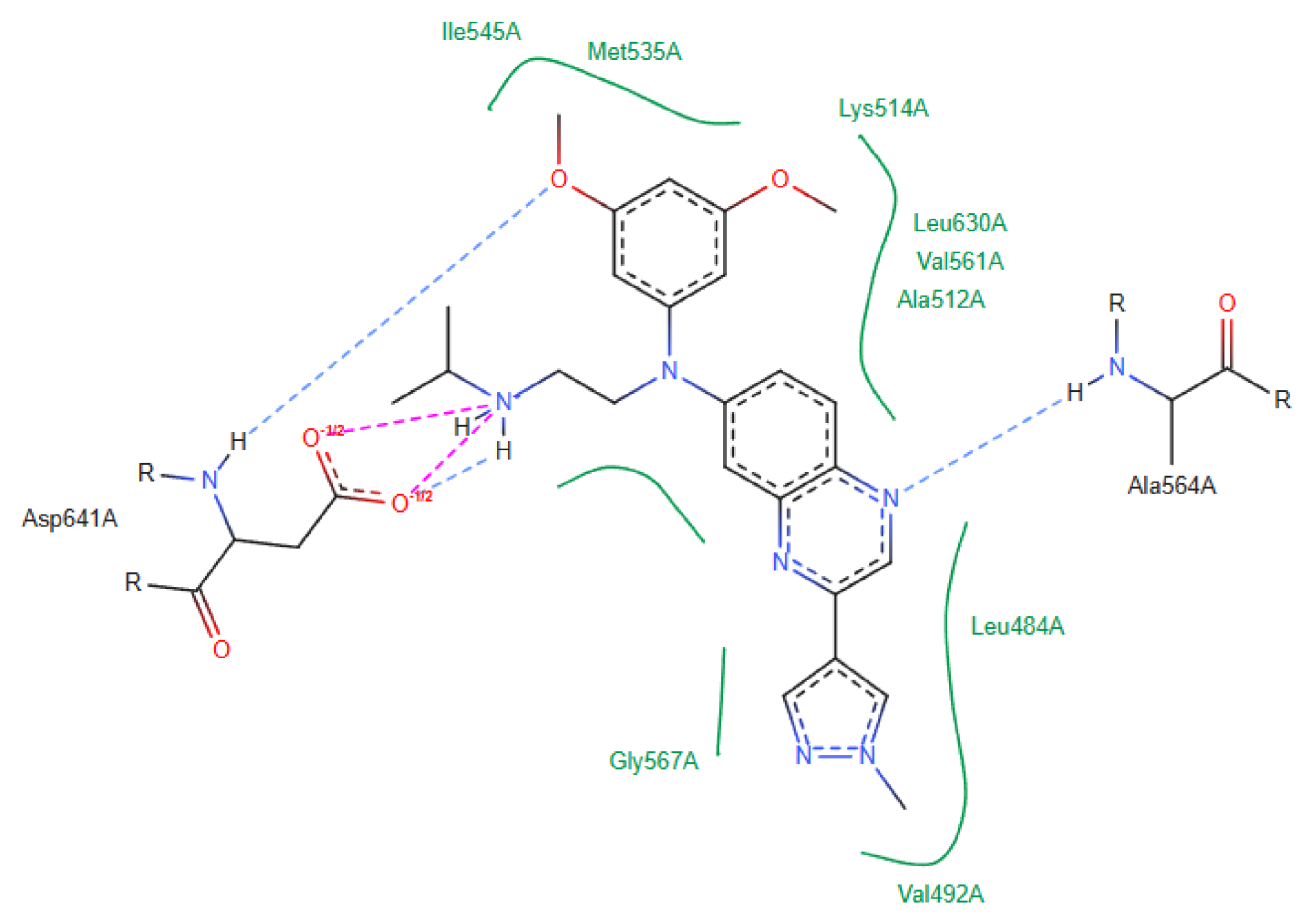
A 2D interaction map (PDB: 5EW8, fibroblast growth factor receptor 1 in complex with erdafitinib) generated by PoseEdit [14].

Approved on 1 October 2025, erdafitinib (Balversa™) is an oral small-molecule inhibitor of fibroblast growth factor receptors (FGFRs) 1–4, used primarily to treat metastatic or locally advanced urothelial carcinoma with susceptible FGFR genetic alterations [146]. By blocking aberrant FGFR signaling, it reduces tumor cell proliferation and survival [147]. It has a manageable safety profile, with common effects like hyperphosphatemia, stomatitis, and hand–foot syndrome, and requires monitoring of serum phosphate levels to guide dosing. Chemically, it is a multifunctional heteroaromatic compound built around a quinoxaline core. Figure 7 depicts the binding pose of the ligand within the active site. Toward the catalytic loop, the terminal heterocycle engages Asp641A through polar and possibly bidentate hydrogen-bonding contacts, providing an additional stabilizing interaction with the conserved acidic residue typical of kinase active sites. Along the length of the scaffold, the aromatic rings and aliphatic substituents are closely packed against a series of hydrophobic residues such as Ile545A, Met535A, Leu484A, Val492A, and Gly567A, indicating extensive van der Waals complementarity that reinforces binding orientation and contributes to affinity. The extended aromatic system appears to project into a deeper hydrophobic back pocket, consistent with inhibitors designed to exploit this subcavity for potency and selectivity, while a weaker peripheral polar interaction near Lys514A likely assists in orienting the ligand without dominating the binding energetics. Overall, the complex is characterized by strong hinge hydrogen bonding, cooperative interactions with Asp641A, and broad hydrophobic burial across the ATP pocket, yielding a binding mode typical of a well-optimized FGFR1 inhibitor.

Overall, the isosurface (electron density) map confirms the interactions shown in Figure 7. The quinoxaline nitrogens both act as hydrogen-bond acceptors. Erdafitinib’s profile is highly drug-like, fitting perfectly within Lipinski’s criteria. Its balanced lipophilicity and TPSA (Table 15) facilitate the high volume of distribution needed to target FGFR-mutated tumors throughout the body.

**Scheme 15:**
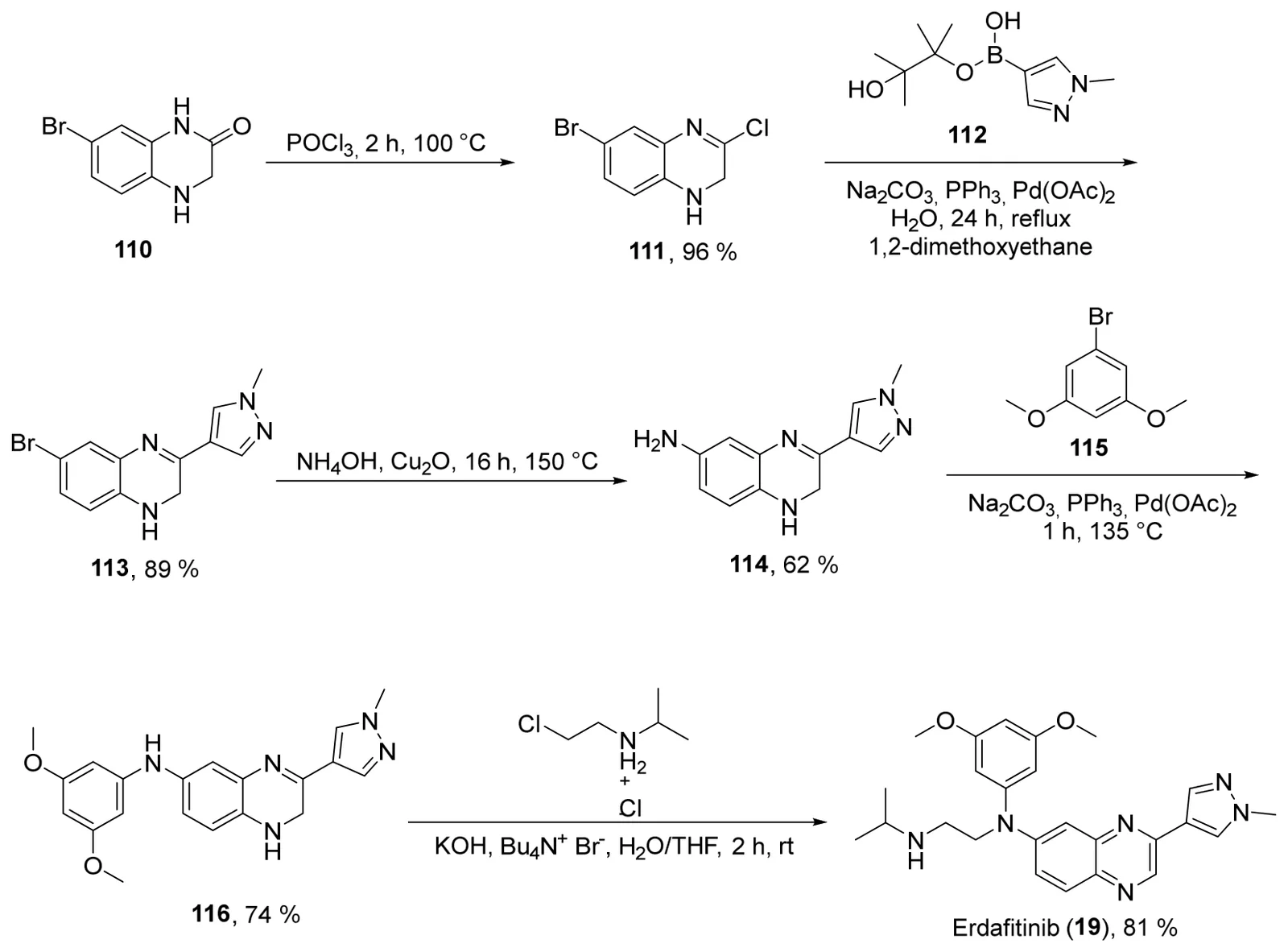
Synthesis of erdafitinib (**19**).

Its synthesis [148] (Scheme 15) develops from the chlorination of quinoxaline **110** using POCl_3_ to yield **111** in 96% yield. Selective Suzuki–Miyaura cross-coupling of **111** with the pyrazole boronic acid derivative **112**, catalyzed by Pd(OAc)_2_ and PPh_3_, affords **113** in 89% yield. Next, a copper-catalyzed amination of the aromatic bromide in **113** using NH_4_OH and Cu_2_O produces the primary amine **114** in 62% yield. Palladium-catalyzed Buchwald–Hartwig amination couples **114** with 1-bromo-3,5-dimethoxybenzene **115** to provide the secondary diarylamine **116** in 74% yield. Finally, phase-transfer alkylation of the secondary aniline in **116** with 2-chloro-*N*-isopropylethan-1-amine hydrochloride using KOH and Bu_4_N^+^ Br^−^ successfully completes the total synthesis of erdafitinib (**19**) in 81% yield. The overall yield was 32%. A total of seven related substances, comprising six degradation products and one impurity, were successfully separated and structurally characterized using HPLC and ion-trap MS techniques [149]. The main competitor drug to erdafitinib is the antibody-drug conjugate enfortumab vedotin (Padcev™) [150].

### 2.18. Vorasidenib (***20***)

**Figure 8:**
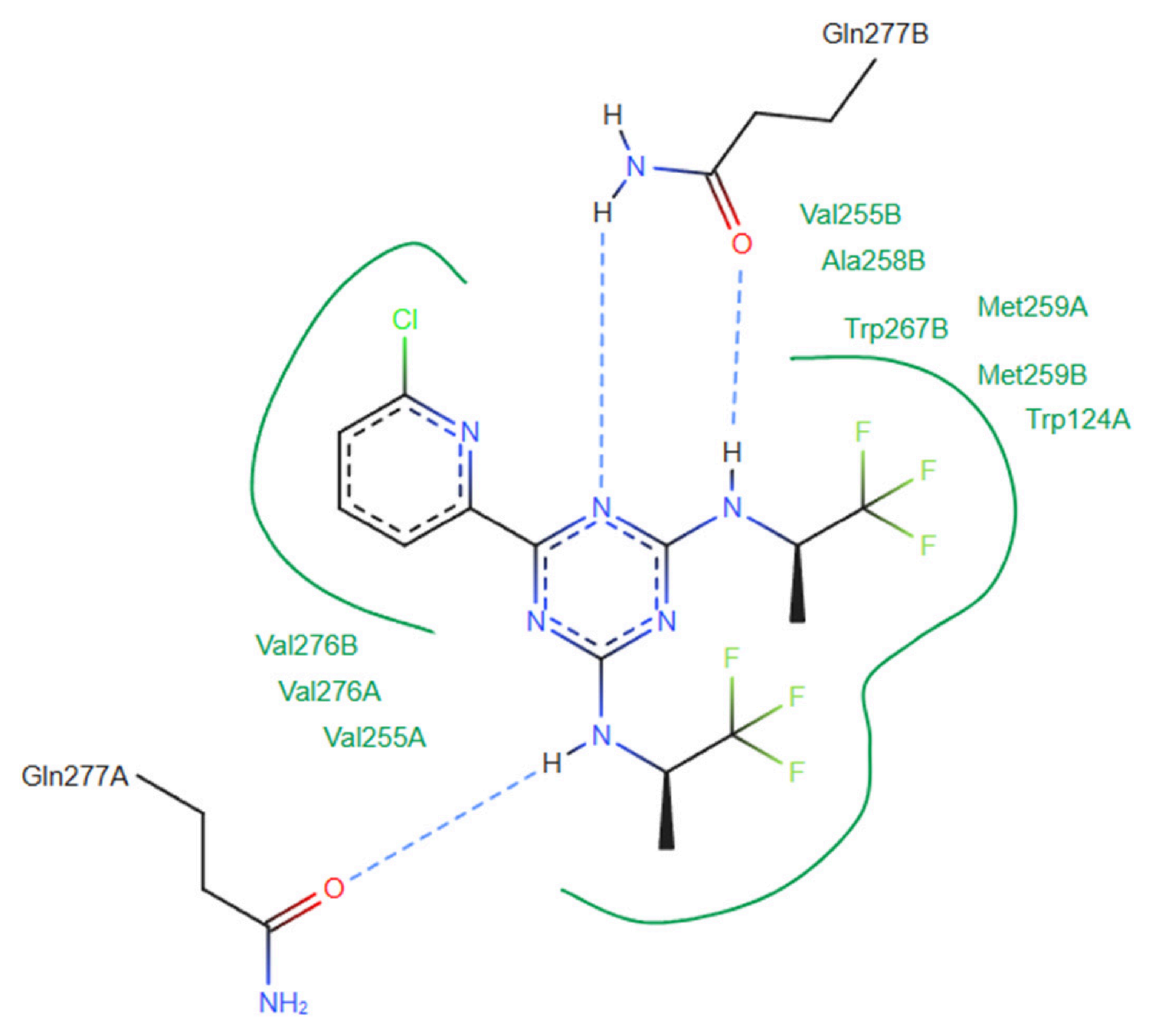
A 2D interaction map (PDB: 6VEI, crystal structure of human cytosolic isocitrate dehydrogenase (IDH1) R132H mutant in complex with NADPH and vorasidenib).

Approved on 15 October 2025, vorasidenib (Voranigo™) has an indication for astrocytoma or oligodendroglioma with a susceptible isocitrate dehydrogenase-1 (IDH1) or isocitrate dehydrogenase-2 (IDH2) mutation after surgery, such as gross complete resection, subtotal resection, or biopsy [151]. A key feature of the drug is its ability to cross the blood–brain barrier, allowing it to act directly within the brain [152]. The medication is taken once daily by mouth and is generally well-tolerated, though common side effects include fatigue, headache, nausea, diarrhea, seizures, and elevations in liver enzymes, which require monitoring [153]. Chemically, it is a highly halogenated triazine molecule (two trifluoromethyl groups and an aromatic chlorine). Figure 8 depicts the binding pose of the ligand within the protein active site. The carbonyl-containing moiety toward Val255B and Ala258B participates in additional polar contacts that further stabilize the complex and reinforce hinge association. Surrounding the aromatic and trifluoromethyl-substituted portions of the ligand is a compact hydrophobic environment formed by residues such as Val255A/B, Val276A/B, Met259A/B, Trp124A, and Trp267B, indicating extensive van der Waals complementarity and tight packing along the ligand periphery. The presence of the trifluoromethyl group promotes deeper insertion into this hydrophobic cleft, enhancing hydrophobic burial and likely contributing to binding affinity. At the opposite end of the molecule, the interaction with Gln277A appears to represent a weaker or more solvent-exposed polar contact that may assist in orienting the terminal substituent without dominating the overall binding energetics. Taken together, the binding mode reflects a balance of strong hinge-directed hydrogen bonding, cooperative peripheral polar contacts, and substantial hydrophobic engagement along the pocket, consistent with a ligand designed to achieve stable and selective recognition within this protein active site.

The isosurface (electron density) map shows that halogens (fluorine and chlorine atoms) play a pivotal role in the binding of this molecule. Vorasidenib’s profile is specifically “tuned” (Table 16) for neuro-oncology. It leverages its LogP of 4.11 to ensure it does not just circulate systemically but achieves the high brain-to-plasma ratio necessary to treat low-grade gliomas effectively.

**Scheme 16:**
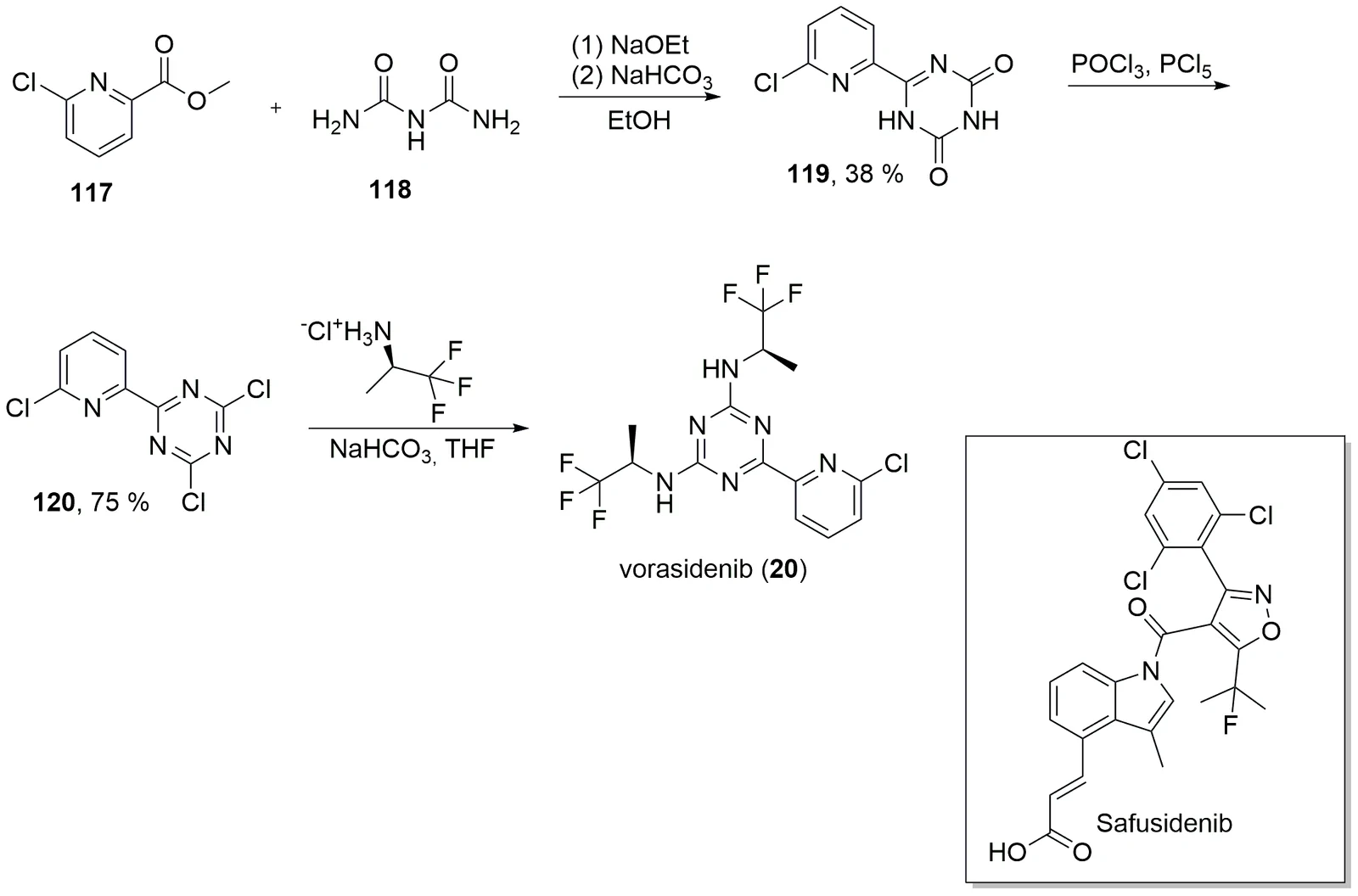
Synthesis of vorasidenib (**20**). Safusidenib, an investigational new drug, shown in the grey box.

Its synthesis (Scheme 16) [152] begins with condensation of **117** and imidodicarbonic diamide **118** in ethanol to yield the triazine-dione **119** (38%). Subsequent chlorination of **119** using phosphorus oxychloride and phosphorus pentachloride affords a dichlorotriazine intermediate **120** (75%), which undergoes a dual nucleophilic aromatic substitution with a chiral trifluoromethylated amine hydrochloride to provide the final drug candidate vorasidenib (**20**). Four process-related impurities associated with vorasidenib were included in an analytical method development, and their complete chromatographic separation was achieved using a Quality by Design (QbD) approach [154]. Vorasidenib and safusidenib are both brain-penetrant IDH inhibitors for IDH-mutant gliomas, but vorasidenib has demonstrated phase III evidence with improved progression-free survival and delayed need for further intervention, whereas safusidenib has shown promising clinical activity and good brain penetration but remains at an earlier stage of clinical development [155].

## 3. Discussion and Trends

Table 17 provides a summary of the approved small molecules by the CDSCO in 2025 according to their therapeutic use and their mechanism of action. Many drugs approved in India in 2025 were already recognized in other major markets (e.g., FDA), indicating regulatory harmonization and increased access to globally validated therapies. Tafamidis (**1**) was approved by the FDA in May 2019 in two formulations: Vyndaqel™ (tafamidis meglumine) and Vyndamax™ (tafamidis) [156]. Letermovir (**2**) was approved by the U.S. Food and Drug Administration (FDA) on 8 November 2017 for the prophylaxis of cytomegalovirus (CMV) infection and disease in adult CMV-seropositive recipients of an allogeneic hematopoietic stem cell transplant (HSCT) [157]. Fexuprazan (**3**) has not been approved by the FDA for use in the United States as of now. Clinical development and regulatory filings for U.S. approval have been in progress, but no official FDA approval date has been announced. Edoxaban (**4**) was approved by the FDA on 8 January 2015 (marketed under the brand name Savaysa™) for the reduction in the risk of stroke and systemic embolism in patients with non-valvular atrial fibrillation, and for the treatment of deep vein thrombosis and pulmonary embolism following initial therapy with a parenteral anticoagulant [158]. Rimegepant (**5**) (marketed as Nurtec ODT™) was first approved by the FDA on 27 February 2020 for the acute treatment of migraine with or without aura in adults [159]. Doravirine (**6**) was approved by the FDA on 30 August 2018 for the treatment of HIV-1 infection in adults as a non-nucleoside reverse transcriptase inhibitor (NNRTI) (brand name Pifeltro™), including use in combination with other antiretroviral agents. Tucatinib (**7**, brand name Tukysa™) was first approved by the FDA on 17 April 2020. It received approval in combination with trastuzumab and capecitabine for the treatment of adult patients with advanced unresectable or metastatic HER2-positive breast cancer, including those with brain metastases, who have received one or more prior anti-HER2-based regimens in the metastatic setting [82]. Zanubrutinib (**8**, brand name Brukinsa™) was first approved by the FDA on 14 November 2019, under accelerated approval for the treatment of adult patients with mantle cell lymphoma (MCL) who have received at least one prior therapy [86]. Siponimod (**9**, brand name Mayzent™) was approved by the FDA on 26 March 2019 for the treatment of relapsing forms of multiple sclerosis (MS), including clinically isolated syndrome, relapsing-remitting disease, and active secondary progressive disease in adults [95]. Ivosidenib (**10**, brand name Tibsovo™) was first approved by the FDA on 20 July 2018 for the treatment of adult patients with relapsed or refractory acute myeloid leukemia (AML) with a susceptible IDH1 mutation [101]. Tegoprazan’s (**11**) FDA review is actively underway, but there has been no official FDA approval yet. The FDA approved the combination of relugolix (**12**), estradiol (**13**), and norethindrone acetate (**14**, Myfembree™) on 8 May 2020 for the management of heavy menstrual bleeding associated with uterine fibroids [112]. Stamicis (**15**) is not an FDA-approved therapeutic drug, but it is a diagnostic radiopharmaceutical product used in medical imaging in some countries (e.g., India). Upadacitinib (**16**, Rinvoq™) was first approved by the FDA on 16 August 2019 for the treatment of moderately to severely active rheumatoid arthritis in adults who did not respond adequately to or are intolerant of one or more TNF blockers [128]. In 2019, the FDA first approved pitolisant (**17**, Wakix™) for the treatment of excessive daytime sleepiness (EDS) in adult patients with narcolepsy [133]. Etrasimod (**18**), marketed under the brand name Velsipity™, was approved by the FDA on 12 October 2023. It is approved for the treatment of moderately to severely active ulcerative colitis in adults [141]. In 2019, the FDA granted accelerated approval to erdafitinib (**19**, Balversa™) for the treatment of locally advanced or metastatic urothelial carcinoma (bladder cancer) with susceptible FGFR3 or FGFR2 genetic alterations after progression on platinum-based chemotherapy [146]. The FDA approved vorasidenib (**20**) on 6 August 2024 for the treatment of adult and pediatric patients (12 years and older) with grade 2 astrocytoma or oligodendroglioma that have a susceptible IDH1 or IDH2 mutation following surgery (including biopsy, sub-total resection, or gross total resection) [151]. The sales for the year 2025 together with other valuable information (commercially stable forms, patent assignees and competitor drugs) have been reported in Table S1 and Figure S1 (Supporting Information).

**Figure 9:**
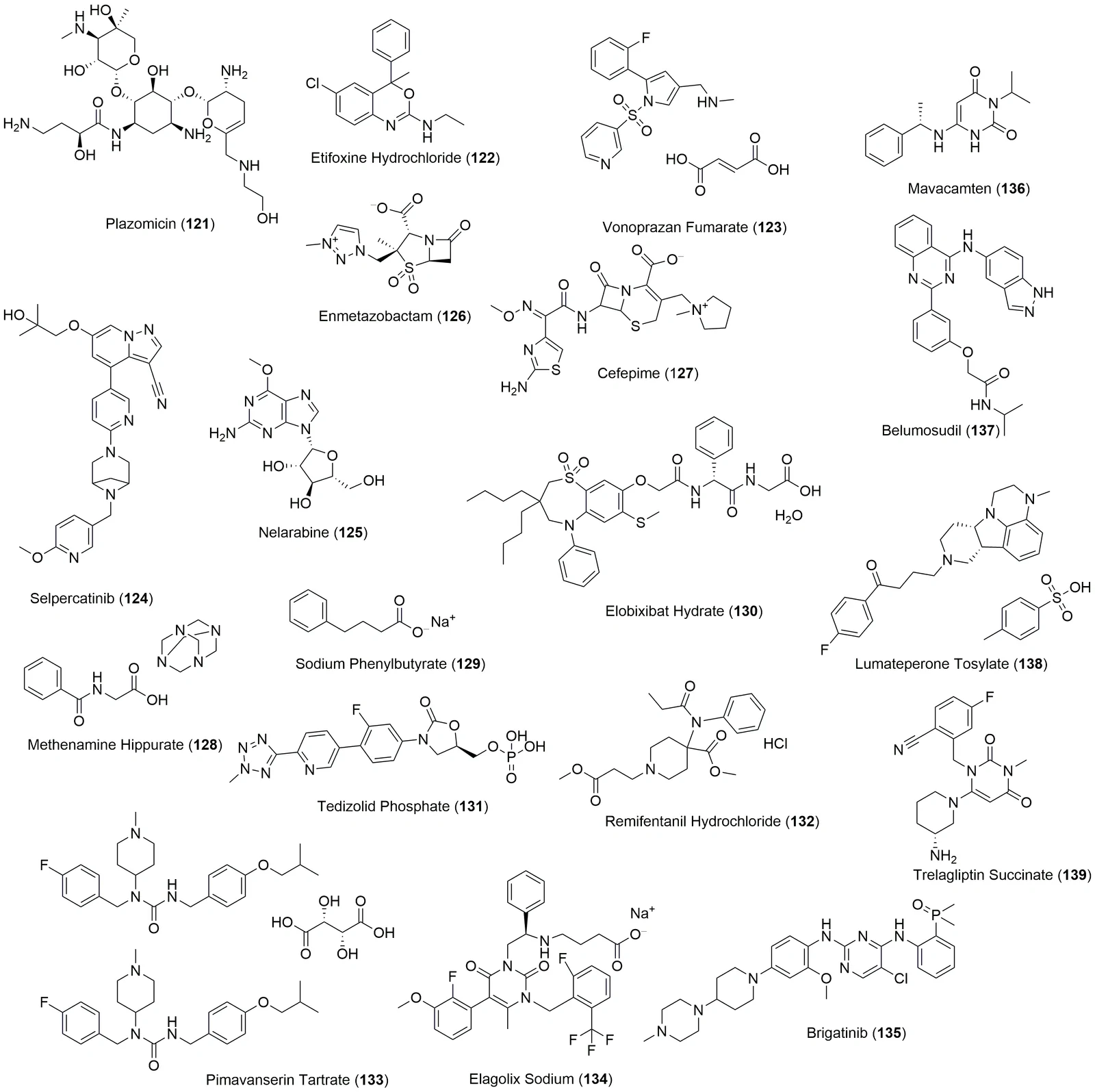
Chemical structures of small molecules approved by CDSCO in 2024.

In the previous year (2024), 19 small molecules were approved [13] (Figure 9). Among these, plazomicin (**121**), etifoxine hydrochloride (**122**), vonoprazan fumarate (**123**), selpercatinib (**124**), nelarabine (**125**), enmetazobactam (**126**) + cefepime (**127**), methenamine hippurate (**128**), sodium phenylbutyrate (**129**), elobixibat hydrate (**130**), tedizolid phosphate (**131**), remifentanil hydrochloride (**132**), pimavanserin tartrate (**133**), elagolix sodium (**134**), brigatinib (**135**), mavacamten (**136**), belumosudil (**137**), lumateperone tosylate (**138**), and trelagliptin succinate (**139**). Table 18 reports a summary of the approved small molecules by the CDSCO in 2024 according to their therapeutic use and their mechanism of action. It is evident that the portfolio appears diversified across three major therapeutic areas (oncology, infectious diseases, and neurology) rather than being dominated by a single therapeutic focus. If grouped broadly, however, oncology-related agents (including hemato-oncology) remain one of the leading clusters, consistent with common industry R&D trends.

**Figure 10:**
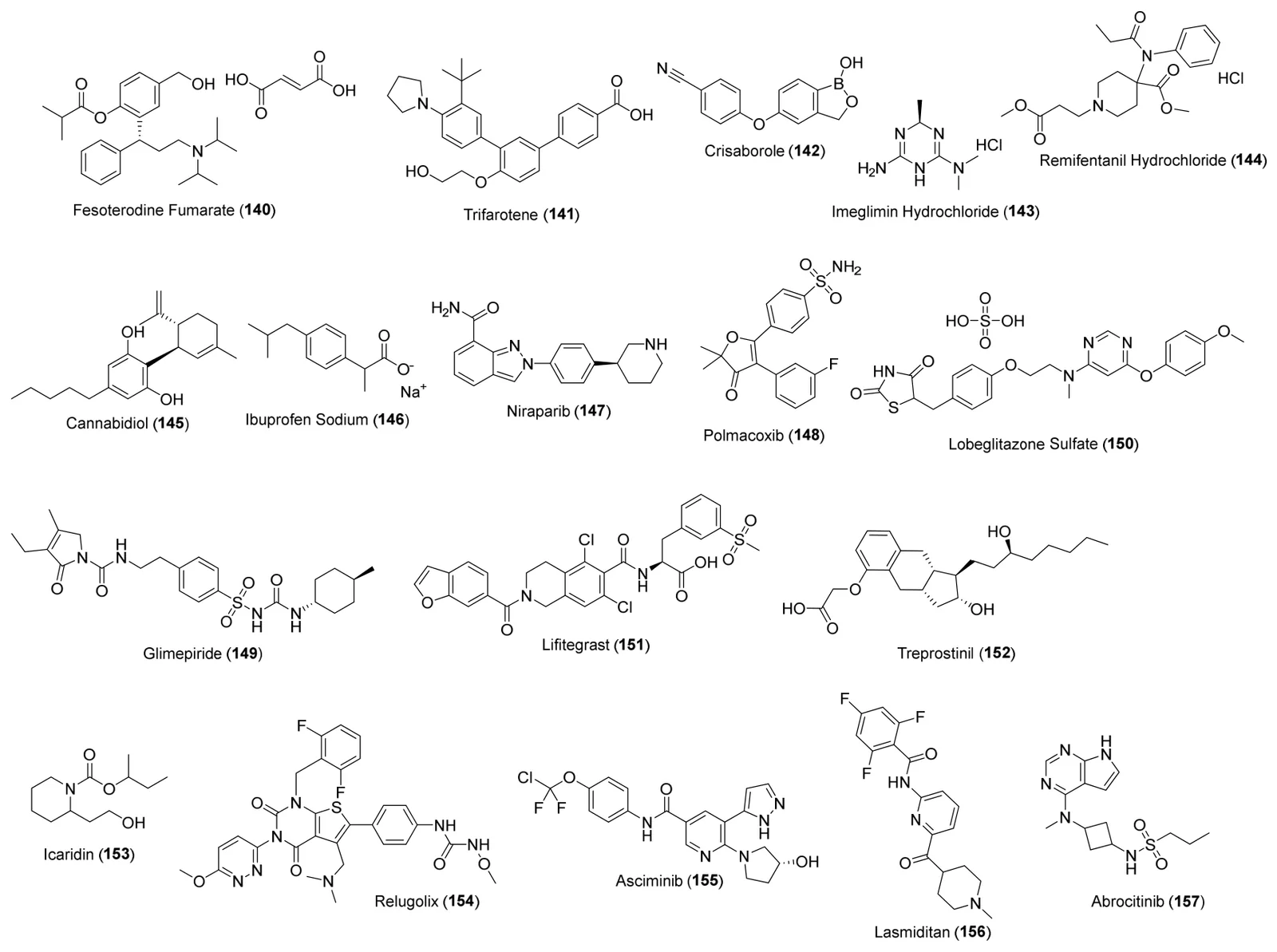
Chemical structures of small molecules approved by CDSCO in 2023.

In 2023, 18 small molecules were approved [13] (Figure 10, Table 19). In the field of urology, overactive bladder (OAB) is commonly managed with antimuscarinic agents such as fesoterodine fumarate (**140**), which reduces detrusor muscle overactivity by blocking muscarinic receptors in the bladder. By decreasing urgency, frequency, and urge incontinence episodes, it improves quality of life in patients with OAB. Within dermatology, several targeted therapies address inflammatory and dermatologic conditions. Trifarotene (**141**) is a fourth-generation topical retinoid selective for RAR-γ receptors, indicated for acne vulgaris and designed to normalize keratinization and reduce inflammation. Crisaborole (**142**) is used in atopic dermatitis but represents a different therapeutic approach. Crisaborole is a topical phosphodiesterase-4 inhibitor that reduces inflammatory cytokine production. Imeglimin hydrochloride (**143**) improves mitochondrial function and enhances both insulin secretion and sensitivity. Remifentanil hydrochloride (**144**) is an ultra-short-acting μ-opioid receptor agonist used during anesthesia because of its rapid onset and metabolism by plasma esterases, allowing precise titration. In neurology, cannabidiol (**145**) has emerged as an adjunctive therapy for severe epileptic syndromes including Lennox-Gastaut syndrome, Dravet syndrome, and tuberous sclerosis complex. Its multimodal mechanism reduces seizure frequency in otherwise refractory epilepsy. For mild-to-moderate pain and fever, Ibuprofen sodium (**146**) provides cyclooxygenase inhibition with faster absorption compared to standard ibuprofen acid formulations. Niraparib (**147**) is a PARP inhibitor used as maintenance therapy in ovarian cancer, exploiting synthetic lethality in tumors with homologous recombination deficiency. In musculoskeletal disorders, polmacoxib (**148**) is used in osteoarthritis management. As a selective COX-2 inhibitor with carbonic anhydrase binding properties, it provides anti-inflammatory and analgesic effects while aiming to reduce gastrointestinal adverse effects compared to nonselective NSAIDs. Traditional sulfonylureas such as glimepiride (**149**) stimulate pancreatic insulin release, while thiazolidinedione derivatives like Lobeglitazone sulfate (**150**) improve peripheral insulin sensitivity through PPAR-γ activation. In ophthalmology, lifitegrast (**151**) is indicated for dry eye disease. It blocks LFA-1 interaction with ICAM-1, reducing T-cell-mediated inflammation on the ocular surface. Treprostinil (**152**) is a prostacyclin analog indicated for pulmonary arterial hypertension. By promoting vasodilation and inhibiting platelet aggregation, it reduces pulmonary vascular resistance and improves exercise capacity. Icaridin (**153**, also known as picaridin) is not a therapeutic drug but an effective mosquito repellent used to prevent vector-borne diseases such as malaria, dengue, and Zika virus infection. Relugolix (**154**) is an oral GnRH receptor antagonist used in advanced prostate cancer to achieve rapid testosterone suppression without the initial flare associated with GnRH agonists. Asciminib (**155**) is a first-in-class STAMP inhibitor targeting the ABL myristoyl pocket in Philadelphia chromosome-positive chronic myeloid leukemia, offering an option for patients resistant to prior tyrosine kinase inhibitors. For acute migraine treatment, Lasmiditan (**156**) is a selective 5-HT1F receptor agonist that provides relief without vasoconstrictive effects, making it suitable for patients with cardiovascular risk factors. Abrocitinib (**157**) is an oral medication used to treat moderate-to-severe atopic dermatitis by reducing inflammation and itching.

Overall, these medications illustrate the diversity of modern pharmacotherapy, ranging from symptomatic relief to highly specific molecular targeting. Many reflect a broader trend toward precision medicine, selective receptor modulation, and improved safety profiles tailored to specific disease mechanisms.

## 4. Concluding Remarks

The 2025 CDSCO approval landscape signals a maturing phase of India’s regulatory and therapeutic ecosystem, increasingly focused on measurable patient impact rather than novelty alone. The therapies cleared this year exemplify how “revolutionary” innovation in the Indian context is defined by scalability, durability of benefit, and system-level relief, alongside scientific sophistication. Targeted oncology agents, next-generation immunomodulators, and disease-modifying therapies for chronic conditions collectively point to a future in which serious illness is managed earlier, more precisely, and with reduced downstream burden on hospitals and specialists.

However, the true success of these approvals will be determined beyond regulatory clearance. Pricing strategies, domestic manufacturing, diagnostic readiness, physician training, and public–private coordination will ultimately decide whether these therapies translate into widespread clinical benefit. As India balances cutting-edge innovation with population-scale healthcare delivery, 2025 may be remembered not just as a year of important approvals, but as a test case for how effectively high-impact medicines can be operationalized to transform outcomes for millions of patients.

Going into detail, the 2025 CDSCO approvals show a clear dominance of oncology-focused small molecules, reflecting the continued prioritization of targeted cancer therapies in drug development and regulatory approvals. The concentration of kinase inhibitors and mutation-specific agents underscores the shift toward precision oncology, while non-oncology approvals remain more evenly distributed across chronic therapeutic areas such as neurology, immunology, and gastroenterology. Looking forward, the CDSCO’s actions will be instrumental in shaping India’s role on the global pharmaceutical stage. By demonstrating the capacity to efficiently and rigorously evaluate complex targeted therapies, the organization is building a crucial pillar of trust. This will not only accelerate the introduction of novel drugs for Indian patients but will also position India as an increasingly attractive destination for global clinical trials and a hub for the manufacturing of next-generation precision medicines.

## Figures and Tables

**Table 1:** The isosurface of tafamidis calculated using the Rowan online tool [25]. Physicochemical properties calculated using SwissADME (https://www.swissadme.ch/) [26].

Isosurface (electron density) ^a^ 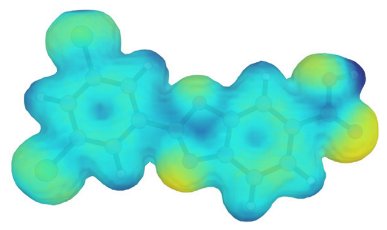
Physicochemical properties TPSA: 63.33 Å^2^ Consensus Log Po/w: 3.76 Synthetic accessibility ^b^: 2.67

^a^ Negative values are shown in orange–red; positive values are in blue. ^b^ From 1 (easy) to 10 (very difficult).

**Table 2:** The isosurface of letermovir calculated using the Rowan online tool [25]. Physicochemical properties calculated using SwissADME [26].

Isosurface (electron density) ^a^ 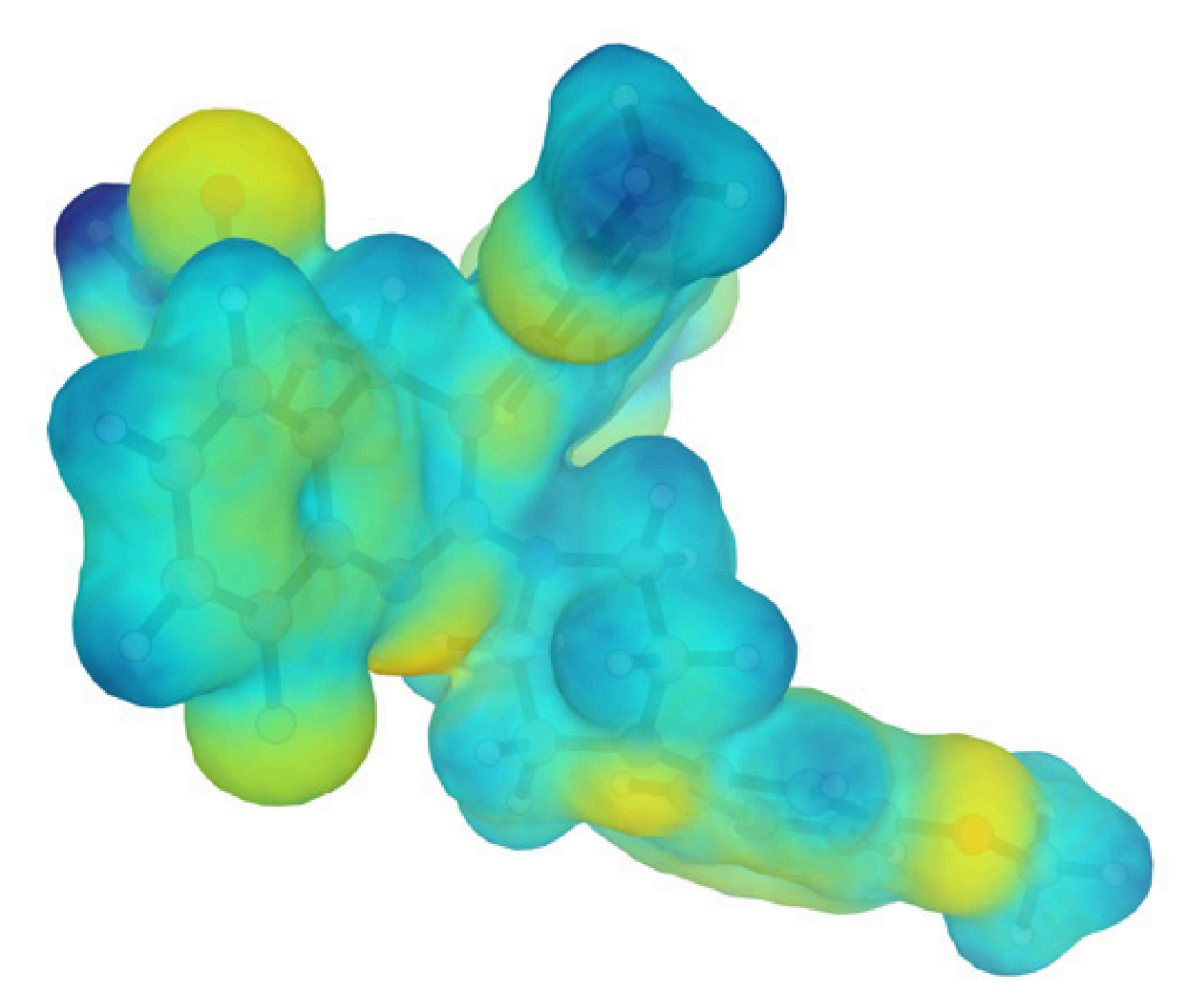
Physicochemical properties TPSA: 77.84 Å^2^ Consensus Log Po/w: 4.57 Synthetic accessibility ^b^: 4.97

^a^ Negative values are shown in orange–red; positive values are in blue. ^b^ From 1 (easy) to 10 (very difficult).

**Table 3:** The isosurface of fexuprazan calculated using the Rowan online tool [25]. Physicochemical properties calculated using SwissADME [26].

Isosurface (electron density) ^a^ 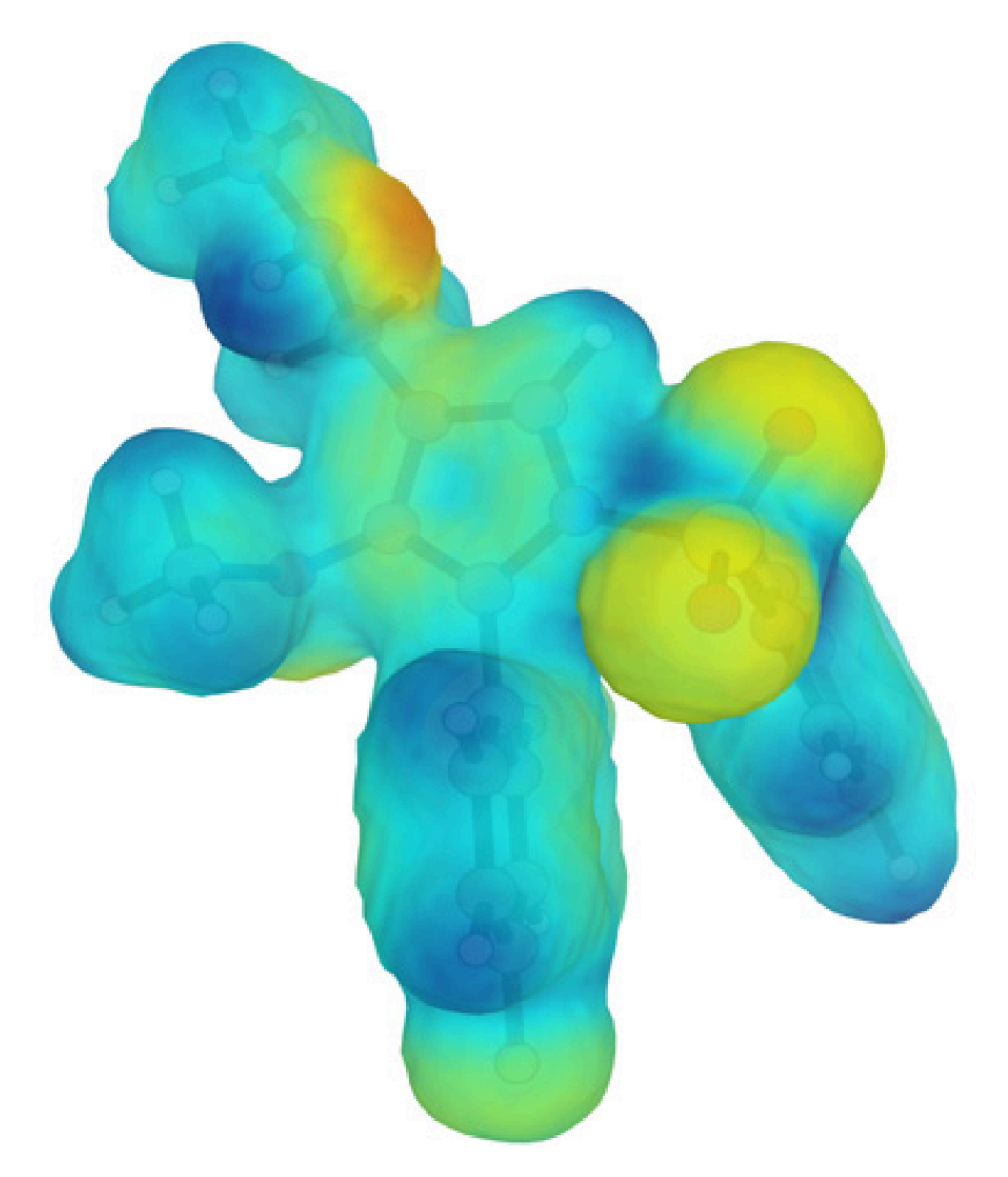
Physicochemical properties TPSA: 68.71 Å^2^ Consensus Log Po/w: 3.95 Synthetic accessibility ^b^: 3.54

^a^ Negative values are shown in orange–red; positive values are in blue. ^b^ From 1 (easy) to 10 (very difficult).

**Table 4:** The isosurface of edoxaban calculated using the Rowan online tool [25]. Physicochemical properties calculated using SwissADME [26].

Isosurface (electron density) ^a^ 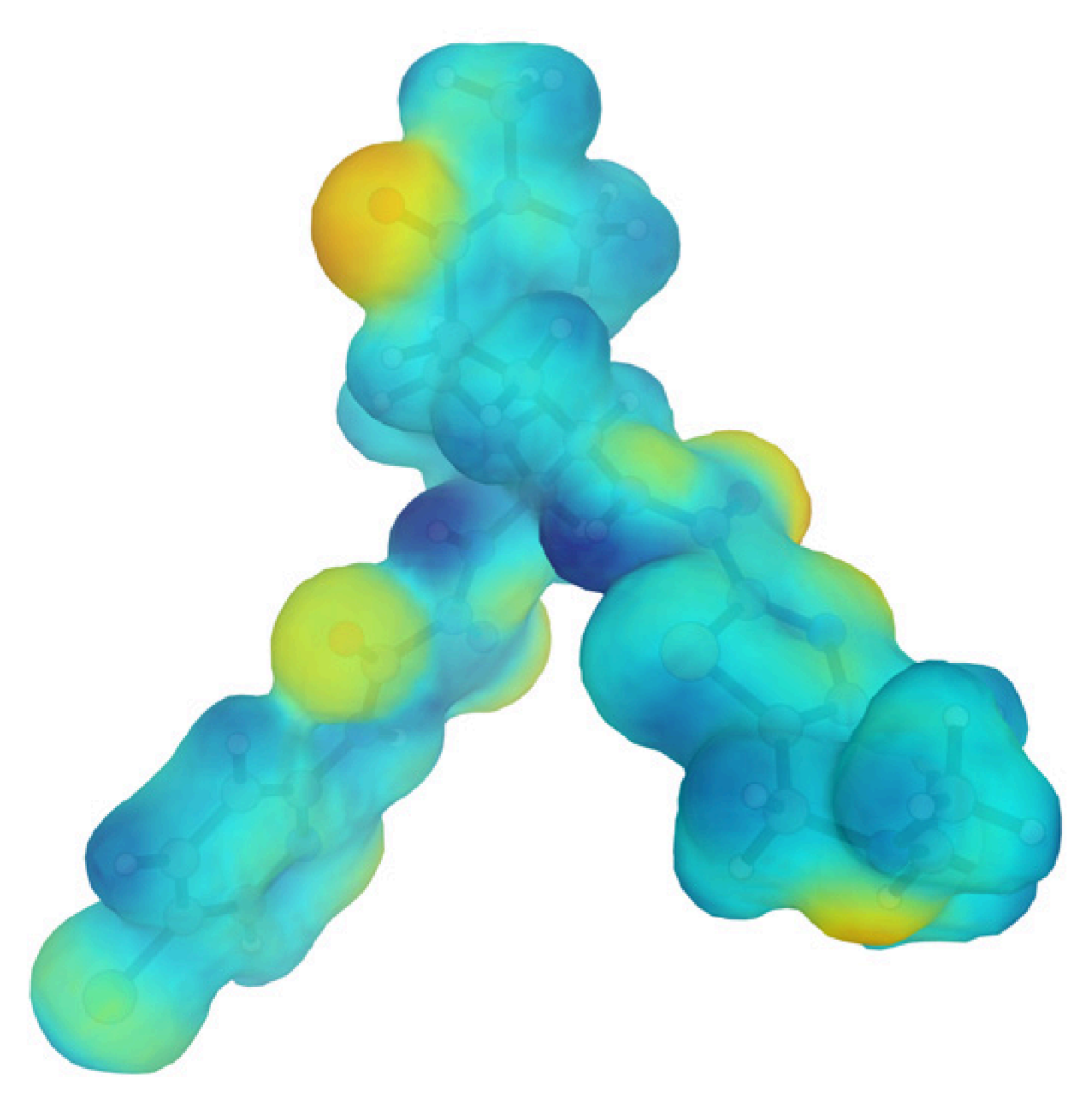
Physicochemical properties TPSA: 164.87 Å^2^ Consensus Log Po/w: 1.34 Synthetic accessibility ^b^: 5.04

^a^ Negative values are shown in orange–red; positive values are in blue. ^b^ From 1 (easy) to 10 (very difficult).

**Table 5:** The isosurface of rimegepant calculated using the Rowan online tool [25]. Physicochemical properties calculated using SwissADME [26].

Isosurface (electron density) ^a^ 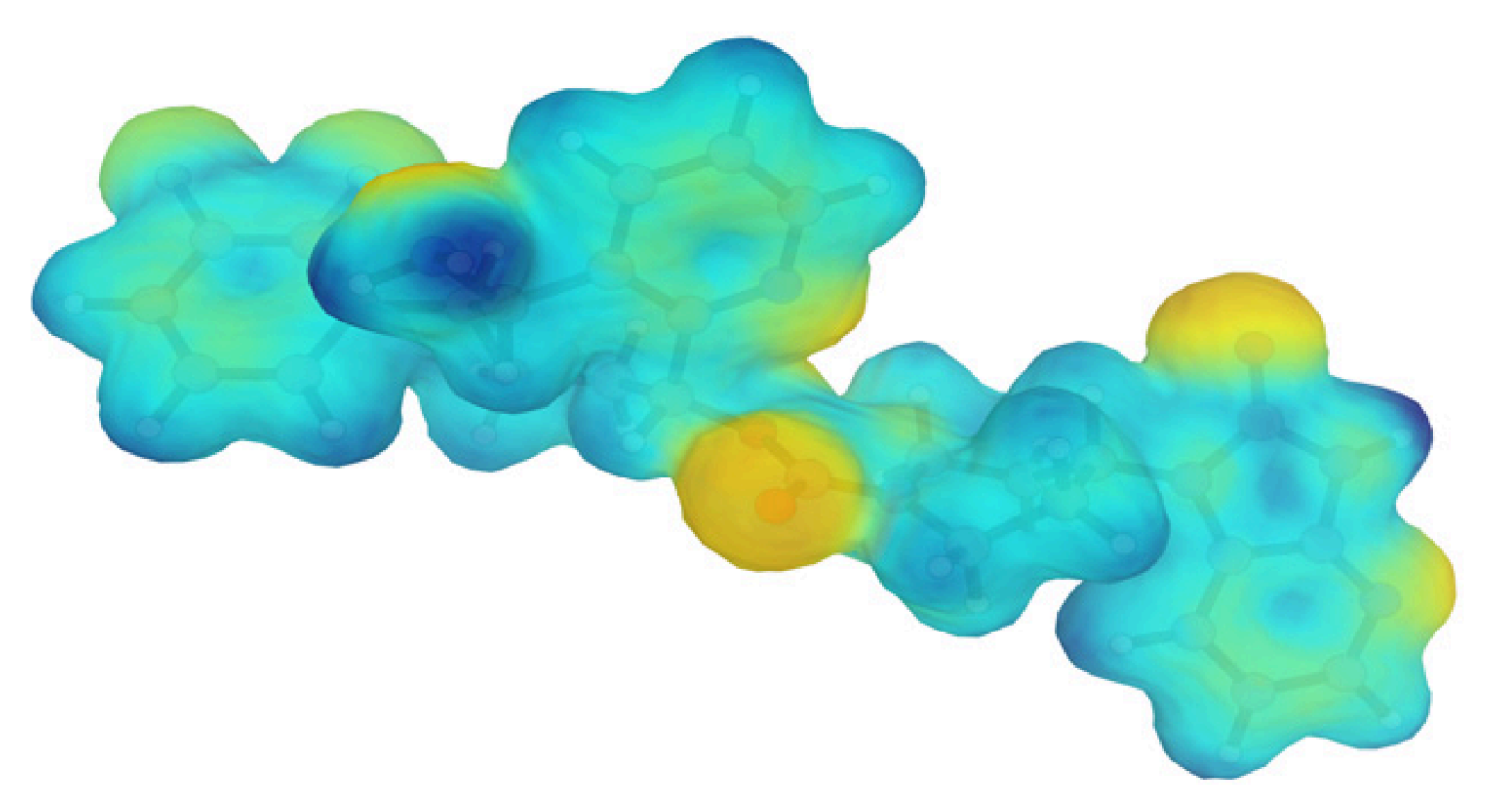
Physicochemical properties TPSA: 119.13 Å^2^ Consensus Log Po/w: 3.10 Synthetic accessibility ^b^: 5.20

^a^ Negative values are shown in orange–red; positive values are in blue. ^b^ From 1 (easy) to 10 (very difficult).

**Table 6:** The isosurface of doravirine calculated using the Rowan online tool [25]. Physicochemical properties calculated using SwissADME [26].

Isosurface (electron density) ^a^ 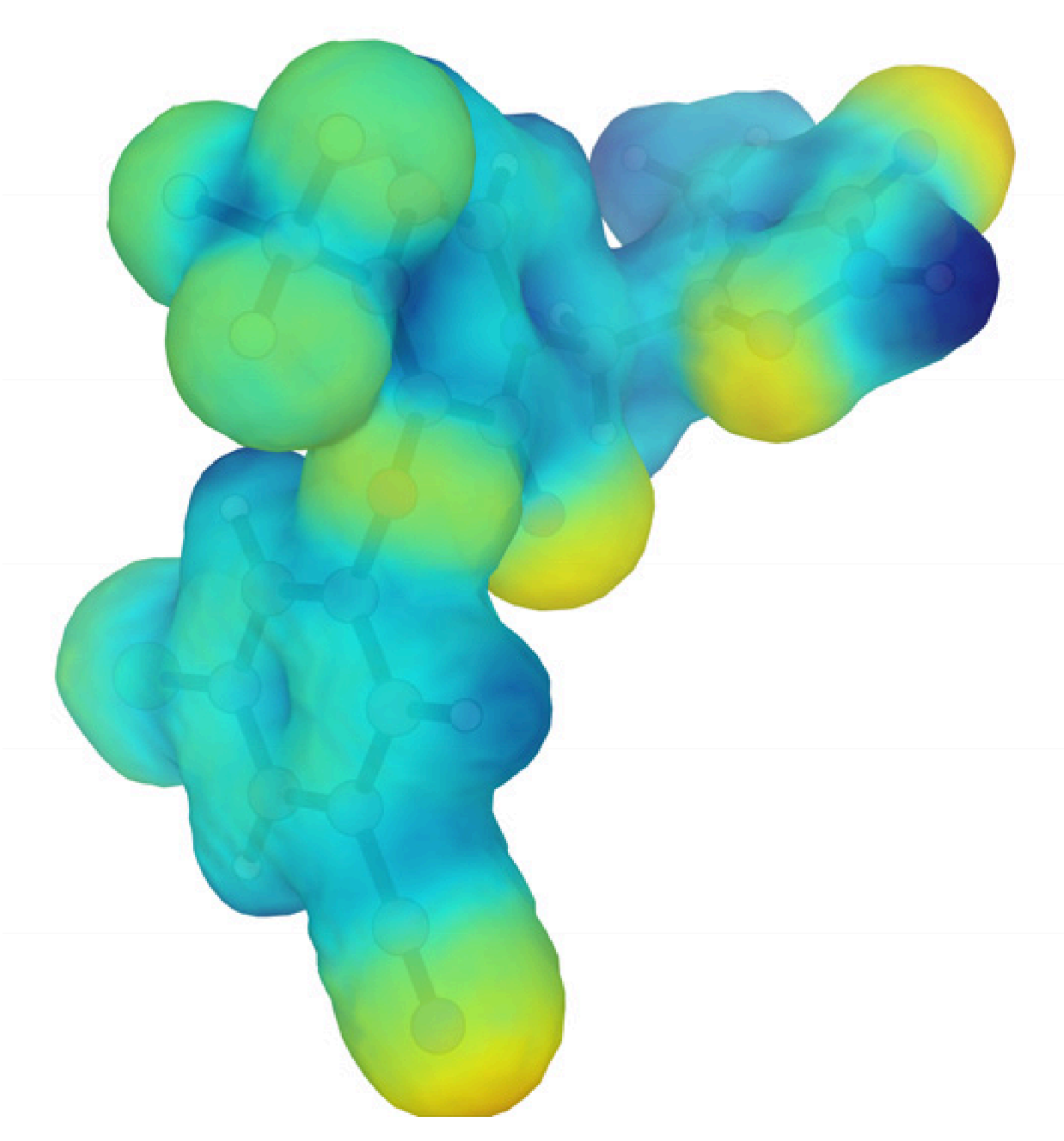
Physicochemical properties TPSA: 105.70 Å^2^ Consensus Log Po/w: 2.75 Synthetic accessibility ^b^: 3.28

^a^ Negative values are shown in orange–red; positive values are in blue. ^b^ From 1 (easy) to 10 (very difficult).

**Table 7:** The isosurface of tucatinib calculated using the Rowan online tool [25]. Physicochemical properties calculated using SwissADME [26].

Isosurface (electron density) ^a^ 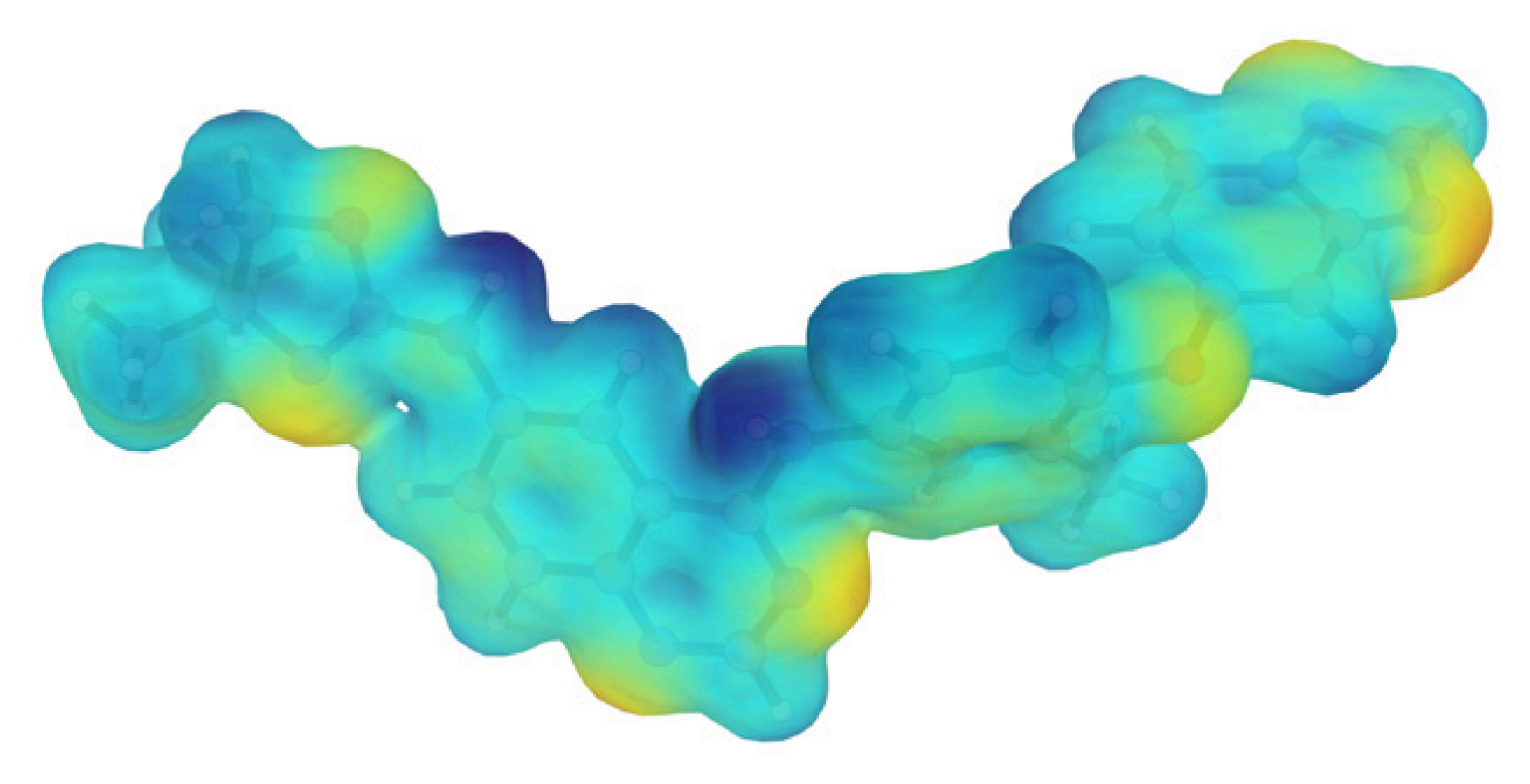
Physicochemical properties TPSA: 110.85 Å^2^ Consensus Log Po/w: 3.77 Synthetic accessibility ^b^: 4.01

^a^ Negative values are shown in orange–red; positive values are in blue. ^b^ From 1 (easy) to 10 (very difficult).

**Table 8:** The isosurface of zanubrutinib calculated using the Rowan online tool [25]. Physicochemical properties calculated using SwissADME [26].

Isosurface (electron density) ^a^ 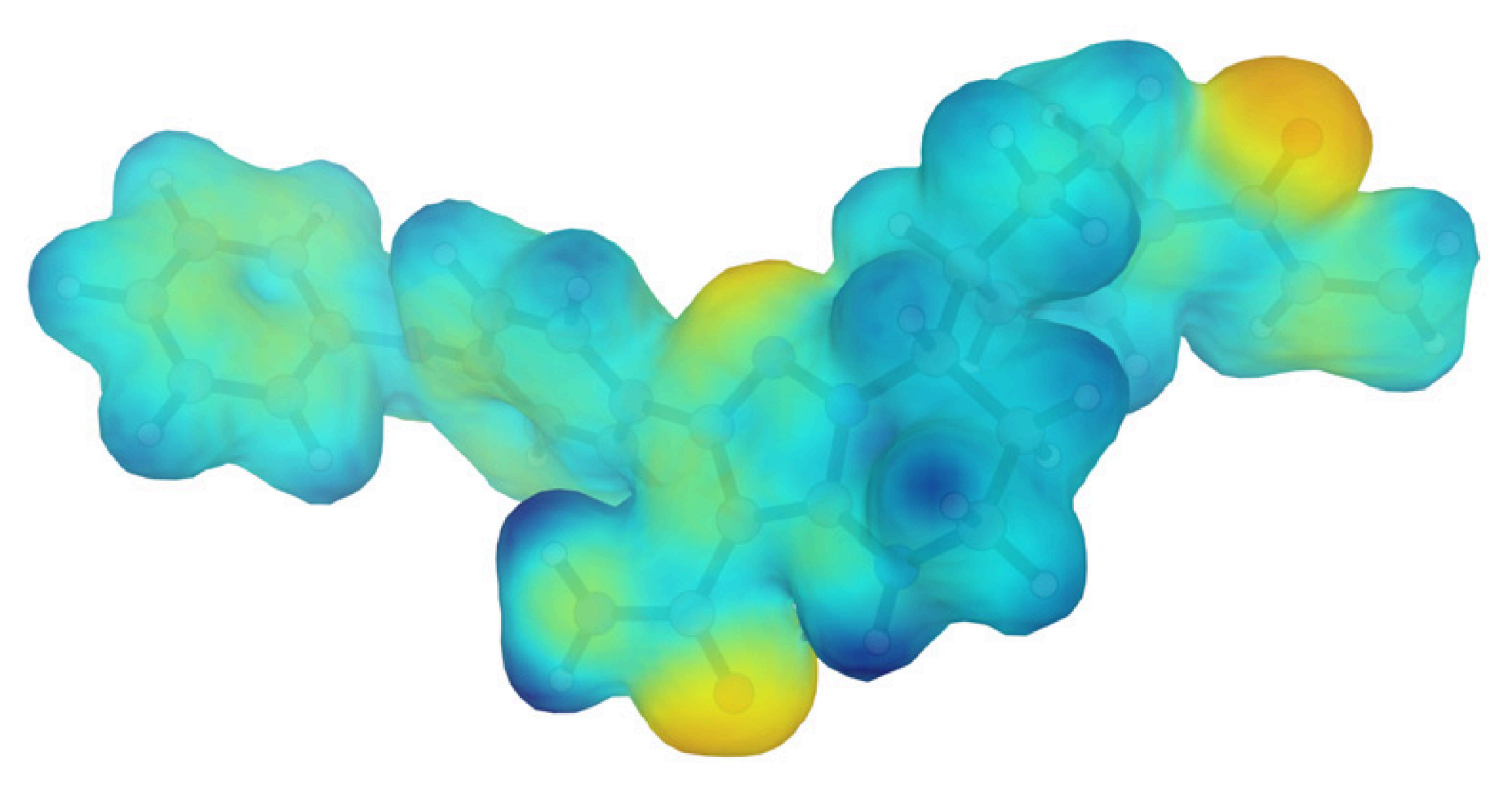
Physicochemical properties TPSA: 102.48 Å^2^ Consensus Log Po/w: 3.17 Synthetic accessibility ^b^: 4.32

^a^ Negative values are shown in orange–red; positive values are in blue. ^b^ From 1 (easy) to 10 (very difficult).

**Table 9:** The isosurface of siponimod calculated using the Rowan online tool [25]. Physicochemical properties calculated using SwissADME [26].

Isosurface (electron density) ^a^ 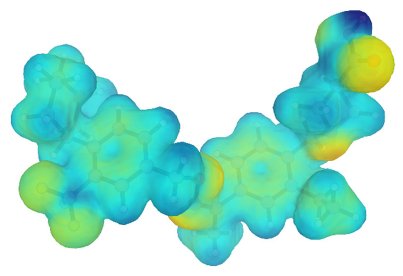
Physicochemical properties TPSA: 62.13 Å^2^ Consensus Log Po/w: 5.79 Synthetic accessibility ^b^: 4.63

^a^ Negative values are shown in orange–red; positive values are in blue. ^b^ From 1 (easy) to 10 (very difficult).

**Table 10:** The isosurface of ivosidenib calculated using the Rowan online tool [25]. Physicochemical properties calculated using SwissADME [26].

Isosurface (electron density) ^a^ 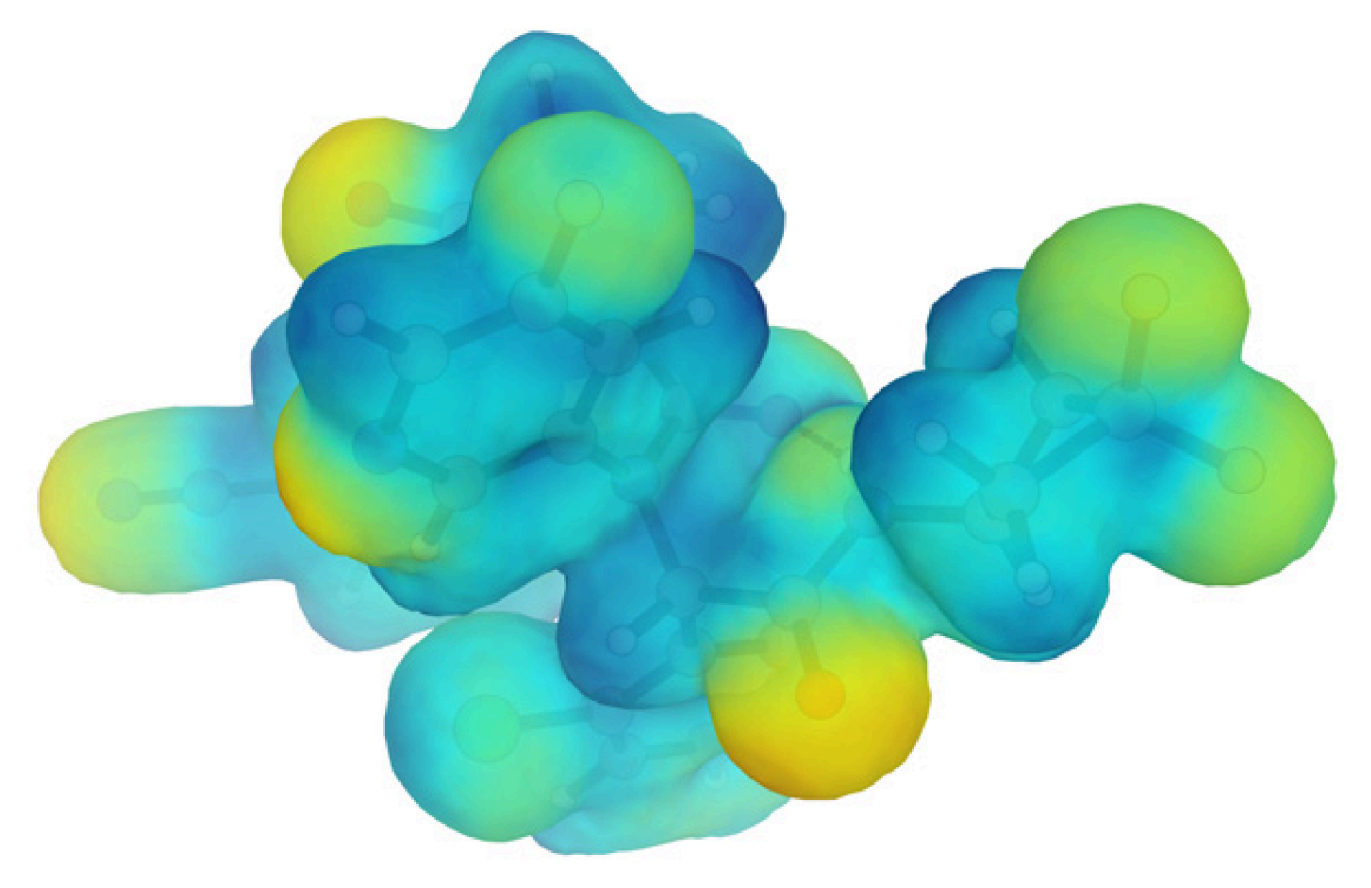
Physicochemical properties TPSA: 119.29 Å^2^ Consensus Log Po/w: 3.40 Synthetic accessibility ^b^: 4.42

^a^ Negative values are shown in orange–red; positive values are in blue. ^b^ From 1 (easy) to 10 (very difficult).

**Table 11:** The isosurface of tegoprazan calculated using the Rowan online tool [25]. Physicochemical properties calculated using SwissADME [26].

Isosurface (electron density) ^a^ 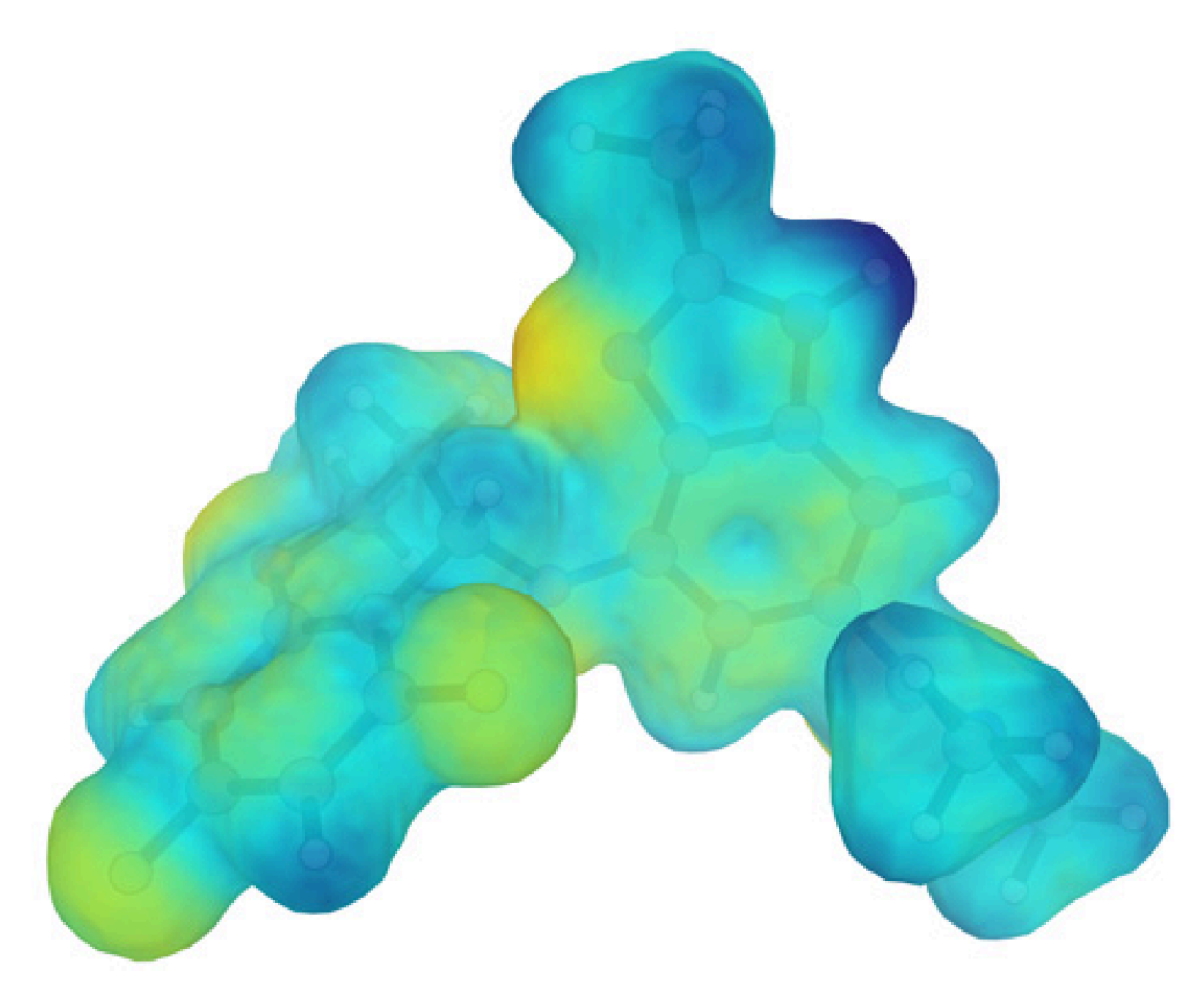
Physicochemical properties TPSA: 67.45 Å^2^ Consensus Log Po/w: 3.44 Synthetic accessibility ^b^: 3.59

^a^ Negative values are shown in orange–red; positive values are in blue. ^b^ From 1 (easy) to 10 (very difficult).

**Table 12:** The isosurface of upadacitinib calculated using the Rowan online tool [25]. Physicochemical properties calculated using SwissADME [26].

Isosurface (electron density) ^a^ 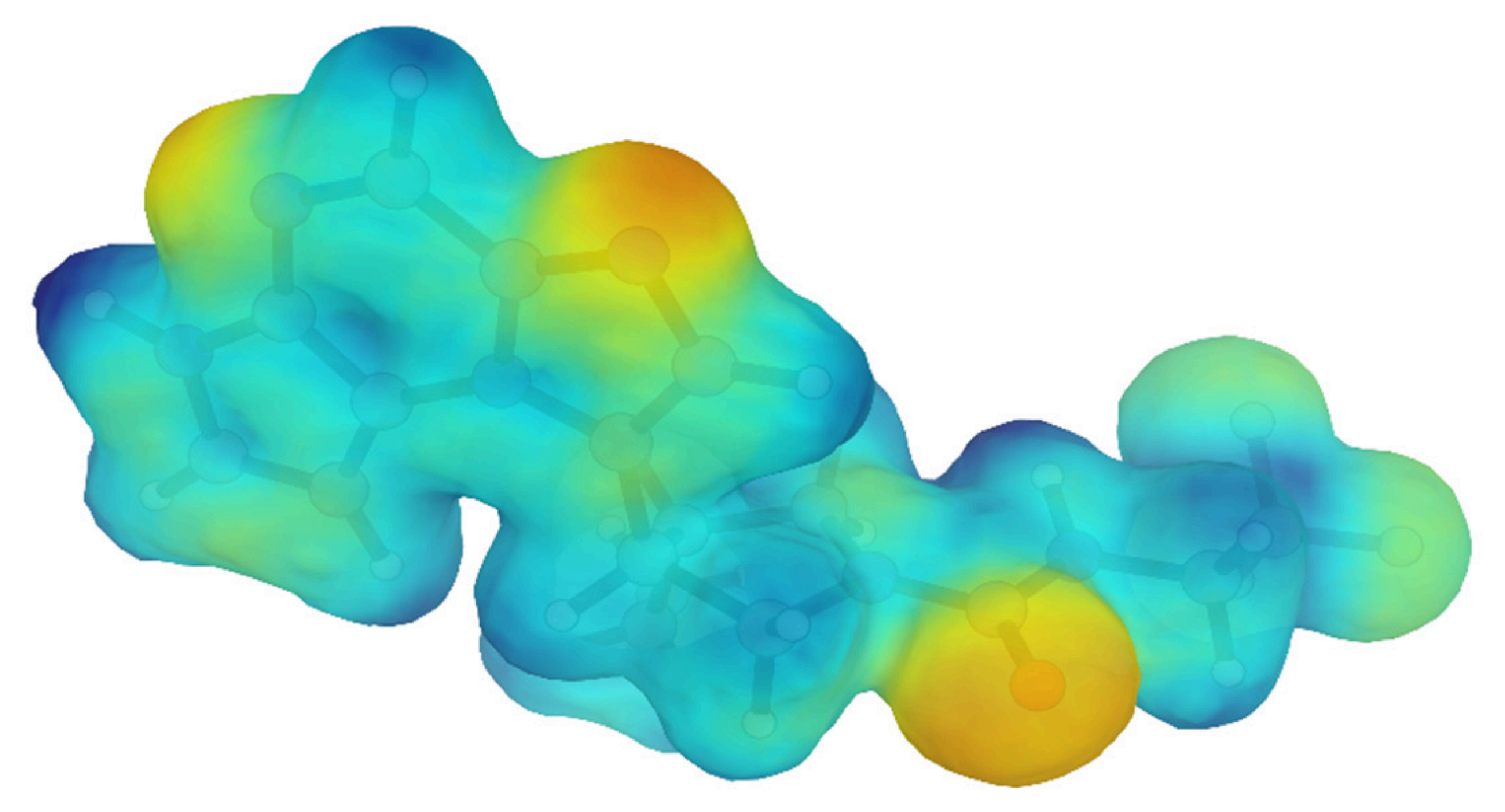
Physicochemical properties TPSA: 78.32 Å^2^ Consensus Log Po/w: 2.48 Synthetic accessibility ^b^: 3.75

^a^ Negative values are shown in orange–red; positive values are in blue. ^b^ From 1 (easy) to 10 (very difficult).

**Table 13:** The isosurface of pitolisant calculated using the Rowan online tool [25]. Physicochemical properties calculated using SwissADME [26].

Isosurface (electron density) ^a^ 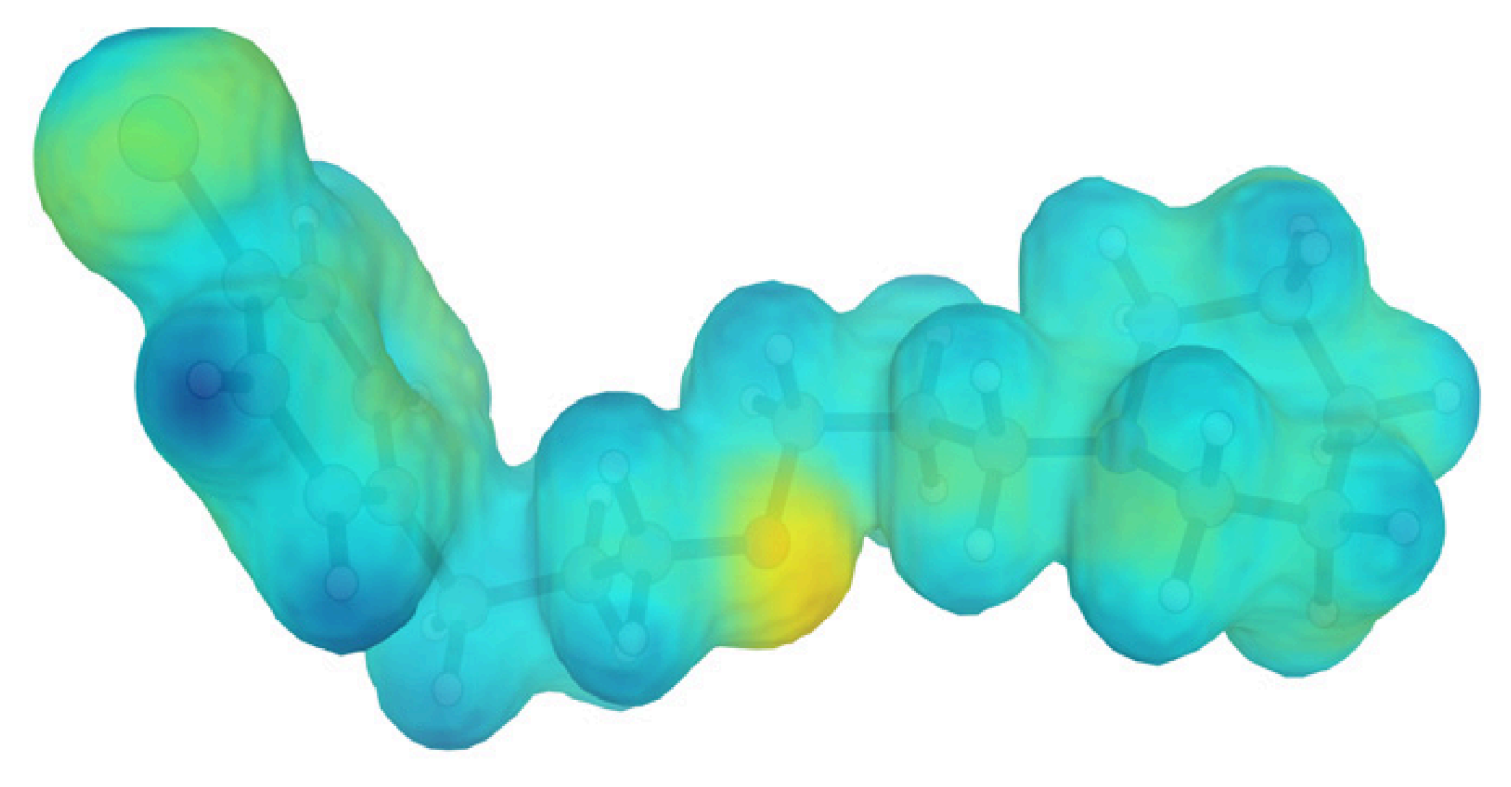
Physicochemical properties TPSA: 12.47 Å^2^ Consensus Log Po/w: 4.09 Synthetic accessibility ^b^: 2.21

^a^ Negative values are shown in orange–red; positive values are in blue. ^b^ From 1 (easy) to 10 (very difficult).

**Table 14:** The isosurface of etrasimod calculated using the Rowan online tool [25]. Physicochemical properties calculated using SwissADME [26].

Isosurface (electron density) ^a^ 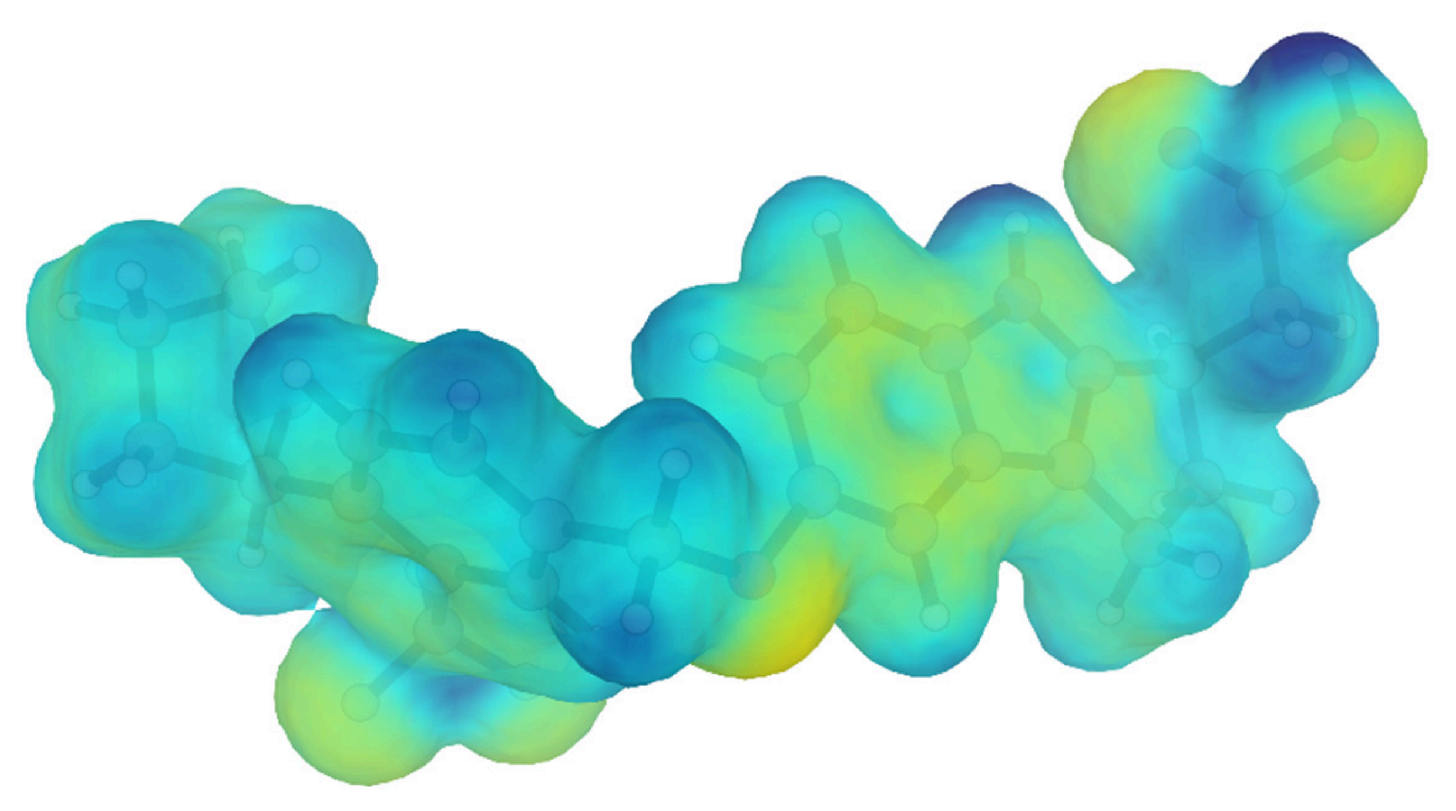
Physicochemical properties TPSA: 62.32 Å^2^ Consensus Log Po/w: 5.94 Synthetic accessibility ^b^: 3.89

^a^ Negative values are shown in orange–red; positive values are in blue. ^b^ From 1 (easy) to 10 (very difficult).

**Table 15:** The isosurface of erdafitinib calculated using the Rowan online tool [25]. Physicochemical properties calculated using SwissADME [26].

Isosurface (electron density) ^a^ 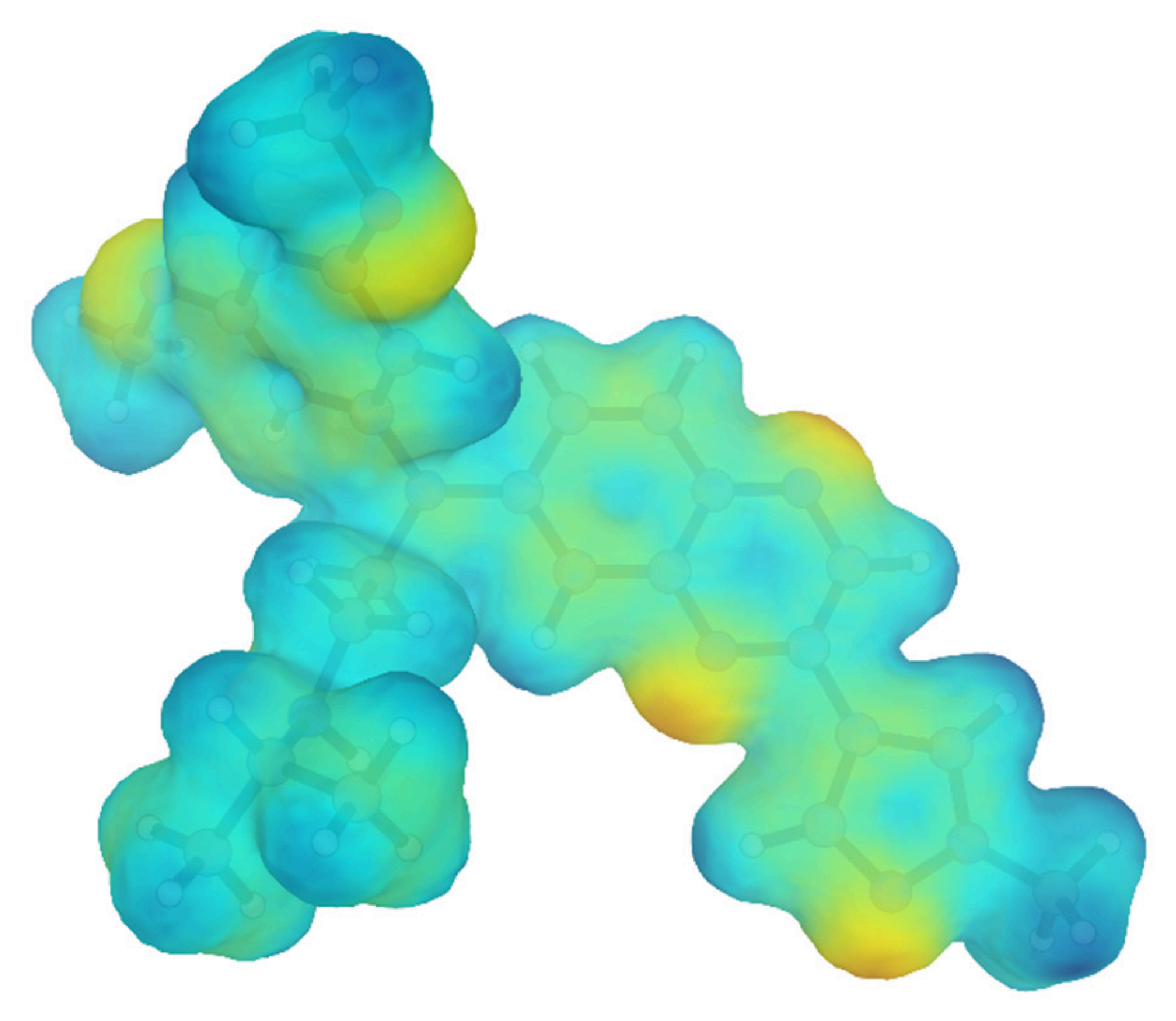
Physicochemical properties TPSA: 77.33 Å^2^ Consensus Log Po/w: 3.29 Synthetic accessibility ^b^: 3.66

^a^ Negative values are shown in orange–red; positive values are in blue. ^b^ From 1 (easy) to 10 (very difficult).

**Table 16:** The isosurface of vorasidenib calculated using the Rowan online tool [25]. Physicochemical properties calculated using SwissADME [26].

Isosurface (electron density) ^a^ 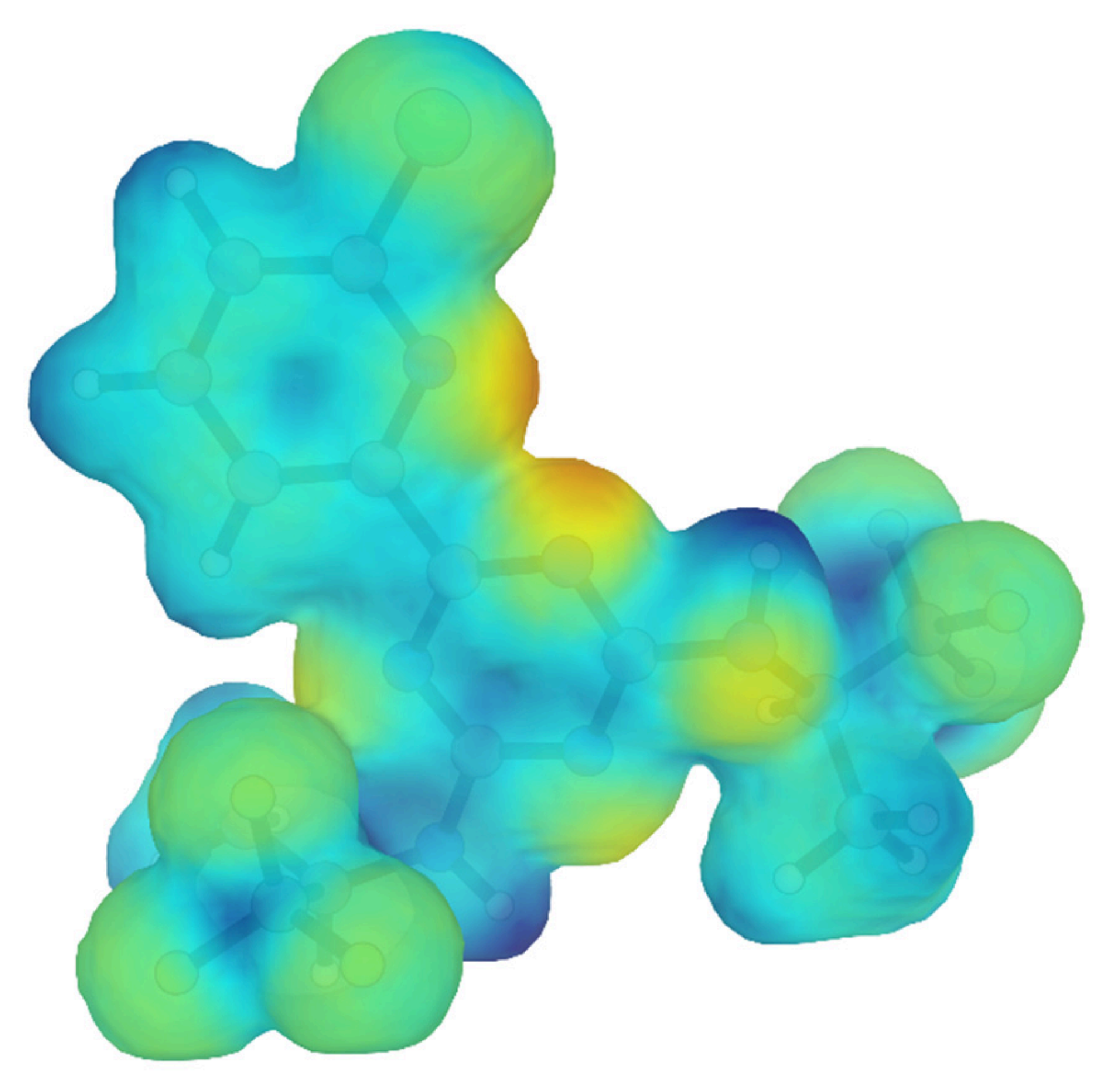
Physicochemical properties TPSA: 75.62 Å^2^ Consensus Log Po/w: 4.11 Synthetic accessibility ^b^: 3.48

^a^ Negative values are shown in orange–red; positive values are in blue. ^b^ From 1 (easy) to 10 (very difficult).

**Table 17:** Therapeutic area-wise classification of CDSCO-approved small molecules in 2025.

Therapeutic Area	Drug	Primary Indication	Mechanism/Class
Oncology/Hematology	Ivosidenib (**10**)	AML, cholangiocarcinoma	IDH1 inhibitor [98]
	Erdafitinib (**19**)	Urothelial carcinoma	FGFR tyrosine kinase inhibitor [146]
	Vorasidenib (**20**)	Glioma	Dual IDH1/IDH2 inhibitor [151]
	Tucatinib (**7**)	HER2-positive breast cancer	HER2-selective TKI [79]
	Zanubrutinib (**8**)	B-cell malignancies	BTK inhibitor [86]
Cardiovascular/Hematology (Non-oncology)	Tafamidis (**1**)	Transthyretin amyloid cardiomyopathy	TTR stabilizer [19]
	Edoxaban (**4**)	Thromboembolism prevention	Factor Xa inhibitor [53]
Neurology	Rimegepant (**5**)	Migraine	CGRP receptor antagonist [63]
	Siponimod (**9**)	Multiple sclerosis	S1P receptor modulator [94]
	Pitolisant (**17**)	Narcolepsy	Histamine H3 receptor antagonist/inverse agonist [132]
Infectious Diseases	Letermovir (**2**)	CMV prophylaxis	CMV terminase complex inhibitor [32]
	Doravirine (**6**)	HIV-1 infection	NNRTI
Immunology/Autoimmune	Upadacitinib (**16**)	RA, IBD, other immune disorders	JAK1 inhibitor [127]
	Etrasimod (**18**)	Ulcerative colitis	S1P receptor modulator [139]
Gastroenterology	Tegoprazan (**11**)	Acid-related disorders	Potassium-competitive acid blocker (P-CAB) [105]
	Fexuprazan (**3**)	Acid-related disorders	Potassium-competitive acid blocker (P-CAB) [45]
Other/Metabolic	Stamicis (**15**)	Used to prepare a diagnostic imaging agent	Other
	Combination of relugolix (**12**), estradiol (**13**) and norethindrone (**14**)	Heavy menstrual bleeding associated with uterine Leiomyomas (Fibroids) [112]	Multiple receptors

**Table 18:** Therapeutic area-wise classification of CDSCO-approved small molecules in 2024.

Therapeutic Area	Drug	Primary Indication
Oncology/Hematology	Selpercatinib (**124**) Nelarabine (**125**) Brigatinib (**135**) Belumosudil (**137**)	RET-altered cancers (e.g., NSCLC, thyroid cancer) [160] T-cell acute lymphoblastic leukemia/lymphoma [161] ALK-positive non-small cell lung cancer [162] Chronic graft-versus-host disease (post-transplant; immune-hematologic overlap but commonly grouped with hem/onc) [163]
Cardiovascular/Hematology (Non-oncology)	Mavacamten (**136**)	Hypertrophic cardiomyopathy [164]
Neurology	Etifoxine hydrochloride (**122**) Remifentanil hydrochloride (**132**) Pimavanserin tartrate (**133**) Lumateperone tosylate (**138**)	Anxiety disorders [165] Short-acting opioid analgesic (anesthesia/sedation) [166] Parkinson’s disease psychosis [167] Schizophrenia/bipolar depression [168]
Infectious Diseases	Plazomicin (**121**) Enmetazobactam (**126**) + cefepime (**127**) Methenamine hippurate (**128**) Tedizolid phosphate (**131**)	Complicated urinary tract infections [169] Complicated UTIs (β-lactam antibiotic + β-lactamase inhibitor [170] UTI prophylaxis [171] Acute bacterial skin/soft tissue infections [172]
Immunology/Autoimmune		
Gastroenterology	Vonoprazan fumarate (**123**) Elobixibat hydrate (**130**)	GERD, peptic ulcer disease [173] Chronic constipation [174]
Other/Metabolic	Sodium phenylbutyrate (**129**) Elagolix sodium (**134**) Trelagliptin succinate (**139**)	Urea cycle disorders [175] Endometriosis (gynecologic) [176] Type 2 diabetes mellitus [177]

**Table 19:** Therapeutic area-wise classification of CDSCO-approved small molecules in 2023.

Therapeutic Area	Drug	Primary Indication
Oncology/Hematology	Niraparib (**147**)Relugolix (**154**) Asciminib (**155**)	Ovarian cancer maintenanceAdvanced prostate cancer Ph+ Chronic Myeloid Leukemia (CML)
Cardiovascular/Hematology (Non-oncology)	Treprostinil (**152**)	Pulmonary arterial hypertension
Neurology	Cannabidiol (**145**) Lasmiditan (**156**)	Lennox-Gastaut syndrome, Dravet syndrome, TSC Acute migraine
Infectious Diseases	Icaridin (**153**)	Mosquito repellent (vector-borne disease prevention)
Immunology/Autoimmune	Crisaborole (**142**) Abrocitinib (**157**)	Atopic dermatitis Atopic dermatitis
Gastroenterology	—	—
Other/Metabolic	Fesoterodine fumarate (**140**) Trifarotene (**141**) Imeglimin hydrochloride (**143**) Lobeglitazone sulfate (**150**) Glimepiride (**149**) Remifentanil hydrochloride (**144**) Ibuprofen sodium (**146**) Polmacoxib (**148**) Lifitegrast (**151**)	Overactive bladder (OAB) Acne vulgaris Type 2 diabetes mellitus Type 2 diabetes mellitus Type 2 diabetes mellitus Analgesic during anesthesia Pain and fever Osteoarthritis Dry eye disease

## Data Availability

No new data were created or analyzed in this study. Data sharing is not applicable to this article.

## Supplementary Materials

The following supporting information can be downloaded at: https://www.mdpi.com/article/10.3390/molecules31213962/s1, Figure S1: Worldwide sales (millions of USD) in 2025 for CDSCO-approved drugs described in the paper; Table S1: Details for CDSCO-approved small molecules in 2025.

## Abbreviations

The following abbreviations are used in this manuscript:

**Abbreviation**	**Full name**
**(*****o*****-MeC_6_H_4_)_3_P**	Tri(*o*-tolyl)phosphine
**Ac_2_O**	Acetic anhydride
**ADDP**	1,1′-(Azodicarbonyl)dipiperidine
**AIBN**	Azobisisobutyronitrile
**AlCl_3_**	Aluminum chloride
**BTK**	Bruton’s tyrosine kinase
**CCl_4_**	Carbon tetrachloride
**CDI**	1,1′-Carbonyldiimidazole
**CDSCO**	Central Drugs Standard Control Organization
**CF_3_CH_2_OH**	2,2,2-Trifluoroethanol
**CGRP**	Calcitonin gene-related peptide
**CMV**	Cytomegalovirus
**CYP**	Cytochrome P450
**DBU**	1,8-Diazabicyclo[5.4.0]undec-7-ene
**DCM**	Dichloromethane
**DIPEA**	*N*,*N*-Diisopropylethylamine
**DMA**	*N*,*N*-Dimethylacetamide
**DMF-DMA**	Dimethylformamide dimethyl acetal
**ECG**	Electrocardiogram
**EDC**	1-Ethyl-3-(3-dimethylaminopropyl)carbodiimide
**FDA**	Food and Drug Administration
**FGFR**	Fibroblast growth factor receptor
**GERD**	Gastroesophageal reflux disease
**HBTU**	(2-(1H-benzotriazol-1-yl)-1,1,3,3-tetramethyluronium hexafluorophosphate)
**HCOOEt**	Ethyl formate
**HER1/EGFR**	Human epidermal growth factor receptor-1/epidermal growth factor receptor
**HIV**	Human immunodeficiency cirus
**HOBt**	1-Hydroxybenzotriazole
**IDH1**	Isocitrate dehydrogenase-1
**INR**	International normalized ratio
**IPAc**	Isopropyl acetate
**LiHMDS**	Lithium bis(trimethylsilyl)amide
**LiOH**	Lithium hydroxide
**MeSO_3_H**	Methanesulfonic acid
**MnO_2_**	Manganese dioxide
***n*Bu_4_N^+^Br^−^**	Tetrabutylammonium bromide
**N_2_H_4_**	Hydrazine
**Na_2_CO_3_**	Sodium carbonate
**NaBH_4_**	Sodium borohydride
**NaHMDS**	Sodium bis(trimethylsilyl)amide
**NaI**	Sodium iodide
**NaN_3_**	Sodium azide
**NBS**	*N*-Bromosuccinimide
***n*Bu_3_P**	Tributylphosphine
**NCS**	*N*-Chlorosuccinimide
**NDCTR**	New Drugs and Clinical Trials Rules
**NNRTIs**	Non-nucleoside reverse transcriptase inhibitors
**P(CH_3_)_3_**	Trimethylphosphine
**P-CAB**	Potassium-competitive acid blocker
**Pd(OAc)_2_**	Palladium(II) acetate
**Pd(OH)_2_**	Palladium(II) hydroxide
**Pd(PPh_3_)_4_**	Tetrakis(triphenylphosphine)palladium(0)
**Pd_2_(dba)_3_**	Tris(dibenzylideneacetone)dipalladium(0)
**PdCl_2_(CH_3_CN)_2_**	Bis(acetonitrile)palladium(II) dichloride
**PdCl_2_(PPh_3_)_2_**	Bis(triphenylphosphine)palladium(II) dichloride
**PhCH_2_Cl**	Benzyl chloride
**POCl_3_**	Phosphorus oxychloride
**PPh_3_**	Triphenylphosphine
**PPIs**	Proton pump inhibitors
**S1P_1_**	Sphingosine 1-phosphate receptor subtype 1
**SAR**	Structure–activity relationship
**SOCl_2_**	Thionyl chloride
**SPECT**	Single-photon emission computed tomography
***t*-BuOLi**	Lithium *tert*-butoxide
**TBAF**	Tetra-N-butylammonium fluoride
**TCDI**	1,1′-Thiocarbonyldiimidazole
**TEA**	triethylamine
**THF**	Tetrahydrofuran
**TMSCl**	Trimethylsilyl chloride
**TsCl**	Tosyl chloride
**UTI**	Urinary Tract Infection
**Xantphos**	4,5-Bis(diphenylphosphino)-9,9-dimethylxanthene
**Zn(CN)_2_**	Zinc cyanide
