# Supplementary material for: The Indian Pharmaceutical Landscape in 2025: CDSCO-Approved Small Molecules and Synthetic Approaches
Source: Molecules. 2026 Nov 3;31(21):3962. doi: 10.3390/molecules31213962 (PMC13764813; doi:10.3390/molecules31213962)
Supplement: Supplementary file 1 [file molecules-31-03962-s001.zip › molecules-4654272-supplementary.pdf]

SUPPORTING INFORMATION

The Indian Pharmaceutical Landscape in 2025: CDSCO-Approved Small Molecules and Synthetic Approaches

Davide Benedetto Tiz [1,*], Luana Bagnoli [2], Francesca Marini [2], David C. Magri [1] and Luca Sancineto [2,*]

1 Department of Chemistry, Faculty of Science, University of Malta, MSD 2080 Msida, Malta; david.magri@um.edu.mt

2 Group of Catalysis and Organic Green Chemistry, Department of Pharmaceutical Sciences, University of Perugia, Via del Liceo 1, 06100 Perugia, Italy; luana.bagnoli@unipg.it (L.B.); francesca.marini@unipg.it (F.M.)

* Correspondence: davide.benedetto-tiz@um.edu.mt (D.B.T.); luca.sancineto@unipg.it (L.S.)

***Table of contents**:*

page number

Table S1 .... 2/3

Figure S1 3

Computational part 3/4

References 5/6

Table S1. Details for CDSCO-approved small molecules in 2025.

**Drug Name/** ***Commercial name***	**Commercially Stable Form Used in Manufacturing**	**Patent Assignee/related patent**	**Date of approval**	**Clinical indication**	**Revenues worldwide**[a]	**Competitors**
**Tafamidis/** ***Vyndaqel, Vyndamax***	Tafamidis meglumine [1] & Tafamidis free acid	The Scripps Research Institute/Pfizer [2]	16th January 2025	transthyretin (TTR) amyloidosis	6.3 billion	Acoramidis, vutrisiran, patisiran
**Letermovir/** ***Prevymis***	Letermovir (zwitterion free acid)	AiCuris Anti-Infective Cures GmbH [3]	17th January 2025	prevent cytomegalovirus (CMV) infection	978 million	Valganciclovir, ganciclovir
**Fexuprazan/** ***Fexuclue***	Fexuprazan hydrochloride [4]	Daewoong Pharmaceutical Co., Ltd. [5]	10th February 2025	reduce gastric acid secretion	1.15 billion	Vonoprazan, Tegoprazan, Keverprazan, Revaprazan
**Edoxaban/** ***Lixiana, Savaysa***	Edoxaban tosylate monohydrate [6]	Daiichi Sankyo Company, Limited [7]	11th February 2025	anticoagulant	2.5 billion	Apixaban, Rivaroxaban
**Rimegepant/** ***Nurtec***	Rimegepant sulfate [8]	Biohaven/Pfizer [9]	27th March 2025	migraine	1.42 billion	Ubrogepant, atogepant, zavegepant
**Doravirine/** ***Hofix***	Doravirine (anhydrous free base) [10]	Merck Sharp & Dohme LLC [11]	28th March 2025	HIV-1 infection	477 million	Rilpivirine
**Tucatinib/** ***Tukanat***	Tucatinib (free base) [12]	Array BioPharma/Pfizer Inc. [13]	8th April 2025	treat HER2-positive breast cancer	463 million	Neratinib, Lapatinib
**Zanubrutinib/** ***Brukinsa***	Zanubrutinib (free base) [14]	BeOne Medicines I GmbH [15]	8th April 2025	mantle cell lymphoma, chronic lymphocytic leukemia, and Waldenström's macroglobulinemia	3.9 billion	Acalabrutinib, ibrutinib, pirtobrutinib
**Siponimod/** ***Mayzent***	Siponimod fumarate [16]	Novartis AG [17]	9th May 2025	secondary progressive multiple sclerosis	780 million	Ponesimod, Ozanimod, Fingolimod
**Ivosidenib/** ***Tibsovo***	Ivosidenib (free base) [18], [19]	Agios Pharmaceuticals, Inc./Servier [20]	14th May 2025	leukemias and solid tumors	between $150 million and $196 million	Olutasidenib
**Tegoprazan/** ***PCAB***	Tegoprazan (free base) [21]	RaQualia Pharma Inc. [22]	28th May 2025	gastroesophageal reflux disease and peptic ulcers	between US$ 137 million and US$ 140 million	Fexuprazan, Keverprazan, Revaprazan
**Technetium (99mTc) Sestamibi/** ***Stamicis***	Not available	Not available	23rd July 2025	diagnostic imaging agent	Not available	Cardiolite
**Upadacitinib/** ***Rinvoq***	Upadacitinib hemihydrate [23]	AbbVie Inc. [24]	18th August 2025	rheumatoid arthritis, psoriatic arthritis, atopic dermatitis, ulcerative colitis, Crohn's disease, and ankylosing spondylitis	8.3 billion	Tofacitinib, baricitinib
**Pitolisant/** ***Wakelix***	Pitolisant hydrochloride [25]	Bioprojet [26]	27th August 2025	to treat excessive daytime sleepiness in narcolepsy	868 million	sodium oxybate, Modafinil
**Etrasimod/** ***Velsipity***	Etrasimod arginine [27]	Arena Pharmaceuticals, Inc. [28]	16th September 2025	ulcerative colitis	Not available	Ozanimod

**Erdafitinib/** ***Erdanat***	Erdafitinib (free base) [29]	Janssen Pharmaceutica NV [30]	1st October 2025	metastatic or locally advanced urothelial carcinoma	208 million	Dabogratinib, Pemigatinib
**Vorasidenib/** ***Voranigo***	Vorasidenib (free base) [31]	Agios Pharmaceuticals, Inc. [32]	15th October 2025	astrocytoma or oligodendroglioma with a susceptible isocitrate dehydrogenase-1 (IDH1)	Not available	Safusidenib
aSales figures were retrieved from publicly available sources, including company annual reports and pharmaceutical market databases						

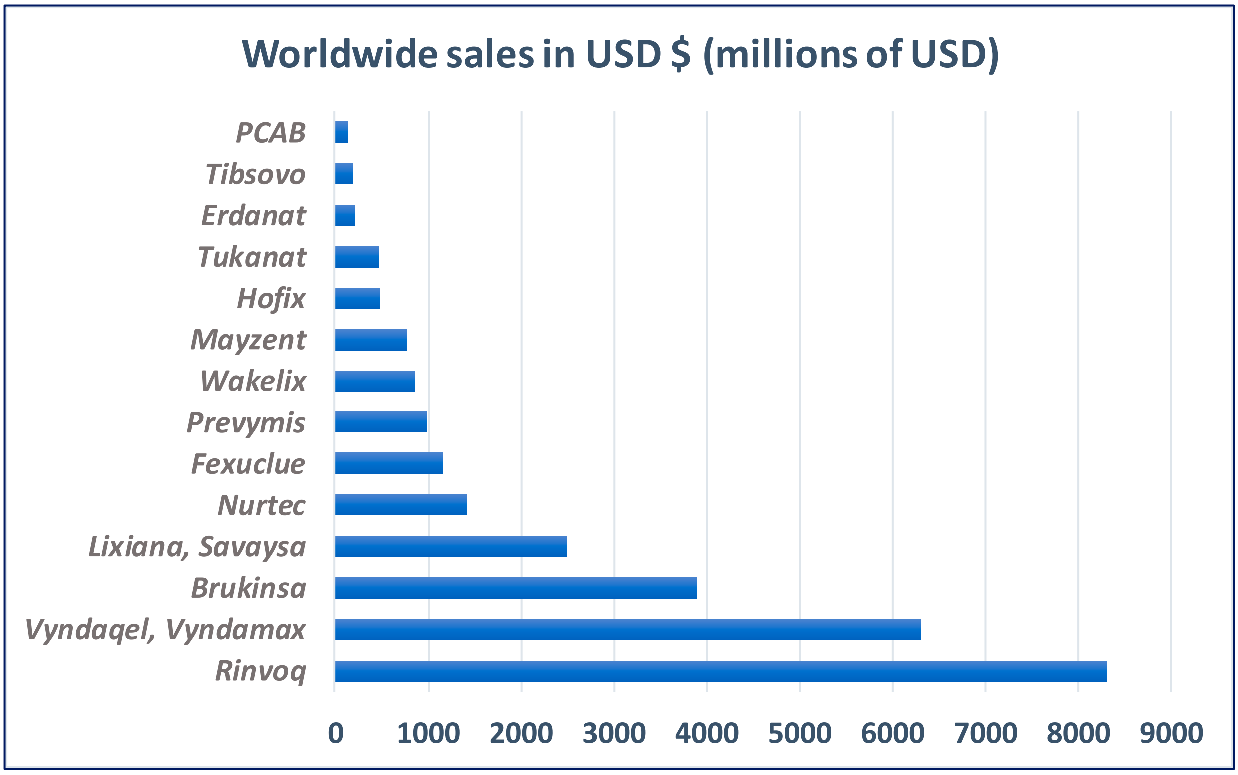

Figure S1. Worldwide sales (millions of USD) in 2025 for CDSCO-approved drugs described in the paper.

COMPUTATIONAL PART

- Rowan Scientific's calculation of isosurface [33]

In accordance with standard reporting guidelines, we detail the complete computational protocol from initial structure and ligand preparation to the generation of interaction maps, ensuring a clear distinction between experimental data and modeling predictions while acknowledging that identified contacts do not constitute direct evidence of binding affinity or pharmacological activity.
In Rowan Scientific's computational chemistry platform, isosurfaces are 3D visualizations used to map and interpret a molecule's electronic distribution, spatial properties, and reactive areas.
A workflow is herein presented:

1. Ligand upload and DFT settings

• Input Submission: The structure was uploaded to the Rowan Web Interface via SMILES strings.

• Computational Specification: A Geometry Optimization task was conducted using an appropriate level of theory. (A Density Functional Theory (DFT) with the range-separated hybrid functional ωB97X).

2. Orbitals and electronic property calculations

Once the optimized geometry was established, the target surface data was computed:
• Workflow Selection: A New Orbitals Calculation workflow was initiated on the optimized coordinates.
• Data Processing: Rowan utilizing underlying engines (such as PySCF or Psi4) computed the molecular electronic structure, yielding the molecular orbitals, the total electron density (ρ), and the molecular electrostatic potential (ESP). For open-shell radical systems, spin density matrix elements were also evaluated.

3. 3D Isosurface visualization & tuning

The computed volumetric grid data was rendered into 3D isosurfaces directly within the browser interface under the Isosurfaces tab:
• Electron Density Surfaces: To map the overall structural boundary (molecular envelope), points of equal electron density were connected to form the 3D surface.
• Cutoff Threshold Adjustment: To accurately capture specific molecular phenomena, the isovalue (cutoff) parameter was explicitly defined:
• Molecular Shape/Volume: A standard cutoff in the range of 0.01 to 0.05 e/Bohr^3 was applied to gauge the spatial extent of the electron cloud and molecular volume.
• Bond Density: A higher cutoff in the range of 0.1 to 0.15 e/Bohr^3 was isolated to visualize areas of high-order covalent bonding localized between atom pairs.
• Electrostatic Potential (ESP) Mapping: To analyze regional electrostatics, electrophilic/nucleophilic sites, or non-covalent interactions (e.g., σ-holes), the total electron density isosurface was mapped and colored by the ESP value. By convention, regions of negative electrostatic potential (electron-rich zones, such as lone pairs) were represented in red, while areas of positive electrostatic potential (electron-poor zones) were rendered in blue.

- PoseEdit [34]

PoseEdit automatically produces high-quality 2D diagrams of intermolecular interactions in 3D binding sites calculated from ligands in complex with proteins. It is a visualization tool that relies heavily on underlying computational calculations.
To visualize and analyze the specific binding modes, 2D protein-ligand interaction diagrams were generated using PoseEdit via the ProteinsPlus web server (Center for Bioinformatics, University of Hamburg). The underlying interaction model automatically calculated intermolecular contacts (such as hydrogen bonds, π-stacking, and hydrophobic interactions) using geometric criteria based on the raw 3D docking coordinates. PoseEdit was subsequently utilized as an interactive layout manager to optimize the visual presentation.

REFERENCES:

1. Thakkar, K.; Solanki, R.; Patel, R. Ultrasensitive Liquid Chromatography Triple Quadrupole Mass Spectrometry Detection and Quantification of *N* -Nitroso-Meglumine in Tafamidis Meglumine Capsules Using Electrospray Ionization. *Sep. Sci. PLUS* **2025**, *8*, e70091, doi:10.1002/sscp.70091.
2. Girard, K.P.; Jensen, A.J.; Jones, K.N. Crystalline Solid Forms of 6-Carboxy-2-(3,5-Dichlorophenyl)-Benzoxazole. US Patent US9770441B1, 26 September 2017.
3. Kim, E.S. Letermovir: First Global Approval. *Drugs* **2018**, *78*, 147–152, doi:10.1007/s40265-017-0860-8.
4. Cao, M.; Chen, D.; Huang, Z.; Chen, Y.; Jin, F.; Yang, S.; Zheng, B.; Gao, Q. Crystal Form a of Fexuprazan Hydrochloride, Preparation Method Therefor, and Use Thereof. WO Patent WO2024067844A1, 4 April 2024
5. Wang, T.; Hua, Y.; Deng, F.; Wen, R.; Zhang, H.; Li, C.; Liu, Y.; Cheng, M. Scalable Synthesis of the Key Fexuprazan Intermediate and Investigation of a Reaction Mechanism. *ACS Omega* **2024**, *9*, 37942–37952, doi:10.1021/acsomega.4c04507.
6. Rashid, M.A.; Muneer, S.; Mendhi, J.; Sabuj, M.Z.R.; Alhamhoom, Y.; Xiao, Y.; Wang, T.; Izake, E.L.; Islam, N. Inhaled Edoxaban Dry Powder Inhaler Formulations: Development, Characterization and Their Effects on the Coagulopathy Associated with COVID-19 Infection. *Int. J. Pharm.* **2021**, *608*, 121122, doi:10.1016/j.ijpharm.2021.121122.
7. Stacy, Z.A.; Call, W.B.; Hartmann, A.P.; Peters, G.L.; Richter, S.K. Edoxaban: A Comprehensive Review of the Pharmacology and Clinical Data for the Management of Atrial Fibrillation and Venous Thromboembolism. *Cardiol. Ther.* **2016**, *5*, 1–18, doi:10.1007/s40119-016-0058-2.
8. Istoc, M.; Chaudhry, B.A.; Christensen, R.H.; Al-Khazali, H.M.; Ashina, M.; Karlsson, W.K.; Ashina, H. Rimegepant for the Acute Treatment of Migraine: A Systematic Review and Meta-Analysis. *J. Headache Pain* **2026**, *27*, 174, doi:10.1186/s10194-026-02388-x.
9. Coric, V.; Croop, R. Rimegepant for CGRP Related Disorders. US Patent US11083724B2, 10 August 2021.
10. Talwani, R.; Temesgen, Z. Doravirine: A New Non-Nucleoside Reverse Transcriptase Inhibitor for the Treatment of HIV Infection. *Drugs Today Barc. Spain 1998* **2020**, *56*, 113–124, doi:10.1358/dot.2020.56.2.3109966.
11. Panmai, S.; Tatavarti, A.; Farrington, A.M.; Biyyala, V.; Allain, L.R.; Nefliu, M.; Klinzing, G.R.; Ren, J.; Lamm, M. Pharmaceutical Compositions Containing Doravirine, Tenofovir Disoproxil Fumarate and Lamivudine. US Patent US10603282B2, 31 March 2020.
12. Olson, D.; Taylor, J.; Willis, K.; Hensley, K.; Allred, S.; Zaval, M.; Farr, L.; Thurman, R.; Jain, N.; Hein, R.; et al. HER2-Selective and Reversible Tyrosine Kinase Inhibitor Tucatinib Potentiates the Activity of T-DM1 in Preclinical Models of HER2-Positive Breast Cancer. *Cancer Res. Commun.* **2023**, *3*, 1927–1939, doi:10.1158/2767-9764.CRC-23-0302.
13. Lyssikatos, J.P.; Greschuk, J.M.; Marmsäter, F.P.; Zhao, Q.; Hennings, D.D.; Liu, W. N4-Phenyl-Quinazoline-4-Amine Derivatives and Related Compounds as ErbB Type I Receptor Tyrosine Kinase Inhibitors for the Treatment of Hyperproliferative Diseases. US Patent US8648087B2, 11 February 2014.
14. Čermáková, L.; Hofman, J.; Laštovičková, L.; Havlíčková, L.; Šprіngrová, I.; Novotná, E.; Wsól, V. Bruton's Tyrosine Kinase Inhibitor Zanubrutinib Effectively Modulates Cancer Resistance by Inhibiting Anthracycline Metabolism and Efflux. *Pharmaceutics* **2022**, *14*, 1994, doi:10.3390/pharmaceutics14101994.
15. Wang, Z.; Guo, Y.; Shi, G.; Wang, L. Crystalline Form of (S)-7-(1-Acryloylpiperidin-4-Yl)-2-(4-Phenoxyphenyl)-4,5,6,7-Tetrahydropyrazolo[1,5-a]Pyrimidine-3-Carboxamide, Preparation, and Uses Thereof. US Patent US11884674B2, 30 January 2024
16. DrugBank. Siponimod Fumarate. Available online: https://go.drugbank.com/salts/DBSALT002866 (accessed on 23 October 2026).
17. Dymacek, B.; Partl, J.; Tesson, N.; Trilla Castano, M. Siponimod Salts and Cocrystals. WO Patent WO2022064007A1, 31 March 2022.
18. DiNardo, C.D.; Stein, A.S.; Stein, E.M.; Fathi, A.T.; Frankfurt, O.; Schuh, A.C.; Döhner, H.; Martinelli, G.; Patel, P.A.; Raffoux, E.; et al. Mutant Isocitrate Dehydrogenase 1 Inhibitor Ivosidenib in

Combination With Azacitidine for Newly Diagnosed Acute Myeloid Leukemia. *J. Clin. Oncol.* **2021**, *39*, 57–65, doi:10.1200/JCO.20.01632.
19. Fan, B.; Mellinghoff, I.K.; Wen, P.Y.; Lowery, M.A.; Goyal, L.; Tap, W.D.; Pandya, S.S.; Manyak, E.; Jiang, L.; Liu, G.; et al. Clinical Pharmacokinetics and Pharmacodynamics of Ivosidenib, an Oral, Targeted Inhibitor of Mutant IDH1, in Patients with Advanced Solid Tumors. *Invest. New Drugs* **2020**, *38*, 433–444, doi:10.1007/s10637-019-00771-x.
20. Gu, C.H. Pharmaceutical Compositions of Therapeutically Active Compounds. US Patent US9968595B2, 15 May 2018.
21. Kim, D.K.; Lee, K.-H.; Kim, S.; Kim, S.-J.; Lee, S.J.; Park, C.H.; Kim, B.-T.; Song, G.-S.; Moon, B.-S.; Ryu, S.-Y. Effects of Tegoprazan, a Novel Potassium-Competitive Acid Blocker, on Rat Models of Gastric Acid–Related Disease. *J. Pharmacol. Exp. Ther.* **2019**, *369*, 318–327, doi:10.1124/jpet.118.254904.
22. Qiu, X.; Zuo, Z.; Hu, L.; Zou, P.; Liu, W.; Chu, L.; Wang, P. Tegoprazan Analogues and Synthetic Method Thereof. WO Patent WO2021185192A1, 23 September 2021.
23. DrugBank. Upadacitinib Hemihydrate. Available online: https://go.drugbank.com/salts/DBSALT002885 (accessed on 23 October 2026).
24. Qiu, Y.; Huang, Y.; Zhou, D. Extended Release Upadacitinib Formulations. US Patent Application US20240316040A1, 26 September 2024.
25. DrugBank. Pitolisant Hydrochloride. Available online: https://go.drugbank.com/salts/DBSALT002888 (accessed on 23 October 2026).
26. Nithiyanandam, R.K.; Thota, S.P. A Pharmaceutical Composition of Pitolisant Hydrochloride and Their Process for the Preparation. WO Patent WO2023214431A1, 9 November 2023.
27. DrugBank. Etrasimod Arginine. Available online: https://go.drugbank.com/salts/DBSALT003430 (accessed on 23 October 2026).
28. Grundy, J.S.; Lee, C.A. Etrasimod for Use in Treating S1p1 Receptor-Associated Disorders in Combination with Hormone Treatment. WO Patent WO2023135506A1, 20 July 2023.
29. Loriot, Y.; Matsubara, N.; Park, S.H.; Huddart, R.A.; Burgess, E.F.; Houede, N.; Banek, S.; Guadalupi, V.; Ku, J.H.; Valderrama, B.P.; et al. Erdafitinib or Chemotherapy in Advanced or Metastatic Urothelial Carcinoma. N. Engl. J. Med. 2023, 389, 1961–1971
30. Saxty, G.; Murray, C.W.; Berdini, V.; Besong, G.E.; Hamlett, C.C.F.; Johnson, C.N.; Woodhead, S.J.; Rees, D.C.; Mevellec, L.A.; Angibaud, P.R.; et al. Pyrazolyl Quinoxaline Kinase Inhibitors. US Patent US8895601B2, 25 November 2014.
31. Barbato, M.I.; Barone, A.K.; Aungst, S.L.; Miller, C.P.; Ananthula, S.; Bi, Y.; Yang, Y.; Li, X.; Xiong, Y.; Fan, J.; et al. FDA Approval Summary: Vorasidenib for IDH-Mutant Grade 2 Astrocytoma or Oligodendroglioma Following Surgery. *Clin. Cancer Res. Off. J. Am. Assoc. Cancer Res.* **2025**, *31*, 4412–4418, doi:10.1158/1078-0432.CCR-25-1333.
32. Zenon D. KonteatisJaneta Popovici-MullerJeremy M. TravinsRobert ZahlerZhenwei CAIDing Zhou Therapeutically Active Compounds and Their Methods of Use.
33. Rowan Scientific. Https://Www.Rowansci.Com (Accessed 2026-10-22). Available online: https://www.rowansci.com.
34. Ehrt, C.; Schulze, T.; Graef, J.; Diedrich, K.; Pletzer-Zelgert, J.; Rarey, M. ProteinsPlus: A Publicly Available Resource for Protein Structure Mining. *Nucleic Acids Res.* **2025**, *53*, W478–W484, doi:10.1093/nar/gkaf377.
